# Synthesis of a Functionalized Bicyclo[3.2.1]Octane: A Common Subunit to Kauranes, Grayananes, and Gibberellanes

**DOI:** 10.1002/chem.202502441

**Published:** 2025-09-13

**Authors:** Nicolas Fay, Camil Benbouziyane, Cyrille Kouklovsky, Aurélien de la Torre

**Affiliations:** ^1^ Institut de Chimie Moléculaire et des Matériaux d'Orsay (ICMMO, UMR 8182), CNRS Université Paris‐Saclay Orsay 91405 France

**Keywords:** bicycles, cyclization, metathesis, multi‐step synthesis, natural products

## Abstract

Kauranes, grayananes, and gibberellanes are three important diterpenoid families. These natural products all share a bicyclo[3.2.1]octane skeleton, with oxidation at very specific positions. In this manuscript, we describe the synthesis of a bicyclo[3.2.1]octane building block, which could serve as a potential intermediate for the synthesis of natural products from these three families. A first approach relying on a 1,4‐sila‐Prins cyclization was first explored, which required the selective functionalization of dihydrocarvone (via C─H activation and selective oxidation). This strategy resulted in a dead end when a 1,2‐cyclization product was obtained. An alternative strategy relying on ring‐closing metathesis (RCM) allowed to successfully achieve the synthesis of the desired scaffold in 8 steps from cyclohexenone.

## Graphical abstract

A detailed account on the synthesis of a bicyclo[3.2.1]octane scaffold, common to the kaurane, grayanane, and gibberellane diterpenoid families, is reported. Our original strategy relying on a 1,4‐sila‐Prins cyclization proved unsuccessful. Learning from that, we designed an alternative strategy relying on ring‐closing metathesis (RCM), which ultimately led to the desired compound in 8 steps.

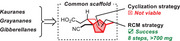

## 1 Introduction

**Scheme 1:**
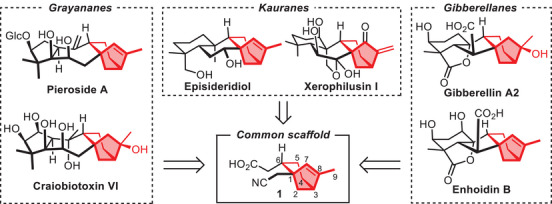
Structure of kauranes, grayananes, gibberellanes, and proposed common synthetic scaffold.

Kauranes are a broad family of diterpenoids encountered in the *Lamiaceae*, *Asteraceae*, *Annonaceae*, and *Euphorbiaceae* families.^[^ ^1^, ^2^ ^]^ These natural products display a wide range of biological activities, including antibacterial, anticancer, and antifungal, providing them with therapeutic potential. Kauranes have a tetracyclic structure with 6/6/6/5‐membered rings, and various possible oxidation patterns (Scheme 1). Their synthesis has attracted the attention of the organic chemistry community, with reports as early as 1962 by Ireland et al,^[^ ^3^ ^]^ and a continuous interest since then.^[^ ^4^, ^5^, ^6^ ^]^ Grayananes are a biosynthetically related family of natural products, originating from a^[^ ^1^, ^2^ ^]^‐rearrangement from the kaurane skeleton, which leads to a tetracycle with 5/7/6/5‐membered rings.^[^ ^7^ ^]^ Grayananes are mainly found in the *Ericaceae* plant family, which are commonly used in traditional medicine around the world. They display interesting antinociceptive, antifeedant, and antitumor activities, which prompted various groups to engage in their total synthesis,^[^ ^8^ ^]^ from early relay synthetic approaches by Matsumoto^[^ ^9^ ^]^ to recent strategies by Newhouse,^[^ ^10^ ^]^ Ding,^[^ ^11^ ^]^ Luo,^[^ ^12^ ^]^ Jia,^[^ ^13^, ^14^ ^]^ and Yang.^[^ ^15^ ^]^ The gibberellane family is another biosynthetically related diterpenoid family, originating from a different^[^ ^1^, ^2^ ^]^‐rearrangement from a functionalized kaurane precursor.^[^ ^16^ ^]^ They are produced by various plants, bacteria, and fungal species. The first synthesis of gibberellanes was reported by Mori at the same time as the early kaurane syntheses,^[^ ^17^ ^]^ but some gibberellanes were also recently synthesized by Dai and Fan.^[^ ^18^, ^19^ ^]^ These three natural product families display a broad diversity of structures owing to the multiple possible oxidation patterns.

Natural products from these three families share a bicyclo[3.2.1]octane scaffold, often named rings C and D. The synthesis of this scaffold represents a challenge which has stimulated the creativity of organic chemists for decades.^[^ ^20^ ^]^ Attracted by the prospect of reaching various natural products within these three different natural product families, we decided to design a common intermediate **1** having the shared bicyclo[3.2.1]octane scaffold. In order to introduce potential oxidation at C^7^, C^8^, or C^9^, we imagined an olefin at C^7^═C^9^, which could undergo Mukaiyama hydration^[^ ^21^ ^]^ or epoxidation/elimination.^[^ ^22^ ^]^ Moreover, we wanted to have two orthogonal chemical handles, which would allow to build the rest of the scaffolds. Thus, we designed this intermediate with a carboxylic acid and a nitrile group, which can be functionalized chemoselectively. In this manuscript, we describe our efforts toward the synthesis of this scaffold.

## 2 Results and Discussion

**Scheme 2:**
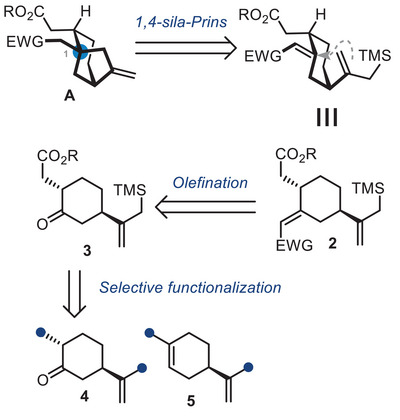
Proposed retrosynthesis of 1.

The key challenge is the formation of the bicyclo[3.2.1]octane scaffold containing the quaternary center C^1^ at the bridgehead position. Our original approach relied on a 1,4‐sila‐Prins cyclization reaction for the formation of **A**, which would have an exocyclic olefin allowing possible oxidation,^[^ ^21^ ^]^ from intermediate **2** having the cyclohexane core, an allylsilane, and a Michael acceptor (Scheme 2). We imagined that the Michael acceptor could be obtained from the corresponding ketone **3**, which we could trace back to either dihydrocarvone **4** or limonene **5**.

### 2.1 C─H Activation from Dihydrocarvone

**Scheme 3:**
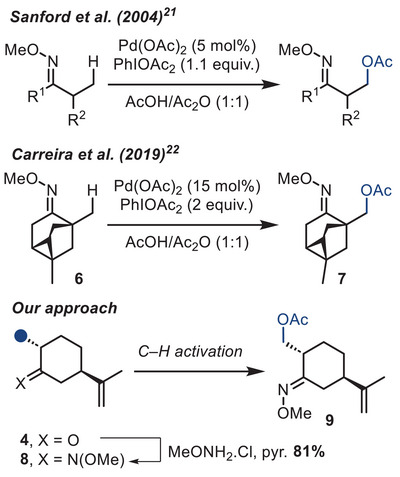
Oxime directed C─H acetoxylation.

In our initial approach, we consider valorizing natural dihydrocarvone **4**, readily available from commercial sources in enantioenriched form. The C─H activation method developed by Sanford in 2004, allowing the *β*‐functionalization of oximes (Scheme 3), seemed particularly suited for that.^[^ ^23^ ^]^ In particular, Carreira and coworkers had also applied this method to the functionalization of more complex 2‐methylcyclohexanone derivative **6** in the synthesis of the tricyclooctane core of trachylobane natural products.^[^ ^24^ ^]^ To test this approach, we prepared the *O*‐methyloxime derivative **8** from dihydrocarvone **4**, and submitted it to various C─H activation conditions.

Our first attempts were directly based on the reaction conditions described by Sanford et al., using Pd(OAc)_2_ as the catalyst and PhI(OAc)_2_ as the oxidant in a mixture of AcOH/Ac_2_O (Table 1). The reaction required heating at 100 °C to reach full conversion, although prolonged reaction times were detrimental to the reaction (entries 1–5). Increasing catalyst loading (entry 6) did not significantly improve the yield, while increasing the amount of oxidant led to over‐oxidation products (entry 7). The best result was obtained using Pd(dba)_2_ as the palladium source (entry 9), while heating with microwave irradiation or changing the solvent to acetonitrile led to decomposition (entries 8 and 10).

**Table 1:** Summary of C─H acetoxylation attempts.

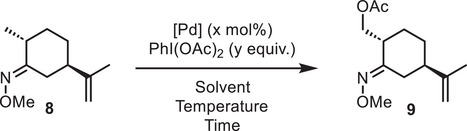							
Entry	[Pd] [x mol%]	y	Solvent	Temperature	Time	Conversion [%]	Yield [%]^[^ ^a^ ^]^
1	Pd(OAc)_2_ (5 mol%)	1.1	AcOH/Ac_2_O (1:1)	70 °C	3 hours	20	10
2	Pd(OAc)_2_ (5 mol%)	1.1	AcOH/Ac_2_O (1:1)	70 °C	16 hours	80	18
3	Pd(OAc)_2_ (5 mol%)	1.1	AcOH/Ac_2_O (1:1)	100 °C	3 hours	100	ND
4	Pd(OAc)_2_ (5 mol%)	1.1	AcOH/Ac_2_O (1:1)	100 °C	1.5 hours	100	21^[b]^
5	Pd(OAc)_2_ (5 mol%)	1.1	AcOH/Ac_2_O (1:1)	100 °C	1 hour	100	26
6	Pd(OAc)_2_ (20 mol%)	1.1	AcOH/Ac_2_O (1:1)	100 °C	1 hour	100	22
7	Pd(OAc)_2_ (5 mol%)	2	AcOH/Ac_2_O (1:1)	100 °C	1 hours	100	ND
8	Pd(OAc)_2_ (5 mol%)	1.1	AcOH/Ac_2_O (1:1)	100 °C^[c]^	15 minutes	100	ND
9	Pd(dba)_2_ (5 mol%)	1.1	AcOH/Ac_2_O (1:1)	100 °C	1 hour	100	32
10	Pd(dba)_2_ (5 mol%)	1.1	MeCN	100 °C	1 hour	100	ND

^[a]^ NMR yield determined using 1,3,5‐trimethoxybenzene as the internal standard.

^[b]^ Isolated yield.

^[c]^ Microwave, 200 W.

In all cases, although the conversion was complete, product **9** was obtained in low yield. We assume that this is due to the presence of an olefin as well as inherently more reactive allylic C─H, although no other product could be isolated. It should be noted that neither Sanford nor Carreira had tested this reaction on substrates bearing olefins. This limitation led us to abandon the C─H activation approach.

### 2.2 Hydrocyanation Reaction from a Limonene Derivative

**Scheme 4:**
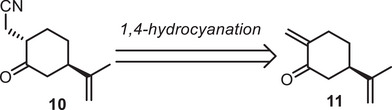
Proposed 1,4‐hydrocyanation.

As an alternative approach, we imagined a key 1,4‐hydrocyanation reaction to introduce a nitrile moiety on an enone derivative **11** (Scheme 4). The enone could itself be traced back to commercially available limonene **5**, also accessible in enantioenriched form.

To test this approach, we converted limonene **5** into perillyl alcohol **13** through an epoxidation/elimination sequence developed by Evans.^[^ ^25^ ^]^ Perillyl alcohol was then oxidized under Swern conditions. The resulting enone **14** was very unstable and had to be engaged directly into the key 1,4‐hydrocyanation. As the hydrocyanation product was unstable and the reaction was reversible, we had to further hydrolyze the nitrile into the corresponding carboxylic acid **15**. Despite all our attempts, the product was obtained at best with 18% yield. This was due to the formation of side product **16** arising from a hetero‐Diels‐Alder dimerization, which had previously been described by Hayes in the synthesis of cymbodiacetal.^[^ ^26^ ^]^

**Scheme 5:**
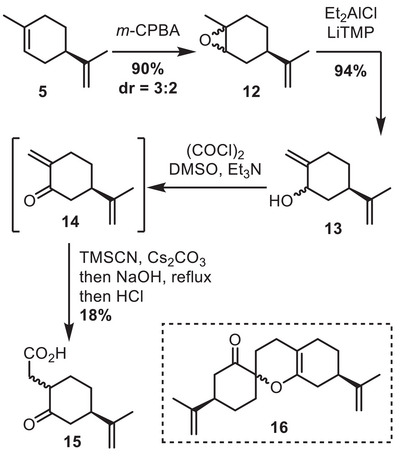
Attempts toward the 1,4‐hydrocyanation approach.

The poor yields obtained in this reaction led us to abandon this approach, and explore alternative strategies not relying on the chiral pool (Scheme 5).

### 2.3 Efforts toward the Formation of the Bicyclo[3.2.1]Octane

**Scheme 6:**
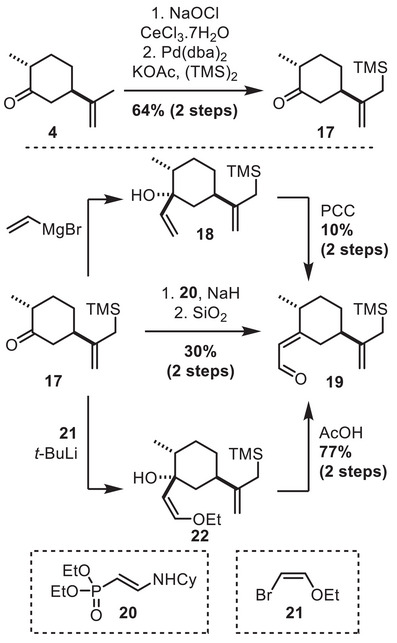
Preparation of a model substrate for the 1,4‐sila‐Prins reaction.

Our strategy relied on a key 1,4‐Sakurai reaction for the bicyclo[3.2.1]octane construction. Although challenging due to the poor orbital alignment inherent to this 5‐*enolendo‐exo‐trig* cyclization mode,^[^ ^27^ ^]^ some precedents in the literature indicated that a similar cyclization could proceed with the right Lewis acid.^[^ ^28^, ^29^ ^]^ To test this approach, a model substrate for this cyclization was elaborated from dihydrocarvone **4**. Allylic chlorination followed by palladium‐catalyzed silylation allowed to introduce the allylsilane moiety (Scheme 6). Following this, ketone **17** underwent a vinyl Grignard addition, followed by chromium‐mediated Babler‐Dauben oxidative rearrangement. However, this sequence led to poor yield due to undesired protodesilylation (see Supporting Information for detail). As an alternative, we tested Nagata homologation using reagent **20**. Again, the product **19** was obtained in unsatisfying yield. Finally, addition of the organolithium reagent deriving from **21** followed by acid hydrolysis provided **19** in good yield. A careful choice of acidic conditions was necessary in order to minimize the competitive protodesilylation in this process (see Supporting Information for more detail).

**Scheme 7:**
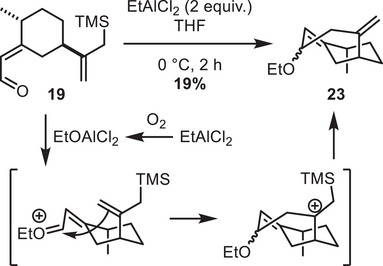
Unexpected 1,2‐sila‐Prins reaction.

With compound **19** in hand, we started to explore the key 1,4‐sila‐Prins cyclization. While most conditions afforded protodesilylation or degradation of **19** (see the Supporting Information for more detail), the use of EtAlCl_2_ in THF led to the unexpected formation of bicyclo[4.3.1]decane **23** (Scheme 7). We rationalize this by the presence of trace amounts of oxygen in THF, which could oxidize EtAlCl_2_ into EtOAlCl_2_, promoting the formation of an oxonium, which would then undergo a 1,2‐sila‐Prins cyclization. This observation indicates that 1,2‐sila‐Prins is geometrically favored over 1,4‐addition on this scaffold, and prompted us to explore other functionalities to achieve the desired cyclization.

**Scheme 8:**
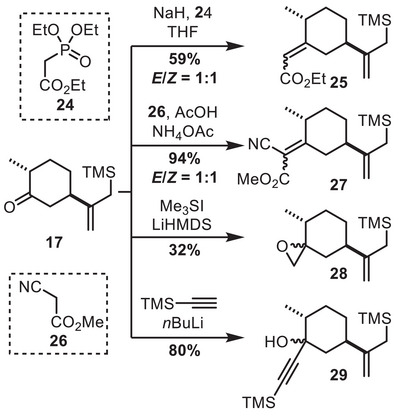
Preparation of alternative cyclization substrates.

In order to explore other substrates for cyclization in which formation of the bicyclo[4.3.1]decane would not be a competitive pathway, we synthesized Michael acceptors **25** and **27** by Horner‐Wadsworth‐Emmons and Knoevenagel reactions, respectively, from **17** (Scheme 8). Moreover, epoxide **28** was prepared by a Corey‐Chaykovsky reaction, and propargyl alcohol **29** was obtained by addition of lithiated TMS‐acetylene. All these products were obtained as diastereomeric mixtures. However, all attempts to cyclize these alternative substrates led either to decomposition or protodesilylation (see Supporting Information for more details).

The challenges associated with this cyclization can be explained by the cyclization mode, which is close to a 5‐*enolendo*‐*exo*‐*trig*, unfavored according to Baldwin's rules modification relative to enolates.^[^ ^27^ ^]^ This can be rationalized by a poor orbital overlap between the two *π*‐systems due to geometrical constrains.^[^ ^30^, ^31^ ^]^ After reaching these multiple dead ends, we decided to turn away from the cyclization approach and explore a different strategy based on ring‐closing metathesis (RCM).

### 2.4 Synthesis of the Bicyclo[3.2.1]Octane Framework by Ring‐closing Metathesis

**Scheme 9:**
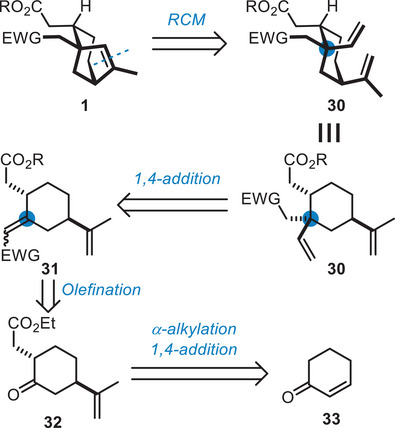
Revised strategy relying on RCM.

A revised strategy relying on RCM was designed, in which **1** would be obtained from **30** having the two olefins appended for the key RCM (Scheme 9). The quaternary stereogenic center would be formed by a diastereoselective 1,4‐addition from Michael acceptor **31**, which would in turn be formed from functionalized cyclohexanone **32**. For a fast preparation of the functionalized cyclohexanone intermediate **32**, we considered a two‐step approach from cyclohexenone **33** involving *α*‐alkylation of the ketone and 1,4‐addition. Both orders of steps can be envisaged: achieving the *α*‐alkylation first could simplify the regioselectivity question in the enolate formation, while achieving the 1,4‐addition first would allow an enantioselective synthesis using the conditions developed by Schmalz.^[^ ^32^ ^]^

**Scheme 10:**
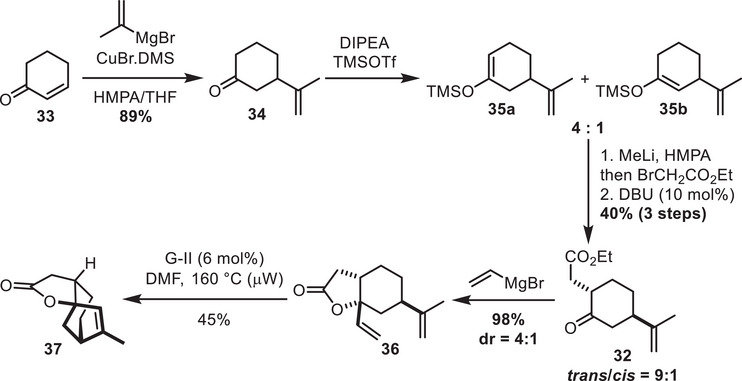
Functionalization of cyclohexanone by performing first the conjugate addition.

Cyclohexenone **33** underwent a Cu‐catalyzed 1,4‐addition to introduce the isopropenyl function (Scheme 10). It should be noted that the enantioselective version of this reaction was reported by Schmalz with very high levels of enantioselectivity.^[^ ^32^ ^]^ As demonstrated by Vanderwal and coworkers,^[^ ^33^ ^]^ soft enolization could allow the formation of the enol ether **35** with a decent regioselectivity (for more details on the enolization conditions explored, see the Supporting Information). Treatment of this enol ether with MeLi and trapping the resulting lithium enolate with ethyl‐*α*‐bromoacetate followed by DBU‐mediated epimerization ultimately led to the desired functionalized cyclohexanone **32**. To further test the viability of the RCM approach, we subjected ketone **32** to vinyl Grignard addition. The resulting lactone **36** could cyclize in the presence of Grubbs 2^nd^ generation catalyst under microwave conditions, leading to the tricyclic structure **37**.

**Scheme 11:**
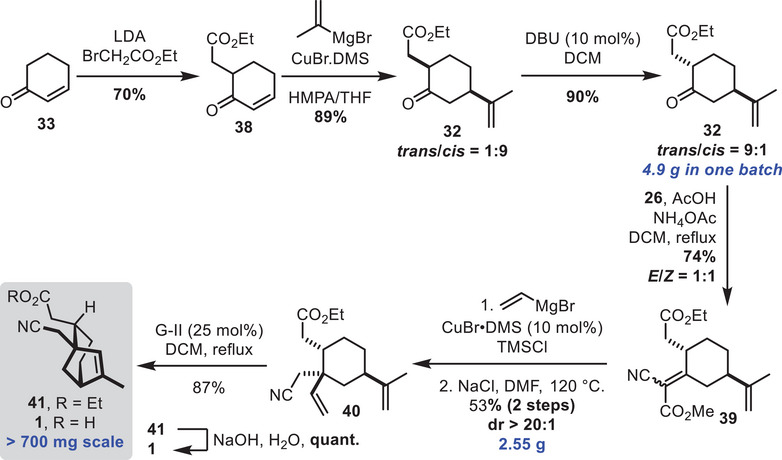
Formation of the bicyclo[3.2.1]octane by ring‐closing‐metathesis.

A faster way to prepare **32** was achieved by reversing the step order. Starting from cyclohexenone **33**, *α*‐alkylation with ethyl‐*α*‐bromoacetate led to the formation of **38**, which further underwent a Cu‐catalyzed 1,4‐addition to introduce the isopropylidene moiety (Scheme 11). This led to product **32** with undesired *cis*‐diastereoselectivity. The desired *trans* arrangement could be obtained by simply treating the product with DBU to epimerize the stereogenic center *α* to the ketone. More importantly, this sequence could be easily scaled‐up to multigram scale. Then, the ketone precursor **32** underwent a Knoevenagel condensation. The resulting *α*,*β*‐unsaturated cyanoester **39** was subjected to a Cu‐catalyzed 1,4 addition using a vinyl Grignard reagent. To simplify the diasteromeric mixture, the methyl ester was decarboxylated under Krapcho conditions (see Supporting Information for details). The polysubstituted cyclohexane **40** having the required vinyl moieties for a ring closing metathesis was obtained with excellent diastereoselectivity. Following that, the RCM went smoothly and the desired bicyclo[3.2.1]octane **41** was isolated in good yield. The ester moiety could further be hydrolyzed without affecting the nitrile, thus leading to the target fragment **1**. Moreover, this synthesis could be easily scaled up to 700 mg.

## 3 Conclusion

To conclude, we have reported the synthesis of a functionalized bicyclo[3.2.1]octane scaffold, a skeleton common to various natural products from the kaurane, grayanane, and gibberellane families. This compound possesses two orthogonal functionalities (nitrile and carboxylic acid), which could be used for further elaboration of the above‐mentioned structures. Our first approaches relying on the chiral pool (starting from dihydrocarvone or limonene) proved unsuccessful. Moreover, the original plan of forming the bicyclo[3.2.1]octane by a 1,4‐sila‐Prins cyclization also failed, due to geometrical constrains, which could not be overcome. Ultimately, we successfully prepared the bicyclo[3.2.1]octane framework by a RCM strategy. The application of this building block to the total synthesis of natural products from the three aforementioned diterpenoid families will be studied in the future in our lab.

## Supporting Information

The authors have cited additional references within the Supporting Information.^[^ ^34^, ^35^, ^36^, ^37^, ^38^, ^39^, ^40^, ^41^ ^]^

## Conflict of Interest

The authors declare no conflict of interest.

## Supporting information



Supporting Information

## Data Availability

The data that support the findings of this study are available in the supplementary material of this article.
