# Supplementary material for: Synthesis of a Functionalized Bicyclo[3.2.1]Octane: A Common Subunit to Kauranes, Grayananes, and Gibberellanes
Source: Chemistry. 2025 Sep 13;31(64):e02441. doi: 10.1002/chem.202502441 (PMC12624312; doi:10.1002/chem.202502441)
Supplement: Supplementary file 1 — Supporting Information [file CHEM-31-e02441-s001.pdf]

Supporting Information

Synthesis of a Functionnalized Bicyclo[3.2.1]octane: a Common Subunit to Kauranes, Grayananes and Gibberellanes

Nicolas Fay, Camil Benbouziyane, Cyrille Kouklovsky and Aurélien de la Torre*

Institut de Chimie Moléculaire et des Matériaux d'Orsay (ICMMO), Université Paris-Saclay, CNRS, 17 avenue des Sciences, 91405 Orsay, France

Supplementary tables and figures 2

Scheme 1. Attempted strategies for the synthesis of ester 32. 2

Scheme 2. 1,4-sila-Prins cyclization to form bridgehead bicyclo[3.2.1]octane 23' 3

Table 1: Enal formation in acidic conditions 4

Table 2: Control of enolization 4

Table 3. Attempted to 1,4-sila-Prins cyclization 5

Table 4: Optimization of Cu-catalyzed 1,4 addition / Krapcho decarboxylation 6

Scheme 3. Synthesis of (±) acid bicyclo[3.2.1]octane 1 7

Experimental section 8

General information 8

Experimental data and procedures 9

Copy of NMR spectras 34

References 54

Supplementary tables and figures

Scheme 1. Attempted strategies for the synthesis of ester 32.

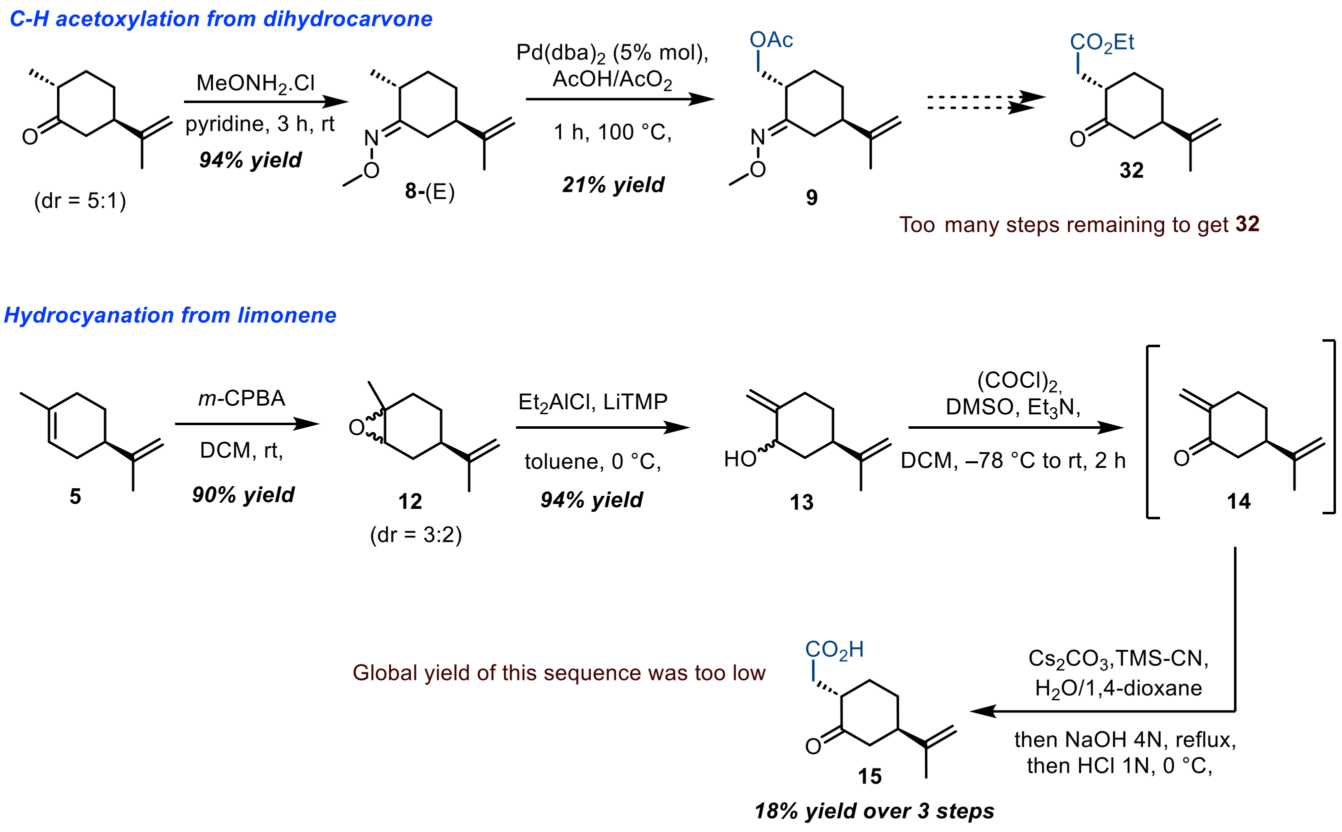

Scheme 2. 1,4-sila-Prins cyclization to form bridgehead bicyclo[3.2.1]octane 23'

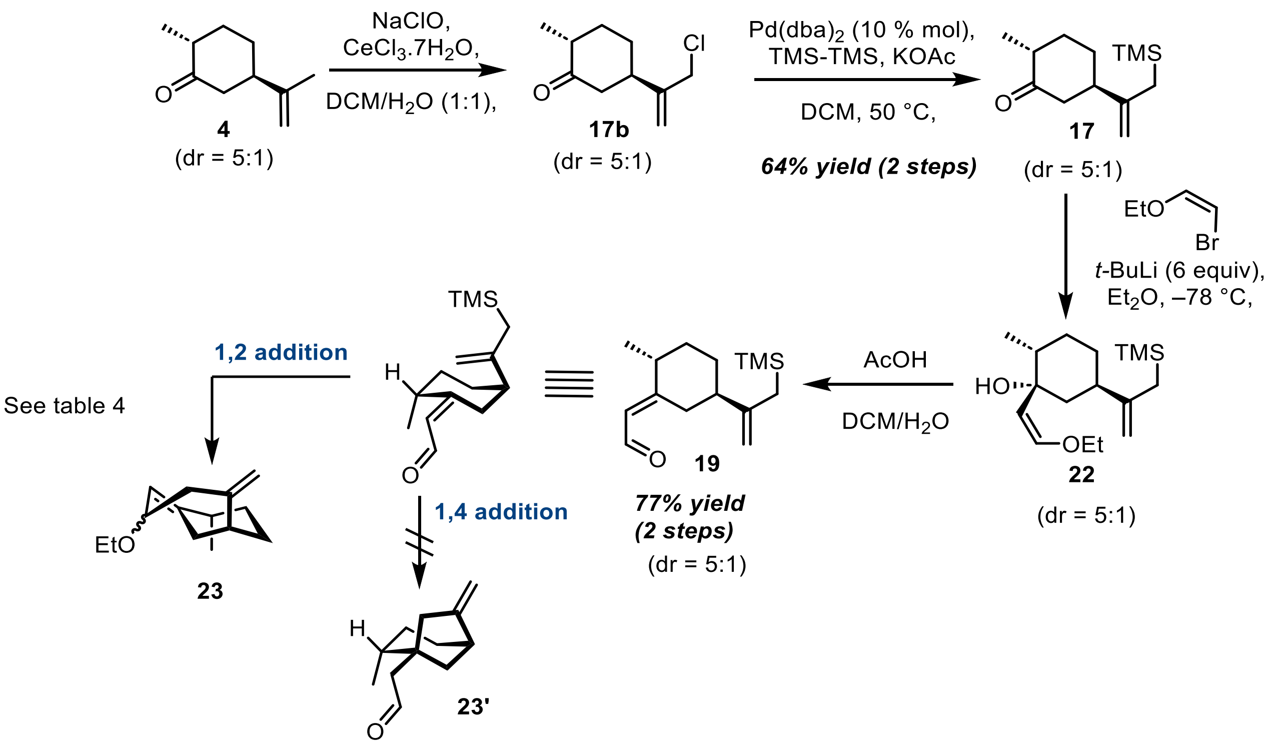

Table 1: Enal formation in acidic conditions

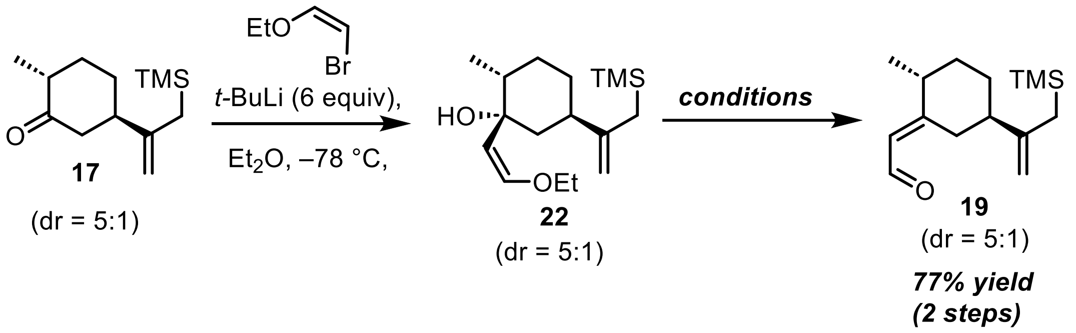

entry	conditions	results[a]
**1**	SiO_2, DCM/H_2O, (2:1), rt, 24 h	No reaction
**2**	Oxalic acid, DCM/H_2O (2:1), rt, 24 h	Protodesilylation, no complete conversion
**3**	AcOH, DCM/H_2O, (2:1), rt, 24 h	No protodesilylation, only target product

[a]Determined by ^1H NMR of crude product.

Table 2: Control of enolization

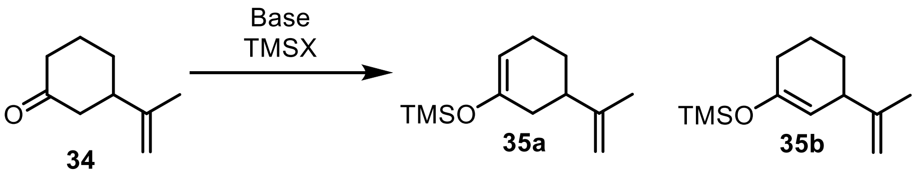

entry	base	TMSX	solvant	temperatures	ratio[b] (**35a** : **35b**)
**1**	DIPEA	TMSOTf	DCM	–78 °C	4 : 1
**2**	DIPEA[e]	TMSOTf	DCM	–78 °C	4 : 1
**3**	DIPEA	TMSOTf	DCM	–90 °C	4 : 1
**4**	DIPEA	TBSOTf	DCM	–78 °C	4 : 1
**5**	TMP	TMSOTf	DCM	–78 °C	2 : 1
**6**	LiTMP	TMSCl	THF	–78 °C	2 : 1
**7**	PMP[d]	TMSOTf	DCM	–78 °C	2.5 : 1
**9**	TMEDA	TMSOTf	DCM	–78 °C	No reaction
**10**	LDA	TMSCl	THF	–78 °C	2 : 1

[a][ketone] = 0,3 mol/L, base (3.5 equiv), Lewis acid (3 equiv). [b]Ratio was determined by NMR ^1H in crude. [d]PMP = 1,2,2,6,6-pentaméthylpipéridine. [e]DIPEA add to a mixture of TMSOTf and starting material instead of TMSOTf add to a mixture of DIPEA and starting material.

Table 3. Attempted to 1,4-sila-Prins cyclization

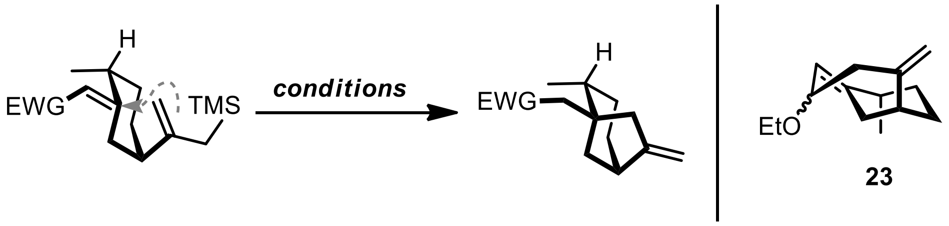

23

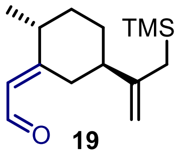

19

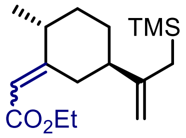

25

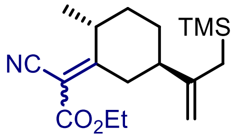

27

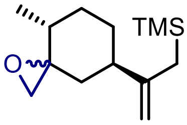

28

entry	substrate	conditions	results
**1**		$EtAlCl_2$ (2 equiv), toluene, 0 °C, 30 min	protodesilylation
**2**		$EtAlCl_2$ (2 equiv), hexane, 0 °C, 2 h	decomp
**3**		$EtAlCl_2$ (2 equiv), DCM, 0 °C, 1 h	protodesilylation
**4**		$EtAlCl_2$ (2 equiv), THF, 2 h, 0 °C	**23** (19%)
**5**	**19**	$EtAlCl_2$ (2 equiv), $AlMe_3$ (1 equiv), THF, –78 °C to –10 °C,	protodesilylation
**6**		$EtAlCl_2$ (2 equiv), $AlMe_3$ (1 equiv), toluene, –78 °C to rt	decomp
**7**		$EtAlCl_2$ (2 equiv), $AlMe_3$ (1 equiv), THF, –78 °C to rt, 24 h	Isomerization + protodesilylation
**8**		$TiCl_4$ (0.5 equiv), DCM, –78 °C, 30 min	isomerization
**9**		TMSOTf (1.1 equiv), DCM, –78 °C, 2 h then –30 °C, 1 h	protodesilylation
**10**	**25**	$TiCl_4$ (2 equiv), DCM, –78 °C, 30 min	decomp
**11**		$EtAlCl_2$ (2 equiv), THF, 0 °C, 2 h	decomp
**12**	**27**	$EtAlCl_2$ (2 equiv), THF, 0 °C, 2 h	protodesilylation
**13**		$EtAlCl_2$ (2 equiv), toluene, 0 °C, 2 h	protodesilylation
**14**	**28**	$EtAlCl_2$ (2 equiv), hexane, 0 °C, 4 h	protodesilylation
**15**		TBAF (1 equiv), DMF, rt, 3 h	protodesilylation

All reactions were performed under argon.

Table 4: Optimization of Cu-catalyzed 1,4 addition / Krapcho decarboxylation

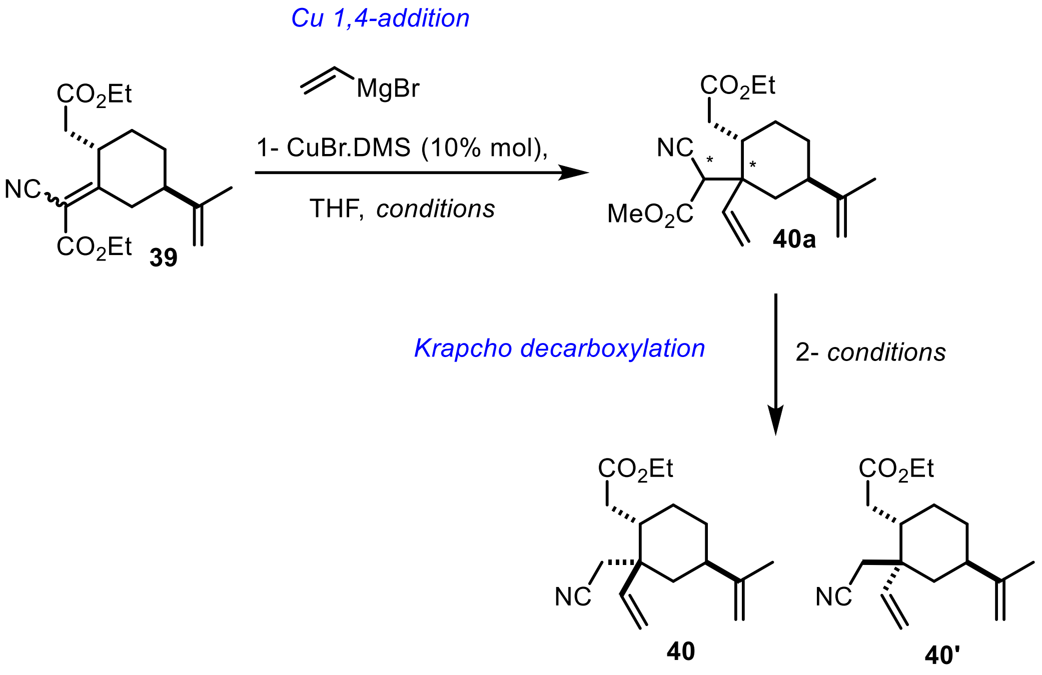

Entry	*1,4-addition*[a]	*Krapcho*	*dr* (**40** : **40'**)[b]	*global yields (%)*
**1**	0 °C to rt	LiCl, DMSO, 150 °C, 2 h	3 : 2	5
**2**	–10 °C	NaCl, DMSO, 150 °C, 2 h	3 : 1	39
**3**	–25 °C	NaCl, DMSO/H_2O, 150 °C, 2 h	12 : 1	13
**4**	TMSCl, –20 °C	NaCl, DMF, 150 °C, 3 h	25 : 1	39
**5**	TMSCl, –20 °C	NaCl, DMF, 120 °C, 12 h	25 : 1	53

[a]CuBr. DMS (10 mol%), THF, 2h. [b] dr was measured by ^{1}H NMR of the crude product.

Scheme 3. Synthesis of (±) acid bicyclo[3.2.1]octane 1

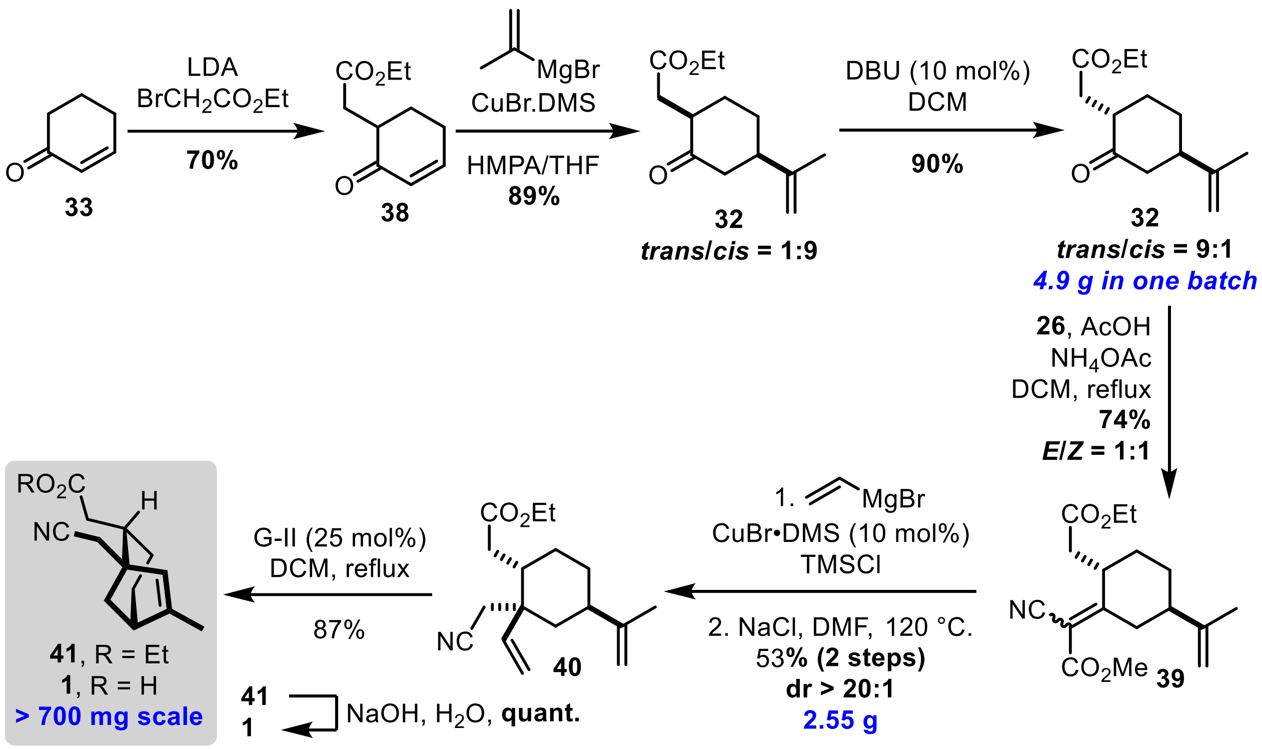

Experimental section

General information

All chemicals and solvents were purchased from commercial suppliers and used without further purification unless otherwise noted. Dichloromethane (DCM) and toluene were distilled with calcium hydride under an atmosphere of argon, while tetrahydrofuran (THF) and diethylether (Et_2O) were distilled with sodium and benzophenone under an atmosphere of argon. When heating the reaction mixture was necessary, either oil bath or sand bath were used. Analytical TLC was performed with silica gel 60 F254 plates and revealed either with PMA (Dissolve 10 g of phosphomolybdic acid in 100 mL of absolute ethanol.), $KMnO_4$ (Dissolve 1.5 g of $KMnO_4$, 10 g K_2CO_3, and 1.25 mL 10% NaOH in 200 mL water. A typical lifetime for this stain is approximately 3 months.) or UV. Column chromatography was performed on silica gel 60 M (0.04-0.063 mm) using appropriate solvents as eluent. Molecular sieves (4Å, white powder) were purchased from Alfa Aesar and further activated before use. NMR spectra were recorded on Bruker AVANCE I DRX-300 spectrometer (1H NMR at 300 MHz and 13C NMR at 75 MHz), Bruker AVANCE I AM-360 spectrometer (1H NMR at 360 MHz and 13C NMR at 90 MHz), or Bruker AVANCE III DRX-400 spectrometer (1H NMR at 400 MHz and 13C NMR at 100 MHz). Proton chemical shifts were referenced relative to the residual solvent peak (δ = 7.26 ppm for $CDCl_3$, 2.5 ppm for DMSO-*d6*). Carbon chemical shifts were referenced relative to deuterated solvent (δ = 77.16 ppm for $CDCl_3$, 39.52 ppm for DMSO-*d6*). Data for 1H NMR were reported as follows: chemical shift (ppm), multiplicity (*s* = singlet, *d* = doublet, *t* = triplet, *q* = quartet, *m* = multiplet, *br* = broad), coupling constants (Hz), integration. Data for 13C NMR were reported in chemical shift (ppm). ESI technique was used for the high-resolution mass (HRMS) measurements using Bruker Daltonics MicroTOF-Q spectrometer.

Experimental data and procedures

Dihydrocarvone oxime 8

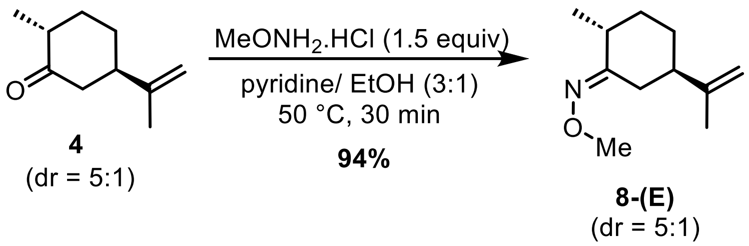

A solution of dihydrocarvone **4** (5.5 mmol, 0.840 g) and hydroxylamine hydrochloride (8.3 mmol, 0.70 g, 1.5 equiv.) in pyridine/EtOH (9 mL:3 mL) was stirred at room temperature for 3h. Pyridine was removed under reduced pressure. The residue was diluted with diethyl ether and then washed with water. The organic phase was dried over $MgSO_4$, filtered, and evaporated under reduced pressure. The residue was purified by column chromatography (PE/EtOAc = 9:1) on silica gel to give the corresponding ketoxime **8** as colorless liquid (0.860 g, **94%**).

Note: Only (E)-oxime was observed. All data are in accordance with the literature.[1]

Major diastereomer (2R, 5R, E)

TLC: R_f = 0.8 (10% EtOAc in PE, revealed with PMA).

^{1}H NMR (360 MHz, $CDCl_3$): δ 4.74 (m, 2H), 3.83 (s, 1H), 3.36-3.29 (dd, *J* = 2.0, 3.8, 13.4 Hz, 1H), 2.25-2.01 (m, 2H), 2.00-1.91 (m, 1H), 1.90-1.81 (m, 1H), 1.74 (3H, s), 1.58 (dd, *J* = 12.6, 13.4 Hz, 1H), 1.42 (m, 1H), 1.26 (qd, *J* = 3.4, 12.6 Hz, 1H), 1.18 (d, *J* = 6.5 Hz, 3H) ppm.

^{13}C NMR (90 MHz, $CDCl_3$): δ 161.6, 148.7, 109.2, 61.0, 44.9, 37.2, 35.4, 30.9, 29.9, 20.8, 16.3 ppm.

[1] T. Kang, Y. Kim, D. Lee, Z. Wang, S. Chang, "Iridium-Catalyzed Intermolecular Amidation of sp3 C–H Bonds: Late-Stage Functionalization of an Unactivated Methyl Group" *J. Am. Chem. Soc.* **2014**, 136, 4141–4144.

Compound 9

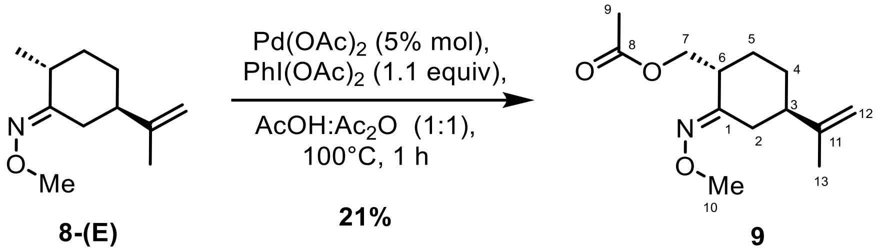

Oxime **8** (200 mg, 1.1 mmol, 1 equiv.), $PhI(OAc)_2$ (389 mg, 1.21 mmol, 1.1 equiv), and $Pd(OAc)_2$ (31.7 mg, 0.055 mmol, 5% mol.) were combined in AcOH (4 mL) and AcO_2 (6 mL) in a 20 mL vial. The vial was sealed with a Teflon lined cap, and the reaction was heated at 100 °C for 1 h 30. The resulting mixture was filtered through a plug of celite and diluted with pentane, and the pentane solution was washed with H_2O, saturated $NaHCO_3$ and brine. The organic layer was dried over $MgSO_4$, filtered, and concentrated to afford a colorless oil, which was purified by chromatography on silica gel (PE/EtOAc = 9:1). The acetate oxime **9** was obtained as a colorless oil (55 mg, **21%**).

Note: Diastereomers were not separated.

TLC: R_f = 0.5 (10% EtOAc in PE, revealed with PMA).
HRMS (ESI): *m/z* calculated for $C_{13}H_{21}NNaO_3$ $[M+Na]^+$ = 262.1413, found 262.1405.

Major diastereomer

^{1}H NMR (360 MHz, $CDCl_3$): δ 4.75 (m, 2H, H_{12}), 4.54 (dd, *J* = 4.8, 11.2 Hz, 1H, H_7), 4.05 (dd, *J* = 8.4, 11.2 Hz, 1H, H_7), 3.81 (s, 3H, H_{10}), 3.33 (m, 1H, H_6), 2.43 (m, 1H, H_3), 2.18-2.09 (m, 2H, H_2), 2.06 (s, 3H, H_9), 1.92 (m, 1H, H_5), 1.74 (s, 3H, H_{13}), 1.63-1.56 (m, 1H, H_5), 1.46 (qd, *J* = 3.5, 12.7 Hz, 1H, H_4), 1.27 (qd, *J* = 3.2, 12.5 Hz, 1H, H_4) ppm.

^{13}C NMR (90 MHz, $CDCl_3$): δ 171.3 (C_8), 157.9 (C_1), 148.5 (C_{11}), 109.6 (C_{12}), 64.9 (C_7), 61.4 (C_{10}), 44.8 (C_3), 41.4 (C_6), 30.2 (C_2), 30.2 (C_4), 29.6 (C_5), 21.0 (C_9), 20.8 (C_{13}) ppm.

Compound 12

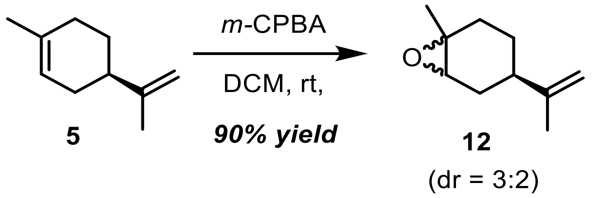

To a solution of (*R*)-limonene **5** (1.4 mmol, 200 mg) in DCM (10 mL) was added *m*-CPBA (1.5 mmol, 250 mg) at 0 °C. Then the mixture was stirred at room temperature for 2 h. The reaction mixture was treated with a saturated $NaHCO_3$ solution and the mixture was extracted with ether. The ether layer was washed with water and brine and dried over Na_2SO_4. Evaporation of the solvent gave a crude product. Purification by column chromatography (PE/EtOAc = 9:1) afforded the epoxide **12** mixture (3:2, 189 mg, **90%**).

Note: mixture of diastereomers (dr = 3:2). Diastereomeric ratio was evaluated by 1H NMR. All data are in accordance with the literature.[2]

Physical state: colorless oil.
TLC: R_f = 0.7 (5% EtOAc in PE, revealed with PMA).

<u>Major diastereomer</u>

^{1}H NMR (360 MHz, $CDCl_3$): δ 4.76 – 4.59 (m, 2H), 2.99 (d, *J* = 5.4, 1H), 2.20–1.97 (m, 2H), 1.93 – 1.79 (m, 1H), 1.76 – 1.61 (m, 5H), 1.48–1.12 (m, 5H) ppm.
^{13}C NMR (90 MHz, $CDCl_3$): δ 149.2, 109.2, 59.3, 57.5, 40.8, 30.9, 30.0, 24.4, 23.2, 20.3 ppm.

Compound 13

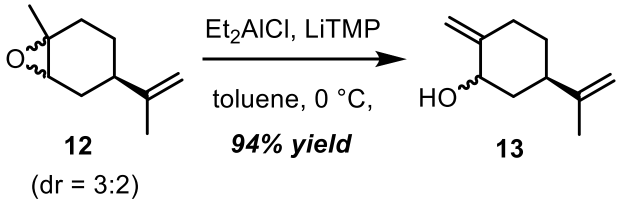

[2] O. Hauenstein, M. Reiter, S. Agarwal, B. Rieger, A. Greiner, "Bio-based polycarbonate from limonene oxide and CO2 with high molecular weight, excellent thermal resistance, hardness and transparency "*Green Chem.* **2016**, *18*, 760–770.

Freshly distilled TMP (4.64 mL, 26.2 mmol, 2 eq.) was diluted in toluene (20 mL), the resulting solution was cooled to 0°C, *n*-butyllithium (13.8 mL, 26.2 mmol, 1.9 M solution in hexanes) was then added, and the colorless solution turned yellow. After 30 min of stirring, diethylaluminum chloride (31.1 mL, 31.1 mmol, 1 M solution in hexanes, 2.4 eq.) was added, the yellow color disappeared, and a turbid solution formed. After a further 40 min of stirring at 0°C, the epoxide **12** (2.00 g, 13.1 mmol, 1 eq.) was added as a solution in toluene (7 mL). The reaction mixture was stirred for 45 min at 0 °C and then for 2 h at rt. The reaction was quenched with the slow addition of saturated $NaHCO_3$ at 0 °C, extracted with DCM, washed with brine, dried over $MgSO_4$, filtered, and concentrated under reduced pressure to give the crude product. Purification by column chromatography (PE/EtOAc = 4:1) afforded the alcohol product **13** as a 3:2 mixtures of diastereomers (1.88 g, **94%**).
Note: All data are in accordance with the literature.[3]

Physical state: colorless oil.
TLC: R_f = 0.2 (10% EtOAc in PE, revealed with PMA).

<u>Major diastereomer</u>

^{1}H NMR (360 MHz, $CDCl_3$): δ 4.95 (m, 1H), 4.79 (m, 1H), 4.77 (m, 1H), 4.71 (m, 2H), 4.15-4.06 (m, 1H), 2.46 (m, 1H), 2.26-2.13 (m, 2H), 2.11-1.95 (m, 2H), 1.89-1.78 (m, 2H), 1.73 (s, 3H), 1.33-1.18 (m, 2H) ppm.
^{13}C NMR (90 MHz, $CDCl_3$): δ 151.3, 149.9, 149.5, 148.7, 109.9, 109.2, 108.9, 103.9, 72.5, 72.2 ppm.

Compound 15

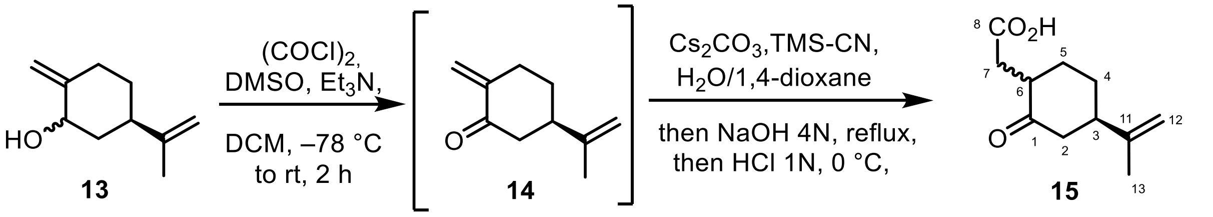

18% yield over 3 steps

DMSO (1.33 mL, 349 mmol, 6 equiv) in DCM (50 mL) was added dropwise to a solution of $(COCl)_2$ (1.13 mL, 13.1 mmol, 4 equiv) in DCM at –78 °C. 1 hour later, a solution of allylic alcohol **13** (0.5 g, 3.28 mmol) in DCM (10 mL) was added dropwise, followed by

[3] M. Uroos, W. Lewis, A. J. Blake, C. J. Hayes, "Total Synthesis of (+)-Cymbodiacetal: A Re-evaluation of the Biomimetic Route" *J. Org. Chem.* **2010**, *75*, 8465.

Et_3N (7.5 mL, 34.5 mmol, 10.5 equiv). The reaction mixture was kept at –78 °C for 10 min and then slowly warmed to room temperature. After completion, the reaction was quenched with an addition of saturated aqueous $NaHCO_3$. The layers were separated, and the aqueous layer was extracted with DCM and the organic layer was washed with NaCl. Then in the organic layer dioxane (10 ml) was added and only DCM was removed under reduced pressure.

Next, Cs_2CO_3 (214 mg, 0.66 mmol, 0.2 equiv) and water (350 µl) were added in the mixture. After stirring for 10 min at rt, TMSCN (1.63 ml, 13.2 mmol, 4 equiv) was added and the mixture was stirred for 24h at the same temperature. Then saturated aqueous $NaHCO_3$ and EtOAc were added. The layers were separated and washed with brine. The combined organic extracts were dried over anhydrous $MgSO_4$, and evaporated under reduced pressure. Then a solution of NaOH (15 mL, 4N) was added and the mixture was reflux for 5 h. After this time, 1N HCl was added until reaching pH 1 and stirred for 1 h. EtOAc was added and layers were separated and washed with brine. The combined organic extracts were dried over anhydrous $MgSO_4$, and evaporated under reduced pressure to give the crude product. Purification by column chromatography (DCM/MeOH = 9:1) afforded the acid product as mixture of diastereomers **15** (182 mg, 18%).

Physical state: white solid.
TLC: R_f = 0.8 (10% MeOH in DCM, revealed with PMA).
HRMS (ESI): *m/z* calculated for $C_{11}H_{16}NaO_3$ $[M+Na]^+$ = 219.0992, found 219.0991.

^{1}H NMR (360 MHz, $CDCl_3$): δ 4.80–4.76 (m, 2H, H_{12}), 2.86-2.80 (m, 2H, H_7-H_6), 2.52–2.48 (m, 1H, H_3), 2.38– 2.35 (m, 2H, H_2), 2.24-2.21 (m, 2H, H_4-H_7), 2.00–1.96 (m, 1H, H_5), 1.76 (s, 3H, H_{13}), 1.70 (m, 1H, H_5), 1.52-1.41 (qd, *J* = 3.47, 12.94, 1H, H_4) ppm.

^{13}C NMR (90 MHz, $CDCl_3$): δ 210.3 (C_1), 178.1 (C_8), 147.2 (C_{11}), 109.1 (C_{12}), 46.9 (C_2), 46.6 (C_6), 46.3 (C_3), 34.0 (C_7), 32.4 (C_4), 30.4 (C_5), 20.4 (C_{13}) ppm.

Compound 16: *all data were in accordance with the literature*.[3]

Compound 17

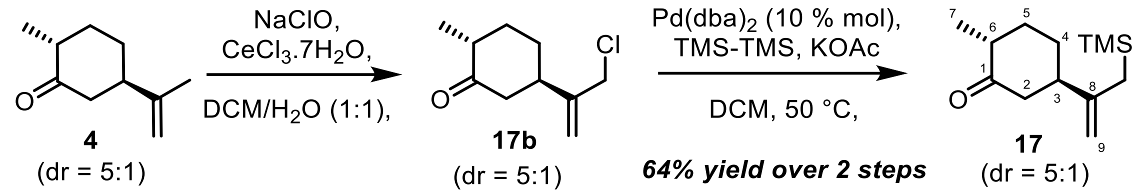

Dihydrocarvone **4** (500 mg, 3.2 mmol, 1 equiv), NaClO (9.8 mmol, 20% available chlorine, 3 mL, 3 equiv), $CeCl_3·7H_2O$ (9.8 mmol, 3.64 g, 3 equiv), DCM/H_2O (1:1, 30 mL) were used. After 30 min, saturated aqueous Na_2SO_3 is added and the mixture was extracted with DCM. The organic layer is dried over anhydrous sodium sulfate. Filtered, and concentrated under reduced pressure to give the crude product. Purification by column chromatography (PE/EtOAc, 95:5) afforded the corresponding chloride **17b** (98%, 604 mg) as a colorless oil.

*Note: Purification of **17b** was not necessary. Note: All data are in accordance with the literature*.[4]

TLC: R_f = 0.71 (10% EtOAc in PE).

^{1}H NMR (360 MHz, $CDCl_3$): δ 5.24 (m, 1H), 5.06 (m, 1H), 4.79 (m, 2H), 2.58-2.36 (m, 2H), 2.45-2.37 (m, 1H), 2.36-2.31 (m, 1H), 2.21-2.11 (m, 1H), 2.11-2.01 (m, 1H), 1.80-1.61 (m, 2H), 1.50-1.35 (qd, J = 3.4, 12.4 Hz, 1H), 1.07-1.05 (d, J = 6.5 Hz, 3H) ppm.
^{13}C NMR (90 MHz, $CDCl_3$): δ 211.6, 147.6, 116.4, 116.3, 46.9, 44.0, 34.6, 31.1, 30.4, 14.2 ppm.

$Pd(dba)_2$ (0.32 mmol, 186 mg, 10% mol), and KOAc (6.48 mmol, 634 mg, 2 equiv) were placed in a flask under argon. Then, DCM (6 mL), chloride **17b** (604 mg, 3.24 mmol, 1 equiv), and hexamethyldisilane (6.48 mmol, 1.26 mL, 2 equiv) were added and the mixture was carried out at 50 °C and the reaction mixture has turned pale yellow. After 24 h, the reaction mixture returned to red-brown solution. The mixture was quentch with water and extracted with EtOAc. The organic layer was dried over anhydrous sodium sulfate, filtered, and concentrated under reduced pressure to give the crude product. Purification by column chromatography (PE/EtOAc, 95:5) afforded the corresponding allylic silane **17** (68% over 2 steps, 501 mg) as a colorless oil.

Note: This reaction doesn't work with anhydrous KOAc. The reaction was performed with wet KOAc. Allylic acetate was formed in situ.

---

[4] D. S. Pisoni, D. Gamba, C. V. Fonseca, J. S. da Costa, C. L. Petzhold, E. R. de Oliveira, M. A. Ceschi, E. R. de Oliveira, M.A Ceschi, "InCl3/NaClO: a reagent for allylic chlorination of terminal olefins" *J. Braz. Chem. Soc.* **2006**, *17,* 321-327.

TLC: Rf = 0.82 (10% EtOAc in PE, revealed with PMA).
HRMS (ESI): *m/z* calculed for $C_{13}H_{24}NaOSi$ $[M+Na]^+$ = 247.1488, found 247.1479.

^{1}H NMR (360 MHz, $CDCl_3$): δ 4.63 (m, 2H, H_9), 2.55-2.43 (m, 1H, H_2), 2.41–2.29 (m, 1H, H_6), 2.28–2.16 (m, 1H, H_2), 2.15–2.03 (m, 2H, H_3-H_4), 2.02-1.92 (m, 1H, H_5), 1.64-1.52 (m, 1H, H_5), 1.51 (s, 2H, H_{10}), 1.38–1.21 (qd, J = 3.5, 13.0 Hz, 1H, H_4), 1.00 (d, J = 6.4 Hz, 3H, H_7), 0.02 (s, 9H, TMS) ppm.
^{13}C NMR (90 MHz, $CDCl_3$): δ 214.1 (C_1), 151.3 (C_8), 107.3 (C_9), 48.6 (C_2), 48.4 (C_6), 46.1 (C_3), 36.3 (C_5), 32.6 (C_4), 26.9 (C_{10}), 15.6 (C_7), -0.0 (TMS) ppm.

Compound 19

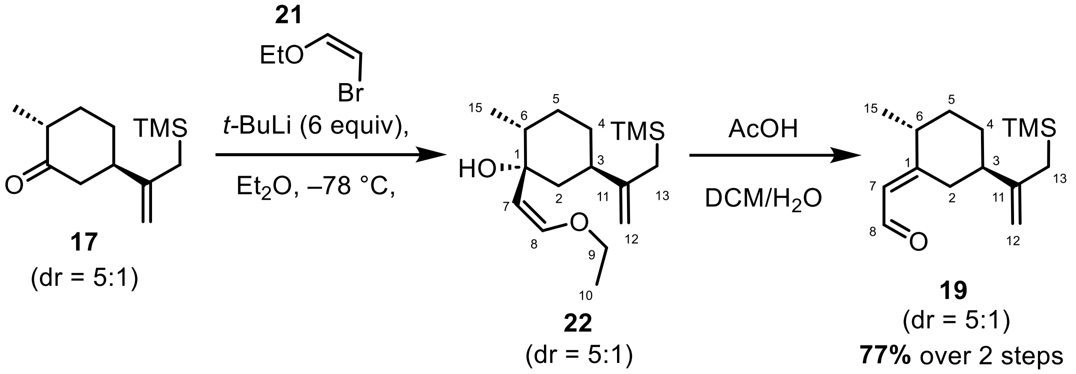

(*Z*)-1-bromo-2-ethoxyethylene **21** (0.165 mL, 1.33 mmol, 3 equiv) was added to an oven-dried flask containing diethyl ether (5 mL). The solution was cooled to –78 °C, and *t*-BuLi (1.6 mL, 2.6 mmol, 6 equiv, 1.7 M in pentane) was added, dropwise. The reaction was stirred, at –78 °C, for 30 min. A solution of ketone **17** (0.100 g, 0.44 mmol dissolved in 2.2 mL Et_2O) was added dropwise, via syringe, and the reaction was stirred for 2.5 h at –78 °C. The reaction was quenched with saturated $NaHCO_3$ and allowed to warm to rt. The separated aqueous phase was extracted with EtOAc and the combined organic phases were washed with brine. The organic layer was dried over Na_2SO_4, filtered, and concentrated under reduced pressure. Purification by silica gel chromatography (PE/ diethyl ether = 95:5) afforded the ethoxyvinyl **22** as a colorless oil.

*Note: **21** was prepared according literature procedure.*[5]

[5] C. Henry, D. Bolien, B. Ibanescu, S. Bloodworth, D.C. Harrowven, X. Zhang, A. Craven, H. F. Sneddon, R.J. Whitby, "Generation and Trapping of Ketenes in Flow" *Eur. J. Org. Chem*. **2015**, *7,* 1491-1499.

TLC: R_f = 0.78 (10% EtOAc in PE, revealed with PMA)
HRMS (ESI): *m/z* calculed for $C_{17}H_{32}NaO_2Si$ $[M+Na]^+$ = 319.2063, found 319.2046.

^{1}H NMR (360 MHz, $CDCl_3$): δ 5.95 (d, *J* = 7.1 Hz, 1H, H_8), 4.52 (m, 2H, H_{12}), 4.32 (d, *J* = 7.1 Hz, 1H, H_7), 3.83-3.73 (m, 2H, H_9), 2.22-2.09 (tt, *J* = 2.9, 15.2 Hz, 1H, H_3), 1.98-1.92 (dt, *J* = 2.9, 13.2. Hz, 1H, H_2), 1.80-1.68 (m, 1H, H_4), 1.53 (s, 2H, H_{13}), 1.50-1.40 (m, 2H, H_5), 1.31 (m, 1H, H_3), 1.24 (t, *J* = 7.3 Hz, 3H, H_{10}), 1.22 (m, 1H, H_2) 1.11 (m, 1H, H_4), 0.90 (d, *J* = 6.9 Hz, 3H, H_{15}), 0.0 (s, 9H, TMS) ppm.
^{13}C NMR (90 MHz, $CDCl_3$): δ 154.1 (C_{11}), 145.8 (C_8), 116.2 (C_7), 106.0 (C_{12}), 76.1 (C_9), 69.6 (C_1), 46.7 (C_2), 41.5 (C_6), 40.7 (C_3), 33.3 (C_4), 31.2 (C_5), 27.6 (C_{13}), 16.9 (C_{15}), 16.4 (C_{10}), 0.0 (TMS) ppm.
The ethoxyvinyl **22** was diluted in solution of DCM/water (1:2, 10 ml) and 3 ml of acetic acid was added. Then the mixture was stirred for 24 h at room temperature. The separated aqueous phase was extracted with DCM and the combined organic phases were washed with brine. The organic layer was dried over Na_2SO_4, filtered, and concentrated under reduced pressure. Purification by silica gel chromatography (PE/ diethyl ether 95:5) afforded the enal product **19** as a colorless oil (0.085 g, 77% over 2 steps).
Note: Only E stereoisomer was obtained and only one conformer was present. The enal product was diluted in Et_2O and put in a fridge to avoid degradation.

TLC: R_f = 0.8 (10% EtOAc in in PE, revealed with PMA, UV).
HRMS (ESI): *m/z* calculed for $C_{15}H_{26}NaOSi$ $[M+Na]^+$ = 273.1645, found 273.1641.

Major diastereomer

^{1}H NMR (360 MHz, $CDCl_3$): δ 10.09 (d, *J* = 8.2 Hz, 1H, H_8), 5.85 (d, *J* = 8.2 Hz, 1H, H_7), 4.66 (m, 2H, H_{12}), 3.51 (m, 1H, H_2), 2.27 (m, 1H, H_6), 2.09-1.90 (m, 4H, H_3-H_2-H_4-H_5), 1.59 (s, 2H, H_{13}), 1.47 (m, 1H, H_5), 1.24 (m, 2H, H_4), 1.10 (d, *J* = 6.6 Hz, 3H, H_{15}), 0.03 (s, 9H, H_{14}) ppm.
^{13}C NMR (90 MHz, $CDCl_3$): δ 190.8 (C_8), 171.0 (C_1), 150.8 (C_{11}), 122.6 (C_7), 105.8 (C_{12}), 47.9 (C_3), 39.7 (C_6), 36.7 (C_4), 35.6 (C_2), 32.2 (C_5), 26.2 (C_{13}), 17.5 (C_{15}), -1.3 (TMS) ppm.

Compound 19 – *Via Nagata homologation*

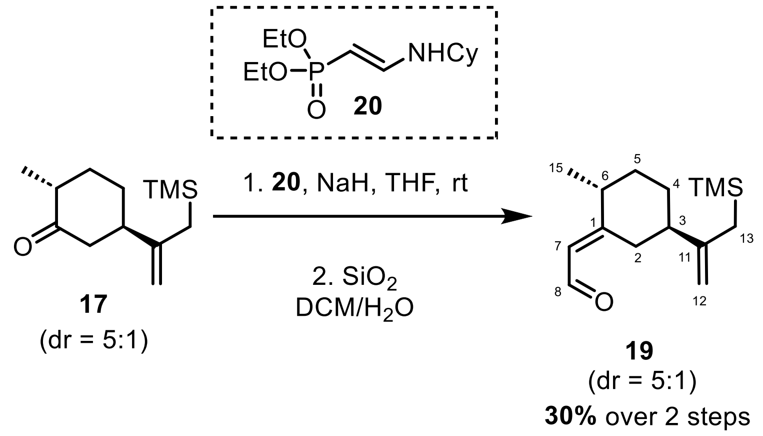

To a suspension of sodium hydride (60 % in oil, 1.5 equiv, 1.11 mmol) in anhydrous THF (5 mL) was added a solution of a phosphonate aldimine **20** (1.7 equiv, 1.25 mmol) in THF (2 mL) with stirring and ice-cooling in an argon atmosphere, and the mixture was stirred for 15 min. A solution of a ketone **17** (167 mg, 0.74 mmol, 1 equiv) in THF (5 mL) was then added, and the mixture was stirred at room temperature. After 1.5 h, the reaction mixture was poured into ice-water and extracted with ether. The extracts were washed with saturated sodium chloride solution, dried, and evaporated concentrated under reduced pressure to give the crude aldimine.

The aldimine mixture was then diluted in mixture H_2O/DCM (5:2, 10 mL), 10% of silica was added and the mixture was stirred for 24 h at room temperature. The separated aqueous phase was extracted with DCM and the combined organic phases were washed with brine. The organic layer was dried over Na_2SO_4, filtered, and concentrated under reduced pressure Purification by silica gel chromatography (5%-20% EtOAc in PE) afforded the enal product **19** as a colorless oil (55 mg, 30%).

*Note: Aldimine **20** was prepared according literature procedure.*[6] *Only diastereomer E was obtained and only one conformer was present.*

Compound 19 – *Via Babler-Dauben oxidative rearrangement*

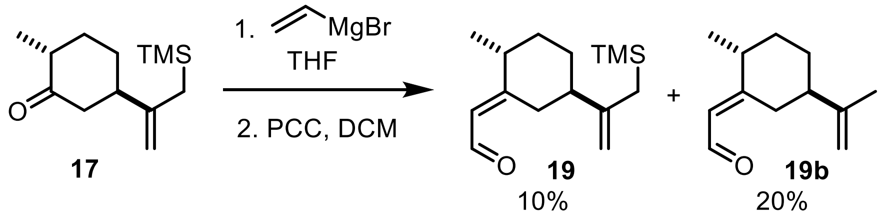

[6] W. Nagata, Y. Hayase, "Formylolefination of carbonyl compounds" *J. Chem. Soc. C*, **1969**, *3*, 460-466.

Vinyl magnesium bromide (1.0 M in THF, 0.9 mL, 0.9 mmol, 2 eq.) was added to a solution of ketone **17** (98 mg, 0.44 mmol) in THF (2 mL) at 0°C and the mixture was stirred for 5 hours at room temperature. The reaction was quenched with saturated solution of NH_4Cl. The phases were separated and the aqueous phase was extracted with Et_2O. The organic phases were combined, dried over $MgSO_4$, and solvents were evaporated under reduced pressure to afford the crude alcohol **18** which was directely involved in the following step. To a solution of the crude allylic alcohol **18** in DCM (10 mL) was added PCC (567 mg, 2.64 mmol) in two portions. After stirring for 12 h, the reaction was quenched with a 2.0 M aqueous solution of NaOH and extracted with DCM. The organic layer was washed with brine, dried over anhydrous Na_2SO_4 and concentrated under reduced pressure. The residue was purified by column chromatography (silica gel, PE /EtOAc, 9:1) to afford enal **19**-(*E*) as a colorless oil (11 mg, 10%) along with protodesilylation product **19b** (16 mg, 20%).
All date were in accordance with the literature.[7]

Compound 23

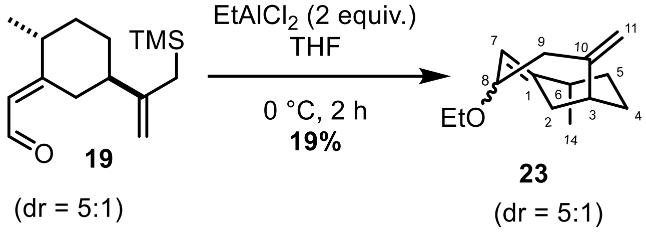

To a stirred solution of the enal TMS **19** in toluene at 0 °C under argon was added a solution of ethylaluminum dichloride in toluene (2.0 M, 2 equiv.). The resulting red solution was stirred at 0 °C for 2 h. After complete conversion, the mixture was diluted in ether and quenched with satured solution of $NaHCO_3$. The organic layer was separated, dried and evaporated. The crude material was purified by gel silica chromatography (5%-20% EtOAc in PE) afforded the product **23** as a colorless oil (4 mg, **19%**).
Note: Degradation was observed during purification. Spectral data for the Major diastereomer is reported.

TLC: Rf = 0.8 (10% EtOAc in PE, revealed with PMA).
HRMS (ESI): *m/z* calculated $C_{14}H_{22}O_1$ $[M\text{-}OEt]^+$= 161.1319, found 161.1324.

[7] T. Gerke, C. Kropf, U. Huchel, A. Griesbeck, B. Porschen (Henkel AG), US 2018/0170849 A1, **2018**.

Major diastereomer

^{1}H NMR (360 MHz, $CDCl_3$): δ 5.48 (d, *J* = 5.1 Hz, 1H, H_7), 4.72 (m, 2H, H_{11}), 4.31 (m, 1H, H_8), 3.62 (m, 1H, H_{12}), 3.48 (m, 1H, H_{12}), 2.68 (m, 1H, H_3), 2.53-2.27 (m, 3H, H_9-H_6), 2.16 (m, 1H, H_2), 1.94 (m, 1H, H_2), 1.81-1.71 (m, 2H, H_5), 1.53-1.47 (m, 1H, H_4), 1.30-1.21 (m, 4H, H_{13}-H_4), 1.06 (d, *J* = 6.7 Hz, 3H, H_{14}) ppm.
^{13}C NMR (90 MHz, $CDCl_3$): δ 150.9 (C_{10}), 142.3 (C_1), 129.5 (C_7), 111.9 (C_{11}), 77.2 (C_8), 63.9 (C_{12}), 41.6 (C_6), 39.9 (C_9), 39.4 (C_3), 31.5 (C_4 or C_5), 27.7 (C_2), 25.0 (C_4 or C_5), 18.1 (C_{14}), 15.4 (C_{13}) ppm.

***Procedure A**: Knoevenagel olefination*

A mixture of ketone, methyl cyanoacetate **26** (1.1 equiv), ammonium acetate (1-5 equiv), glacial acetic acid (4 equiv), and toluene or DCM was heated at reflux until complete conversion. The reaction mixture was cooled to room temperature and washed successively with water, $NaHCO_3$ solution, and brine. Drying, filtration, and evaporation of the organic phase provided crude oil that was purified via through gel silica chromatography.

Compound 27

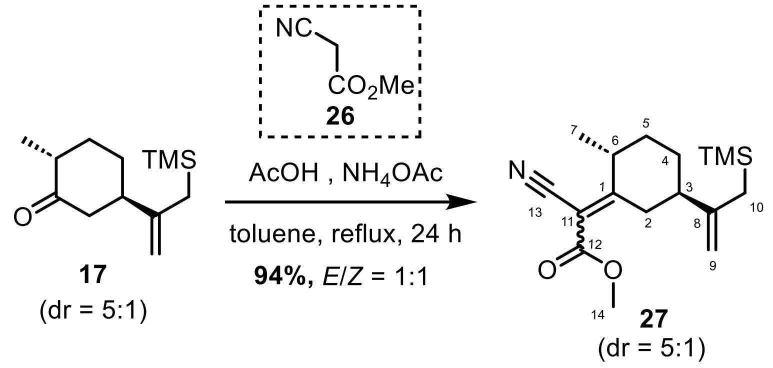

Prepared according to general procedure A. To a solution of TMS dihydrocarvone **17** (100 mg, 0.44 mmol), in 2 ml of toluene, was added methyl cyanoacetate **26** (0.055 ml, 0.49 mmol, 1.1 equiv), ammonium acetate (33.8 mg, 0.44 mmol, 1 equiv) and 0.10 ml (1.76 mmol, 4 equiv) of glacial acetic acid. The mixture was stirred at reflux with Dean-Stark for 24 h. Purification by silica gel chromatography (5%-20% EtOAc in PE) afforded the Knoevenagel product **27** as a colorless oil (127 mg, 94%).
Note: mixture of diastereomers E/Z (1:1) which are not separable.

TLC: R_f = 0.6 (10% EtOAc in PE, revealed with PMA, UV).
HRMS (ESI): *m/z* calculed for $C_{17}H_{27}NNaO_2Si$ $[M+Na]^+$ = 328.1687, found 328.1703.

Major diastereomer

^{1}H NMR (360 MHz, $CDCl_3$) : δ 4.77-4.62 (m, 2H, H_9), 4.13 (m, 1H, H_6, *Z*), 3.93 (m, 1H, H_2, *E*), 3.82 (s, 3H, H_{14}), 3.33 (m, 1H, H_6, *E*), 2.98 (m, 1H, H_2, *Z*), 2.43 (m, 1H, H_2, *Z*), 2.12 (m, 1H, H_2, *E*), 1.90-1.59 (m, 5H, H_3-H_4-H_5), 1.56 (s, 2H, H_{10}), 1.24 (d, *J* = 7.2 Hz, 3H, TMS, *E*), 1.20 (d, *J* = 7.2 Hz, 3H, TMS, *Z*) ppm.
^{13}C NMR (90 MHz, $CDCl_3$) : δ 183.5 (C_1), 162.4 (C_{12}), 150.4 (C_8), 115.3 (C_{13}), 106.1 (C_9), 102.6 (C_{11}), 52.5 (C_{14}), 47.2 (C_3), 37.8 (C_2, *Z*), 37.3 (C_6, *E*), 32.8 (C_5-C_4), 32.01 (C_2, *E*), 31.8 (C_6, *Z*), 26.0 (C_{10}), 25.9 (C_5-C_4), 18.6 (C_7), -1.3 (TMS) ppm.

Compound 25

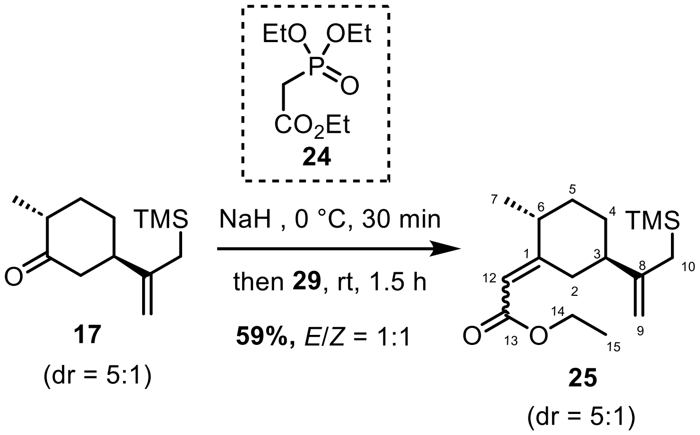

A suspension of sodium hydride (60% in oil, 1.5 equiv, 2.5 mmol, 100 mg) in hexanes under argon atmosphere was magnetically stirred for 10 min and the solvent was syringed out. The oil free sodium hydride was suspended in dry THF (10 mL) and cooled in an ice bath. Triethyl phosphonoacetate **24** (1.7 equiv, 2.8 mmol, 627 mg) in dry THF (2 mL) was added dropwise and the reaction mixture was stirred for 0.5 h at room temperature. The reaction mixture was cooled to 0 °C and a solution of trimethyl silyl ketone **17** (384 mg, 1.71 mmol) in dry THF (5 mL) was added dropwise. The mixture was stirred for 16h and was quenched by careful addition of saturated aqueous ammonium chloride solution and diluted with ether. The ether layer was separated and the aqueous layer was extracted with ether. The combined ether extract was washed with brine and dried (Na_2SO_4). Solvent was evaporated and the residue was purified

by silica gel chromatography (5%-20% EtOAc in PE) afforded the HWE silyl product **25** as a colorless oil (299 mg, 59%).

Note: mixture of diastereomers Z/E (1:1) which are not separable, no conformer was observed.

TLC: R_f = 0.6 (10% EtOAc in PE, revealed with PMA, UV).
HRMS (ESI): *m/z* calculed for $C_{17}H_{30}NaO_2Si$ $[M+Na]^+$ = 317.1907, found 317.1902.

^{1}H NMR (360 MHz, $CDCl_3$) : δ 5.68 (m, 1H, $H_{12,}$ Z), 5.58 (m, 1H, $H_{12,}$ E), 4.68-4.64 (dt, *J* = 1.2, 12.5 Hz, 1H, H_9), 4.57 (m, 1H, H_9), 4.14 (q, *J* = 7.1 Hz, 2H, H_{14}), 4.05 (m, 1H, H_6 , *Z*), 3.71 (m, 1H, $H_{6,}$ *E*), 2.47 (m, 1H, $H_{2,}$ *E*), 2.30 (m, 1H, $H_{2,}$ *Z*), 2.14 (m, 1H, $H_{2,}$ *Z*), 2.08 (m, 1H, $H_{2,}$ *E*), 1.82 (m, 1H, H_3), 1.72-1.51 (m, 6H, $H_{10\text{-}5\text{-}4}$), 1.26 (t, *J* = 7.1 Hz, 3H, H_{15}), 1.13 (d, *J* = 7.0 Hz, 3H, H_7), 0.01 (s, 3H,TMS) ppm.
^{13}C NMR (90 MHz, $CDCl_3$) : δ 168.1 (C_{13}), 167.7 (C_1), 152.7 (C_8), 114.7 (C_{12}), 106.8 (C_9), 60.9 (C_{14}), 48.8 (C_3), 40.8 ($C_{6,}$ *E*), 39.9 ($C_{2,}$ *Z*), 34.6 (C_4-C_5), 31.9 ($C_{2,}$ *E*), 31.5 ($C_{6,}$ *Z*), 27.7 (C_4-C_5) 27.3 (C_{10}), 20.2 (C_7), 15.6 (C_{15}), 0.03 (TMS) ppm.

Compound 29

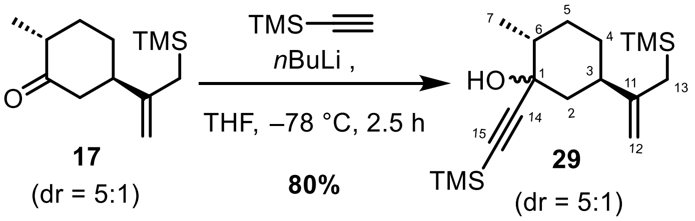

To a THF solution (8 mL) of trimethylsilylacetylene (0.5 mL, 3.5 mmol, 4 equiv) was added *n*-BuLi (1.8 mL, 2.6 mmol, 2.2 M in hexane, 3 equiv) dropwise at –78 °C under an argon atmosphere. The excess dry ice was removed and the reaction was allowed to slowly warm to 0 °C over 1.5 h, then recooled to –78 °C. Next, **17** (0.200 g, 0.89 mmol, 1 equiv) was added dropwise as a solution in THF (2.0 mL). The reaction was stirred for 1.5 h and allowed to slowly warm to room temperature. Saturated aqueous NH_4Cl solution was added to quench the reaction and the mixture was extracted with diethyl ether. The organic layer was washed with water, saturated aqueous sodium bicarbonate, and brine. The organic layer was dried over $MgSO_4$, filtered, and concentrated. The crude product was purified by silica gel chromatography (10-20% EtOAc in PE) to give **29** as a colorless oil (230 mg, 80%).

Note: Note: Diastereomeric ratio at C1 was not determined due to the peaks of each diastereomer overlapping.

.

TLC: R_f = 0.51 (20% EtOAc in PE, revealed with PMA).
HRMS (ESI): *m/z* calculed for $C_{18}H_{34}NaOSi_2$ $[M+Na]^+$ = 345.2047, found 345.2027.

Major diastereomer

^{1}H NMR (360 MHz, $CDCl_3$): δ 4.59 (m, 2H, H_{12}), 2.26 (s, 1H, OH), 2.12 (m, 2H, H_2), 1.83 (m, 1H, H_4 or H_5), 1.72 (m, 1H, H_3), 1.67 (m, 1H, H_4 or H_5), 1.59 (s, 2H, H_{13}),1.53 (m, 1H, H_6), 1.43 (m, 1H, H_2), 1.31 (m, 1H, H_4 or H_5), 1.12 (m, 1H, H_4 or H_5), 1.07 (d, *J* = 6.4 Hz, 3H, H_7), 0.19 (s, 9H, TMS), 0.05 (s, 9H, TMS) ppm.
^{13}C NMR (90 MHz, $CDCl_3$): δ 151.2 (C_{11}), 106.6 (C_{14}), 105.1 (C_{12}), 90.9 (C_{15}), 73.7 (C_1), 46.1 (C_2), 42.7 (C_3), 42.5 (C_6), 32.3 (C_4 or C_5), 31.8 (C_4 or C_5), 26.6 (C_{13}), 15.7 (C_7), 0.01 (TMS), -1.16 (TMS) ppm.

Compound 28

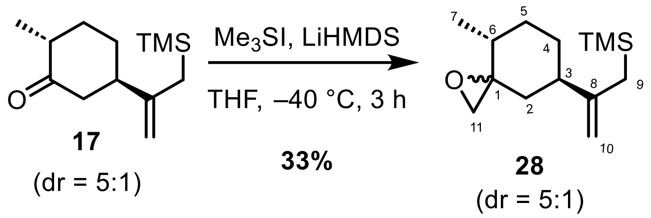

17 (dr = 5:1) **28** (dr = 5:1)

LiHMDS (1 M in hexanes, 0.89 mL, 8.9 mmol, 2 equiv) was added dropwise to a stirred solution of trimethylsulfonium iodide (0.2 g, 0.98 mmol, 2.2 equiv) in dry THF (10 mL) under argon at –40 °C. After 5 min, TMS-methylcyclohexenone **17** (100 mg, 0.44 mmol, 1 equiv) was added dropwise and the mixture was stirred at –40 °C for 30 min. The reaction mixture was allowed to reach room temperature and concentrated under reduced pressure. Water was added, and the mixture was extracted with n-pentane. The organic phases were washed with water and brine, dried over $MgSO_4$, and concentrated under reduced pressure. The crude product was purified by column chromatography on silica gel (5% diethyl ether in hexanes) affording **28** as a colorless oil (35 mg, 33%).

Note: Diastereomeric ratio at C1 was not determined due to the peaks of each diastereomer overlapping.

TLC: R_f = 0.72 (10% EtOAc in PE, revealed with PMA).
HRMS (ESI): *no stable.*

Major diastereomer

^{1}H NMR (360 MHz, $CDCl_3$): δ 4.57 (m, 2H, H_{10}), 2.83 (m, 1H, H_{11}), 2.45 (m, 1H, H_{11}), 2.09 (m, 1H, H_3), 1.93-1.69 (m, 4H, H_2-H_4-H_5-H_6), 1.54 (m, 2H, H_9), 1.42-1.26 (m, 3H, H_2-H_4-H_5), 0.73 (d, J = 6.5 Hz, 3H, H_7), 0.03 (m, 9H, TMS) ppm.
^{13}C NMR (90 MHz, $CDCl_3$): δ 151.1 (C_8), 105.1 (C_{10}), 60.4 (C_1), 50.8 (C_{11}), 42.9 (C_3), 40.5 (C_2), 33.5 (C_6), 32.9 (C_4 or C_5), 31.9 (C_4 or C_5), 26.0 (C_9), 14.0 (C_7), -1.2 (TMS) ppm.

Compound 38

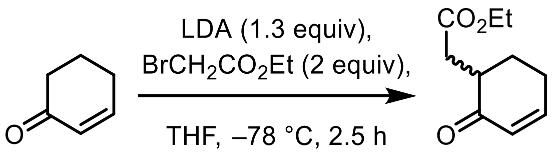

33 **38** **70%**

To a solution of dist. diisopropylamine (6.76 g, 67.6 mmol, 1.3 equiv) in 50 mL of THF at -78 °C was added *n*-butyllithium (37 mL, 67.6 mmol, 1.8 M, 1.3 equiv) in hexane, and the solution was stirred for 15 min. To the solution of lithium diisopropylamine (LDA) was added 2-cyclohexen-1-one **33** (5.0 g, 52.0 mmol) in 15 mL of THF at a rate that allowed a temperature at or below –50 °C to be maintained. The solution was stirred for 10 min at –78 °C followed by the addition of a solution of ethyl bromoacetate (17.3 g, 104 mmol, 2 equiv) in 15 mL of THF at a rate that allowed a temperature at or below -50 °C to be maintained. The solution was stirred for 2.5 h at –78 °C. The solution was diluted with ether and quenched with saturated aqueous NH_4Cl. The mixture was allowed to warm to approximately 0 °C, and the aqueous layer was extracted with ether. The combined organic extracts were washed with saturated aqueous NH_4Cl and saturated NaCl, dried over anhydrous $MgSO_4$, and evaporated under reduced pressure. The residue was chromatographied on a silica gel column with EtOAc/PE (1:9 to 2:8) to give ester **38** as a colorless oil (6.62 g, 70%).

Note: The reaction was also performed on 40 g scale with a loss of 10% yield. All data are in accordance with the literature.[8]

TLC: R_f = 0.4 (20% EtOAc in PE, revealed with PMA, UV).

^{1}H NMR (360 MHz, $CDCl_3$): δ 6.98–6.90 (m, 1H), 5.99 (d, *J* = 10.2 Hz, 1H), 4.13 (qd, *J* = 7.1, 1.9 Hz, 2H), 2.90–2.80 (m, 2H), 2.47– 2.35 (m, 2H), 2.24 (td, *J* = 8.7, 2.2 Hz, 1H), 2.13–2.06 (m, 1H), 1.80 (m, 1H), 1.24 (t, *J* = 7.1 Hz, 3H) ppm.

^{13}C NMR (90 MHz, $CDCl_3$): δ 199.3, 172.3, 150.1, 129.1, 60.4, 43.6, 34.5, 28.6, 25.9, 14.2 ppm.

Compound 32

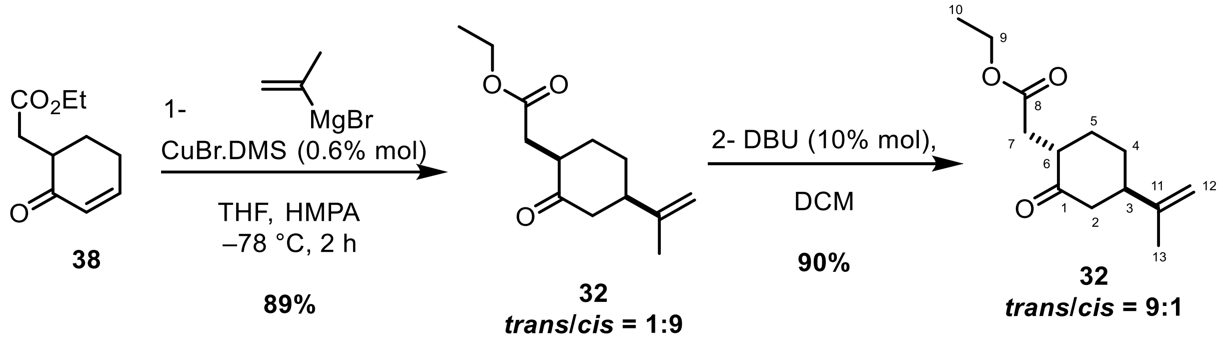

A flame-dried 250 mL round-bottom flask under argon was charged with CuBr·SMe_2 (33 mg, 0.162 mmol, 0.6% mol) and dist. THF (50 mL), then stirred vigorously for 1 h. A flame-dried 150 mL three-neck flask under argon, equipped with an addition funnel, was charged Mg turnings (4.53 g, 189 mmol, 7.0 equiv). The flask was then flame-dried under reduced pressure and back-filled with argon, then dist. THF (4 mL) was added, followed by bromopropene (0.1 equiv) and 1 ball of I_2. The brown reaction mixture was heated at 55 °C until decoloration was observed (30 min). The hot plate was removed and bromopropene (6.04 mL, 67 mmol, 2.5 equiv), in solution of dist. THF (50 mL) was added dropwise over 1 h. The rate of addition and the mixture's temperature was controlled to maintain gentle reflux. The dark-grey solution of Grignard reagent was allowed to cool to room temperature, stirred for 30 min, and titrate with I_2 method.

[8] R. L. Bassfield, K. F. Podraza, "Regiospecific Alkylation of 3-Substituted-2-cyclohexen-l-ones. Synthesis and Conformational Analysis of 6-(Carbethoxymethyl)-3-substituted-2-cyclohexen-l-ones" *J. Org. Chem.* **1989**, *54*, 5919–5922.

The flask containing the $CuBr·SMe_2$ solution was cooled to -78 °C, then a solution of HMPA (30 mL, 6 equiv), ester cyclohexenone **38** (5 g, 27 mmol, 1 equiv) and dist. THF (50 mL) was added dropwise at -78°C. The reaction mixture was then stirred for 20 min at -78 °C and then the Grignard (0.5 mol/L) solution was added dropwise via cannula over 1h. The reaction mixture was then stirred for 2 h at -78 °C and quenched by sat. aq. NH_4Cl. The layers were separated and the aqueous phase was extracted with EtOAc. Combined organic layers were dried over Na_2SO_4 and concentrated under a reduced pressure. The crude product was purified by silica gel chromatography (20% EtOAc in PE) to give a colorless oil of **32** (5.37 g, 89%) as a mixture of diastereomer 1:9 (*trans*/*cis*).

The purified ester was diluted in DCM (50 mL) and DBU (381 mL, 0.1 equiv) was added. The mixture was stirred overnight and sat. aq. NH_4Cl was added. The layers were separated and the aqueous phase was extracted with EtOAc. Combined organic layers were dried over Na_2SO_4 and concentrated under a reduced pressure. The crude product was purified by silica gel chromatography (20% EtOAc in PE) to give a colorless oil of **32** (4.89 g, 90%) as a mixture of diastereomers 9:1 (*trans*/*cis*).

Note: Diastereomers (dr = 9:1 (trans/cis)) can be separated. Diastereomeric ratio was evaluated by ^1H NMR.

TLC: R_f = 0.6 (20% EtOAc in PE, revealed with PMA)
HRMS (ESI): *m/z* calculated for $C_{13}H_{20}NaO_3$ $[M+Na]^+$ = 247.1304, found 247.1305.

^{1}H NMR (360 MHz, $CDCl_3$): δ 4.78–4.75 (m, 2H, H_{12}), 4.18-4.12 (qd, *J* = 7.1, 1.6 Hz, 2H, H_9), 2.91-2.75 (m, 2H, H_6-H_7), 2.50–2.48 (m, 1H, H_3), 2.38– 2.35 (m, 2H, H_2), 2.21-2.15 (m, 2H, H_7-H_4), 2.00–1.96 (m, 1H, H_5), 1.76 (s, 3H, H_{13}), 1.70 (m, 1H, H_5), 1.45 (qd, *J* = 3.4, 12.9 Hz, 1H, H_4), 1.27 (t, *J* = 7.1 Hz, H_{10}) ppm.
^{13}C NMR (90 MHz, $CDCl_3$): δ 210.3 (C_1), 172.5 (C_8), 147.3 (C_{11}), 109.8 (C_{12}), 60.4 (C_9), 46.9 (C_2), 46.6 (C_6), 46.4 (C_3), 34.2 (C_7), 32.4 (C_4), 30.5 (C_5), 20.4 (C_{13}), 14.2 (C_{10}) ppm.

Compound 34

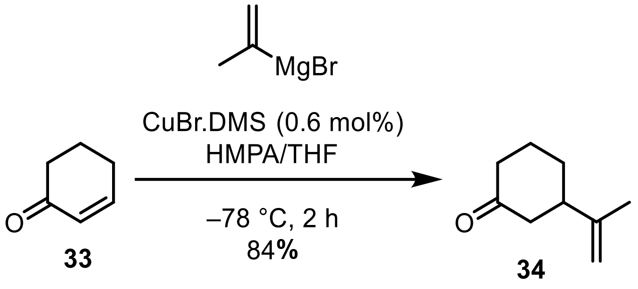

To a mixture of CuBr·SMe_2 (2.53 g, 12.3 mmol) in THF (31 mL) was added dropwise a 0.5 M solution of isopropenylmagnesium bromide in THF (49.3 mL, 24.6 mmol) at –50 °C over 15 min, and the mixture was stirred at the same temperature for 20 min. Then, a solution of freshly distilled 2-cyclohexen-1-one **33** (1.08 mL, 11.2 mmol), TMSCl (2.84 mL, 22.4 mmol), and HMPA (3.90 mL, 22.4 mmol) in THF (14 mL) was added dropwise at –78 °C, and stirring was continued at –78 °C for 3.5 h. The reaction mixture was treated with 1M HCl and the resulting mixture was stirred vigorously at room temperature for 1 h. After the layers were separated, the aqueous layer was extracted with Et_2O. The combined organic layers were washed with wate and brine, dried over $MgSO_4$, and concentrated under reduced pressure. The crude product was purified by silica gel chromatography (20% EtOAc in PE) to afford the ketone **34** (1.98 g, **84%**) as a colorless oil.
Note: All data are in accordance with the literature.[9]

TLC: R_f = 0.6 (20% EtOAc in PE).
^{1}H NMR (360 MHz, $CDCl_3$): δ 4.77 (s, 1H), 4.72 (s, 1H), 2.48-2.15 (m, 5H), 2.15-1.99 (m, 1H), 1.99-1.84 (m, 1H), 1.73 (s, 3H), 1.71-1.49 (m, 2H) ppm.
^{13}C NMR (90 MHz, $CDCl_3$): δ 211.8, 147.7, 110.3, 46.9, 45.9, 41.5, 30.2, 25.4, 20.8 ppm.

[9] J. Küppers, P. Becker; R. Jarling, M. Dörries, N. Cakić, M. Schmidtmann, J. Christoffers, R. Rabus, H. Wilkes, "Stereochemical Insights into the Anaerobic Degradation of 4-Isopropylbenzoyl-CoA in the Denitrifying Bacterium Strain pCyN1" *Chem. Eur. J.* **2019**, *25*, 4722.

Compound 32

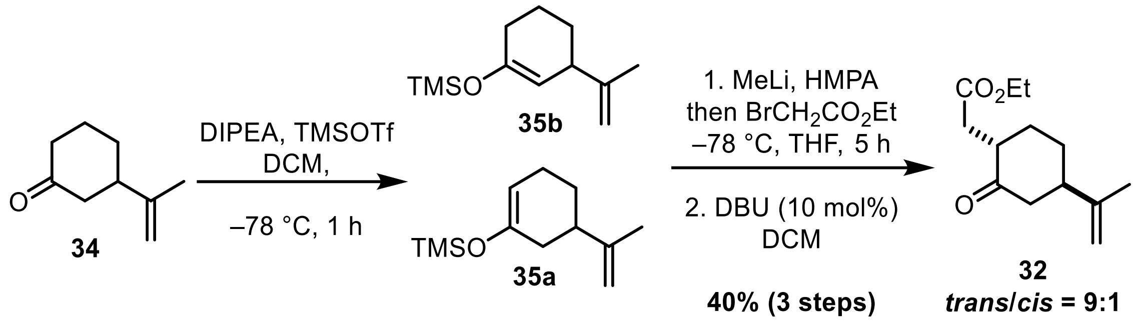

A solution of ketone **34** (0.200 g, 1.5 mmol) and $^{i}Pr_2NEt$ (freshly distilled over CaH_2, 0.87 mL, 5.0 mmol, 3.5 equiv) in DCM (5 mL) was cooled to –78 ºC. A solution of TMSOTf (freshly distilled, 0.81 mL, 4.5 mmol, 3 eq.) in DCM (0.5 mL) was added dropwise over 10 min. After completion as observed by TLC (~1 h), the reaction was quenched at –78 ºC by addition of sat. aq. $NaHCO_3$. The mixture was diluted with pentane or hexane and H_2O, the layers were separated, and the aqueous layer was extracted with pentane or hexanes. The combined organic extracts were washed with brine, dried over anhydrous $MgSO_4$, filtered and concentrated under reduced pressure. The crude product was engaged without purification (mixture of regiomers 4:1 (**35a**/**35b**).

To a solution of enol ether (0.275 g, 1.5 mmol) and HMPA (1.3 mL, 7.5 mmol, 5 equiv) in THF (1.5 mL) was added dropwise MeLi (1.6 M in hexane, 1.8 mL, 3.0 mmol, 2 equiv) at 0 °C and stirring was continued at the same temperature for 10 min. Ethyl bromoacetate (0.58 mL, 5.2 mmol, 3.5 equiv.) was added at –78 °C and the mixture was stirred at –78 °C for 3.5 h, and then –60 °C for 1.5 h. The reaction was quenched with saturated aqueous NH_4Cl solution. After the layers were separated, the aqueous layer was extracted with Et_2O. The combined organic layers were washed with water, and brine, dried over $MgSO_4$, and concentrated. The residue was purified by silica chromatography (9:1 = PE/EtOAc) to afford ester **32** as a mixture of diastereomers 1:9 (*trans*:*cis*).

The purified ester (153 mg, 0.61 mmol 1 equiv) was diluted in DCM (10 mL) and DBU (0.178 mL, 1.2 mmol 2 equiv) was added. The mixture was stirred overnight. Then sat. aq. NH_4Cl was added, the layers were separated and the aqueous phase was extracted with EtOAc. Combined organic layers were dried over Na_2SO_4 and

concentrated under reduced pressure to give colorless oil of ester **32** (129 mg, 40% overs 3 steps) as a mixture of diastereomers 9:1 (*trans*:*cis*).

Note: For analytic data, see previous page. Only the good regiomer was recovered after purification.

Compound 36

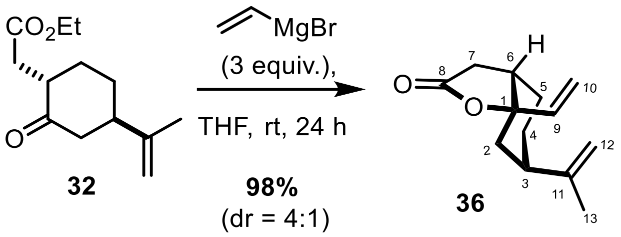

Vinyl magnesium bromide (1.6 M in THF, 3.1 mL, 5.1 mmol, 3 equiv) was added at 0°C to a solution of ester **32** (500 mg, 1.7 mmol, 1 equiv) in THF (10 mL) and the mixture was stirred for 24 hours at room temperature. The reaction was quenched with saturated solution of NH_4Cl. The phases were separated and the aqueous phase was extracted with Et_2O. The organic phases were combined, dried over $MgSO_4$, and solvents were evaporated under reduced pressure. The crude product was purified by column chromatography on silica gel (PE/ diethyl ether 90:10) afforded **36** as mixture of diastereomers lactone (dr = 4:1) as a colorless oil (323 mg, **98%**).

Notes: diastereomers (dr = 4:1) were not separated.

TLC: Rf = 0.8 (10% EtOAc in hexanes, revealed with PMA)
HRMS (ESI): *m/z* calculed for $C_{13}H_{18}NaO_2$ $[M+Na]^+$ = 229.1199, found 229.1193.

Major diastereomer

^{1}H NMR (360 MHz, C_6D_6): δ 5.26 (m, 2H, H_{10}, H_9), 4.83 (dd, *J* = 4.6, 7.0 Hz, 1H, H_{10}), 4.62 (m, 2H, H_{12}), 2.31 (dd, *J* = 6.1, 16.4 Hz, 1H, H_7), 1.99 (m, 2H, H_2, H_6-H_3), 1.67 (d, *J* = 16.4 Hz, 1H, H_7), 1.51 (s, 3H, H_{13}), 1.36 (m, 2H, H_5), 1.27 (m, 1H, H_4), 1.04 (m, 1H, H_2), 0.78 (m, 2H, H_5, H_4) ppm.
^{13}C NMR (90 MHz, C_6D_6): δ 175.4 (C_8), 148.5 (C_{11}), 141.2 (C_9), 113.6 (C_{10}), 109.1 (C_{12}), 85.3 (C_1), 38.9 (C_2), 38.5 (C_3), 37.3 (C_6), 36.7 (C_7), 28.8 (C_5), 28.7 (C_4), 20.6 (C_{13}) ppm.

Compound 37

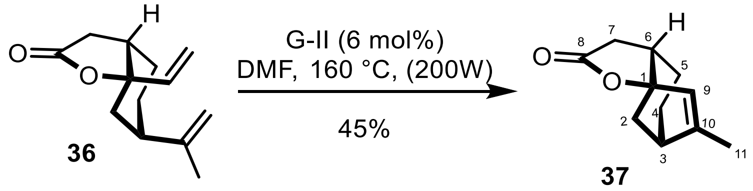

A solution of starting diene **36** (55 mg, 0.24 mmol) in DMF ([C]= 0.05 mol/L, 5 mL) was purged with argon for 15 min. Then Grubbs II catalyst (6% mol) was added under argon and the mixture was stirred at 160 °C under micro-wave (200 W). After 8 h, DMSO (1 mL) was added and the mixture was stirred under air for 24 h and concentrated. The crude reaction mixture was column chromatography on silica (10% EtOAc in PE) to yield lactone bicyclo[3.2.1]octane **37** compound (21 mg, **45%**) as a colorless oil

TLC: Rf = 0.3 (10% EtOAc in PE, revealed with PMA)
HRMS (ESI): *m/z* calculated for $C_{11}H_{15}O_3$ $[M+H]^+$ = 179.1065, found 179.1066.

^{1}H NMR (360 MHz, C_6D_6): δ 5.36 (m, 1H, H_9), 2.25-2.14 (m, H_7), 1.92-1.86 (m, 1H, H_6), 1.85-1.79 (m, 1H, H_2), 1.78-1.62 (m, 3H, H_3-H_7-H_2), 1.50-1.39 (m, 1H, H_5), 1.36 (s, 3H, H_{11}), 1.21-1.05 (m, 2H, H_4-H_5), 0.94-0.83 (m, 1H, H_4) ppm.
^{13}C NMR (90 MHz, C_6D_6): δ 174.8 (C_8), 148.8 (C_{10}), 131.1 (C_9), 91.8 (C_1), 40.8 (C_6), 39.0 (C_2), 37.0 (C_7), 35.1 (C_3), 26.1 (C_5), 21.1 (C_4), 15.2 (C_{11}) ppm.

Compound 39

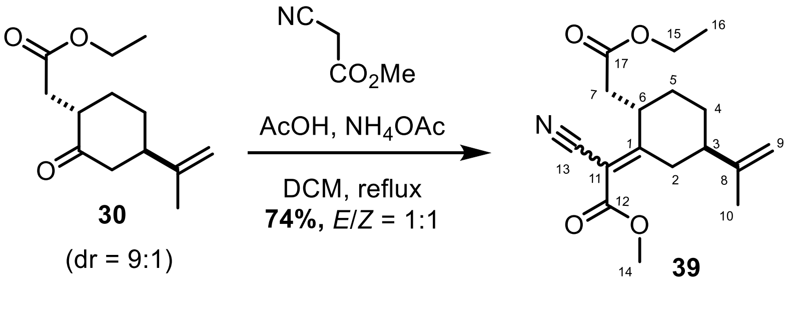

Prepared according to general procedure A. To a solution of ester **30** (500 mg, 2.2 mmol, 1 equiv) in DCM (12 mL) was added methyl cyanoacetate (4.4 mmol, 0.410 mL, 2 equiv), ammonium acetate (508 mg, 6.6 mmol, 3 equiv) and glacial acetic acid (0.264 mL, 4.4 mmol, 2 equiv). The reaction mixture was stirred at reflux and methyl cyanoacetate (4.4 mmol, 0.410 mL, 2 equiv) was added every day until complete

consumption of starting material. Purification by silica gel chromatography (5%-20% EtOAc in PE) afforded the Knoevenagel product **39** as a colorless oil (496 mg, 74%).

Note: 4 days were needed to obtain good conversion. The E/Z (1:1) diastereomers were not separated. Higher temperatures increase acid product formation.

TLC: R_f = 0.6 (10% EtOAc in PE, revealed with PMA, UV).
HRMS (ESI): *m/z* calculed for $C_{17}H_{23}NNaO_4$ $[M+Na]^+$ = 328.1509, found 328.1519.

^{1}H NMR (360 MHz, $CDCl_3$): δ 4.85-4.78 (m, 2H, H_9), 4.52 (m, 1H, $H_{6,}$ *Z*), 4.23-4.09 (m, 2H, H_{15}), 3.98 (m, 1H, H_2, *E*), 3.85 (s, 3H, H_{14}), 3.69 (m, 1H, H_6, *E*), 3.03 (m, 1H, H_2, *Z*), 2.65 (m, 1H, H_7), 2.56 (m, 1H, H_7), 2.44-2.33 (m, 1H, H_2, *Z*), 2.23-1.92 (m, 2H, H_4 or H_5-H_3), 1.78 (s, 3H, H_{10}), 1.76-1.56 (m, 3H, H_4-H_5), 1.27 (m, 3H, H_{16}) ppm.
^{13}C NMR (90 MHz, $CDCl_3$): δ 179.4 (C_1), 170.9 (C_{17}), 162.2 (C_{12}), 147.6 (C_8), 115.1 (C_{13}), 110.2 (C_9), 103.8 (C_{11}), 61.0 (C_{15}), 52.7 (C_{14}), 47.4 (C_3), 40.1 (C_6, *E*), 37 (C_2, *Z*), 36.8 (C_7) 33.8 (C_6, *Z*), 31.7 (C_2, *E*), 30.7 (C_4 or C_5), 25.4 (C_4 or C_5), 20.7 (C_{10}), 14.1 (C_{16}) ppm.

Compound 40

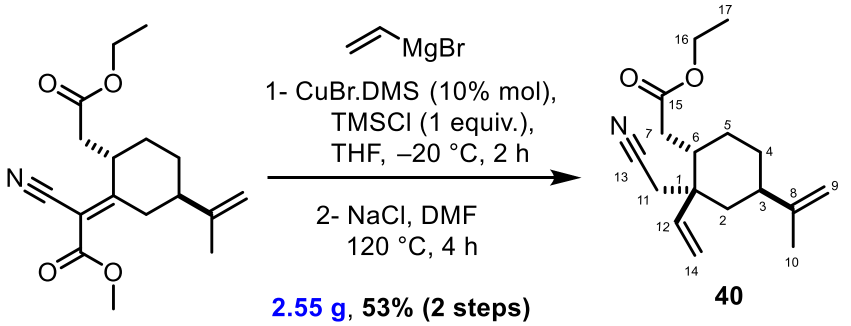

39

E/*Z* = 1:1

40

Vinyl magnesium bromide (1.0 M in THF, 69 mL, 69.2 mmol, 4 equiv) was added dropwise at -20°C to a solution of CuBr.DMS (354 mg, 1.73 mmol, 10% mol), ester **39** (5.28 g, 17.3 mmol, 1 equiv) and TMSCl (2.18 mL, 17.3 mmol, 1 equiv) in THF (150 mL). The mixture was stirred at -20 °C for 2 h. Then the reaction was quenched with saturated solution of NH_4Cl. The phases were separated and the aqueous phase was extracted with Et_2O. The organic phases were combined, dried over $MgSO_4$, and solvents were evaporated under reduced pressure to afford the crude diester.
To the crude in DMF (150 mL) were added NaCl (3.94 g, 6 equiv). Then, the mixture was heated at 120 °C for 3 hours, cooled, H_2O (50 mL) was added and the mixture was extracted with EtOAc. The extracts were washed with brine, dried over $MgSO_4$,

and concentrated under reduced pressure. The product was purified by column chromatography on silica gel using PE/EtOAc (9:1) as eluent to afford the product shown **40** (53% over 2 steps, 2.55 g) as a yellow oil.

Note: Diastereomeric ratio can't be read on the crude directly after the 1,4-addition step. Trace of undesired stereoisomer remain after purification.

TLC: R_f = 0.6 (10% EtOAc in PE, revealed with $KMnO_4$).
HRMS (ESI): *m/z* calculed for $C_{17}H_{26}NO_2$ $[M+Na]^+$ = 276.1951, found 276.1958.

Major diastereomer

^{1}H NMR (360 MHz, $CDCl_3$): δ 5.77 (dd, *J* = 11.1, 17.5 Hz, 1H, H_{12}), 5.26 (d, 11.1 Hz, 1H, H_{14}), 5.15 (d, *J* = 17.5 Hz, 1H, H_{14}), 4.75 (m, 2H, C_9), 4.11 (q, *J* = 7.1 Hz, H_{16}), 2.76 (d, *J* = 17.1 Hz, 1H, H_{11}), 2.48 (d, *J* = 17.1 Hz, 1H, H_{11}), 2.45 (m, 1H, H_2), 2.31 (m, 1H, H_2), 2.19 (m, 1H, H_6), 2.05 (m, 1H, H_3), 1.85 (m, 1H, H_7), 1.74 (s, 3H, H_{10}), 1.67 (m, 3H, H_4-H_5-H_7), 1.48 (m, 1H, H_4-H_5), 1.29 (m, 2H, H_4-H_5), 1.23 (t, *J* = 7.1 Hz, H_{17}) ppm.

^{13}C NMR (90 MHz, $CDCl_3$): δ 172.8 (C_{15}), 148.9 (C8), 142.9 (C_{12}), 117.5 (C_{13}), 115.7 (C_{14}), 109.4 (C_9), 60.5 (C_{16}), 41.6 (C_1), 39.5 (C_3), 36.6 (C_7), 34.5 (C_6), 31.7 (C_2), 26.7 (C_{11}), 25.1 (C_5 or C_4), 24.2 (C_5 or C_4), 21.0 (C_{10}), 14.2 (C_{17}) ppm.

Compound 41

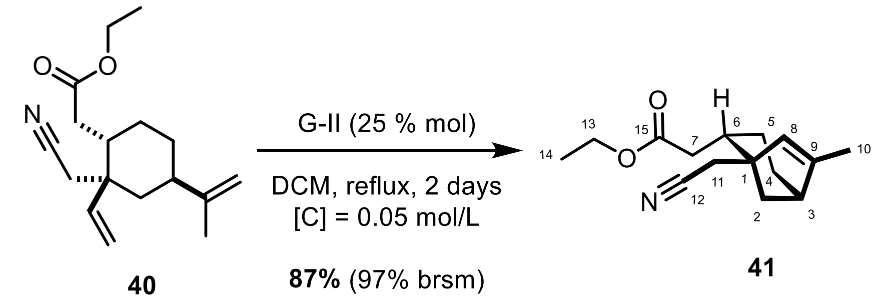

A solution of starting diene **40** (3.6 mmol, 1 g, 1 equiv) in DCM ([C]= 0.05 mol/L, 90 mL) was purged with argon for 15 min. Then Grubbs II catalyst (6% mol) was added under argon and the mixture was stirred at reflux. Several portions of Grubbs II were added every until completion on TLC or NMR. The cyclization was almost completed by using 25% mol of Grubbs II (added in several portions) and 48 h at reflux. Then DMSO (1 mL) was added and the mixture was stirred under air for 24 h and concentrated. The crude reaction mixture was purified by column chromatography on

silica gel (90:5:5 PE/EtOAc/acetone) to yield ester bicyclo[3.2.1]octane **41** (773 mg, **87%, 97% brsm**) as a yellow oil.

TLC: R_f = 0.6 (PE/EtOAc/acetone = 90:5:5, revealed with PMA or $KMnO_4$).
HRMS (ESI): *m/z* calculated for $C_{15}H_{21}NNaO_2$ $[M]^+$ = 270.1455, found 270.1464.

^{1}H NMR (360 MHz, $CDCl_3$): δ 5.15 (m, 1H, H_8), 4.15 (q, *J* = 1.2, 7.5 Hz, 2H, H_{13}), 2.50-2.41 (m, 2H, H_3-H_{11}), 2.47- 2.39 (m, 1H, H_{11} or H_7), 2.38-2.28 (m, 1H, H_{11} or H_7), 2.13-1.99 (m, 2H, H_6-H_2), 1.94-1.85 (m, 1H, H_7), 1.75 (s, 3H, H_{10}), 1.73-1.65 (m, 1H, H_4 or H_5), 1.53 (m, 1H, H_2), 1.50-1.41 (m, 2H, H_4 or H_5), 1.28 (t, *J* = 7.5 Hz, H_{14}), 1.17 (m, 1H, H_4 or H_5) ppm.
^{13}C NMR (90 MHz, $CDCl_3$): δ 172.6 (C_{15}), 146.5 (C_9), 127.7 (C_8), 117.8 (C_{12}), 60.6 (C_{13}), 49.5 (C_2), 49.0 (C_1), 44.2 (C_3), 37.9 (C_7), 36.9 (C_6), 34.8 (C_1), 26.4 (C_5), 25.0 (C_{11}), 23.5 (C_4), 15.2 (C_{10}), 14.5 (C_{14}) ppm.

Compound 1

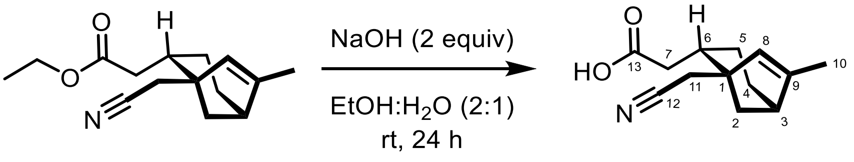

41 1

Ester **41** (0.371 g, 1.29 mmol, 1 equiv) was dissolved in a mixture of EtOH (43 mL) and water (21 mL). Sodium hydroxide (0.103 g, 2.60 mmol, 2 equiv) was added, and the mixture was stirred at rt for 24 h. The reaction mixture was concentrated, resuspended in water, and acidified with 1 M HCl. The aqueous layer was then extracted into EtOAc, dried over anhydrous magnesium sulfate and evaporated to dryness. The crude product was purified by silica gel chromatography (10-20% MeOH in DCM) to give a yellow oil **1** (0.282 g, ***quant.***).

TLC: R_f = 0.2 (DCM/MeOH = 90:10, revealed with PMA or $KMnO_4$).
HRMS (ESI): *m/z* calculated for $C_{13}H_{16}NO_2$ $[M\text{-}H]^+$ = 218.1184, found 218.1186.

^{1}H NMR (360 MHz, $CDCl_3$): δ 5.15 (m, 1H, H_8), 2.49-2.46 (m, 2H, H_{11}), 2.44 (m, 1H, H_3), 2.41-2.34 (dd, *J* = 4.8, 14.3 Hz, 1H, H_7), 2.04 (m, 1H, H_2), 1.98 (m, 1H, H_6), 1.93-1.84 (dd, *J* = 8.3, 14.3 Hz, 1H, H_7), 1.79-1.75 (m, 1H, H_4 or H_5), 1.74 (s, 3H, H_{10}), 1.47 (m, 1H, H_2), 1.42 (m, 2H, H_4 or H_5), 1.13 (m, 1H, H_4 or H_5) ppm.

^{13}**C NMR** (90 MHz, $CDCl_3$): δ 179.0 (C_{13}), 146.9 (C_9), 123.3 (C_8), 118.0 (C_{12}), 49.5 (C_2), 49.2 (C_1) 44.3 (C_3), 37.5 (C_7), 36.4 (C_6), 26.8 (C_5), 25.1 (C_{11}), 23.5 (C_4), 15.4 (C_{10}) ppm.

Copy of NMR spectras

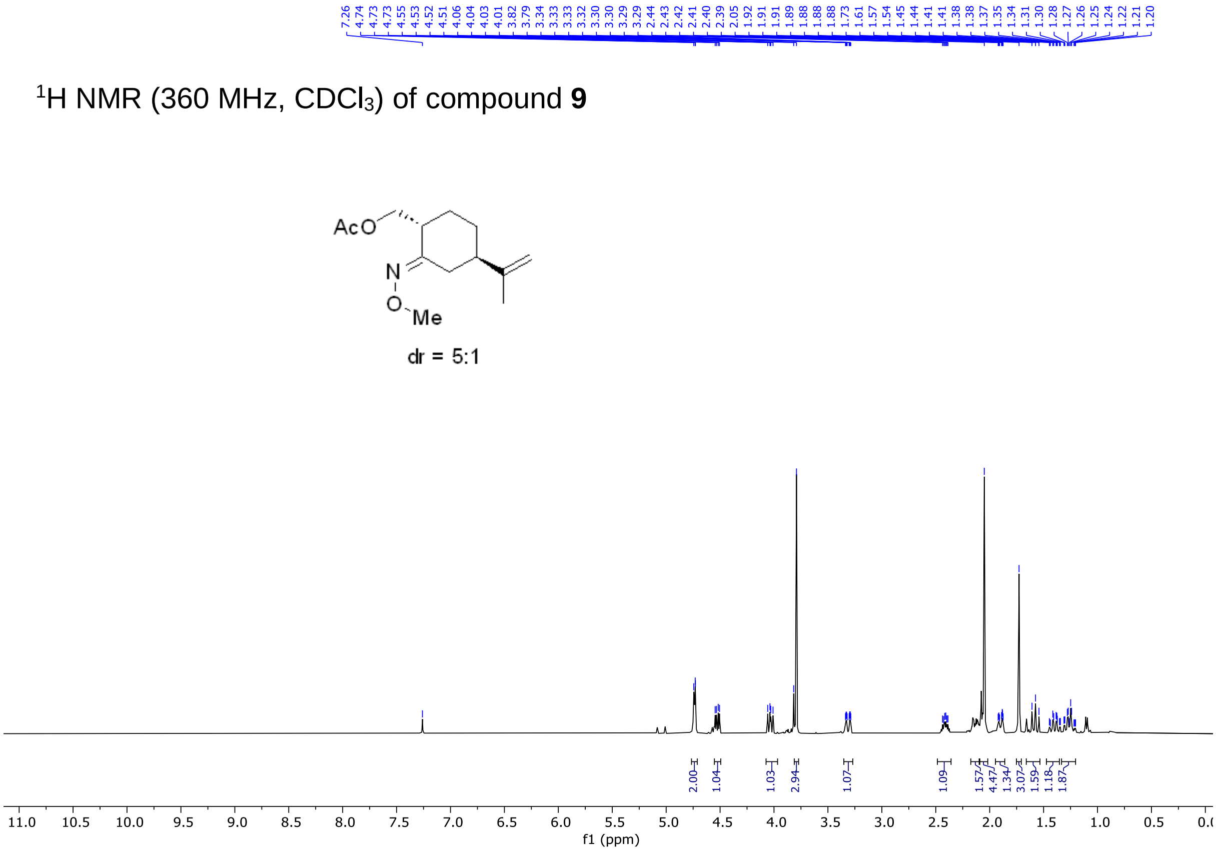

^1H NMR (360 MHz, $CDCl_3$) of compound **9**

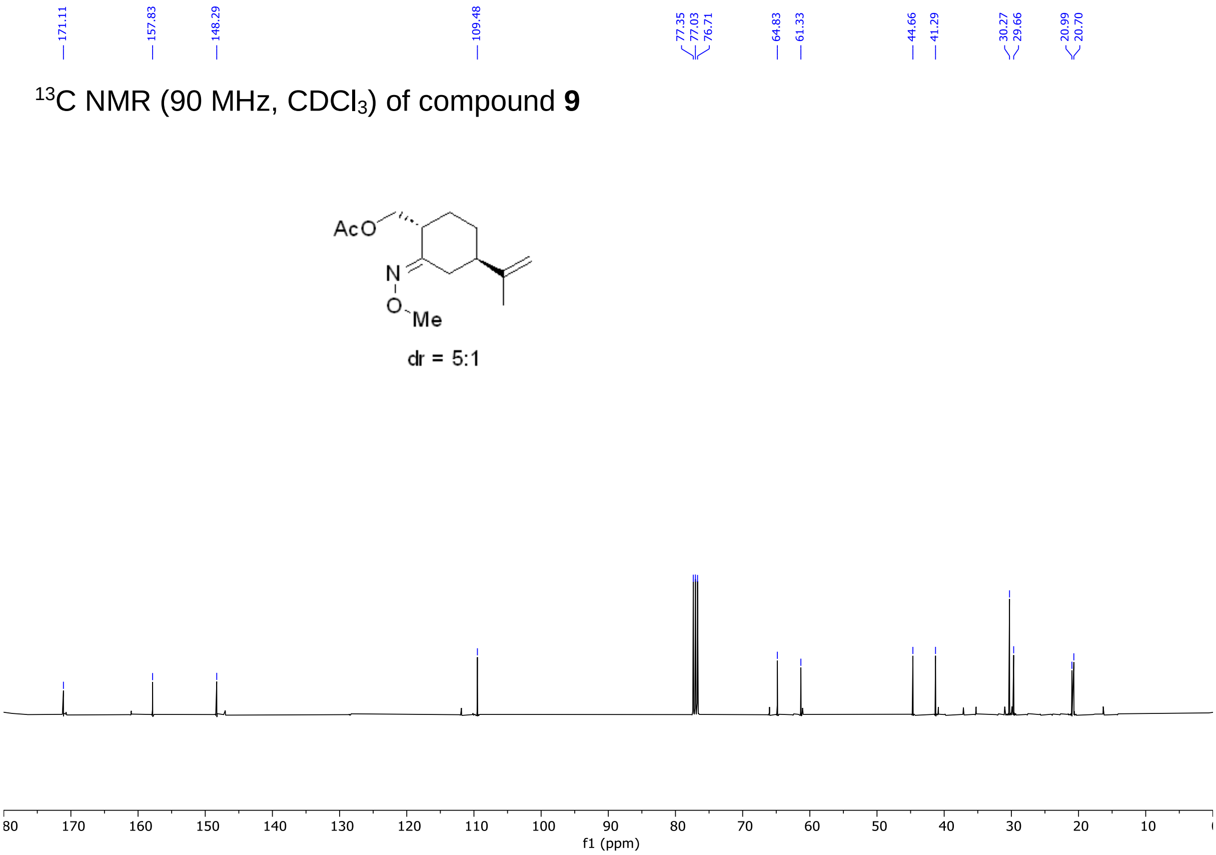

^{13}C NMR (90 MHz, $CDCl_3$) of compound **9**

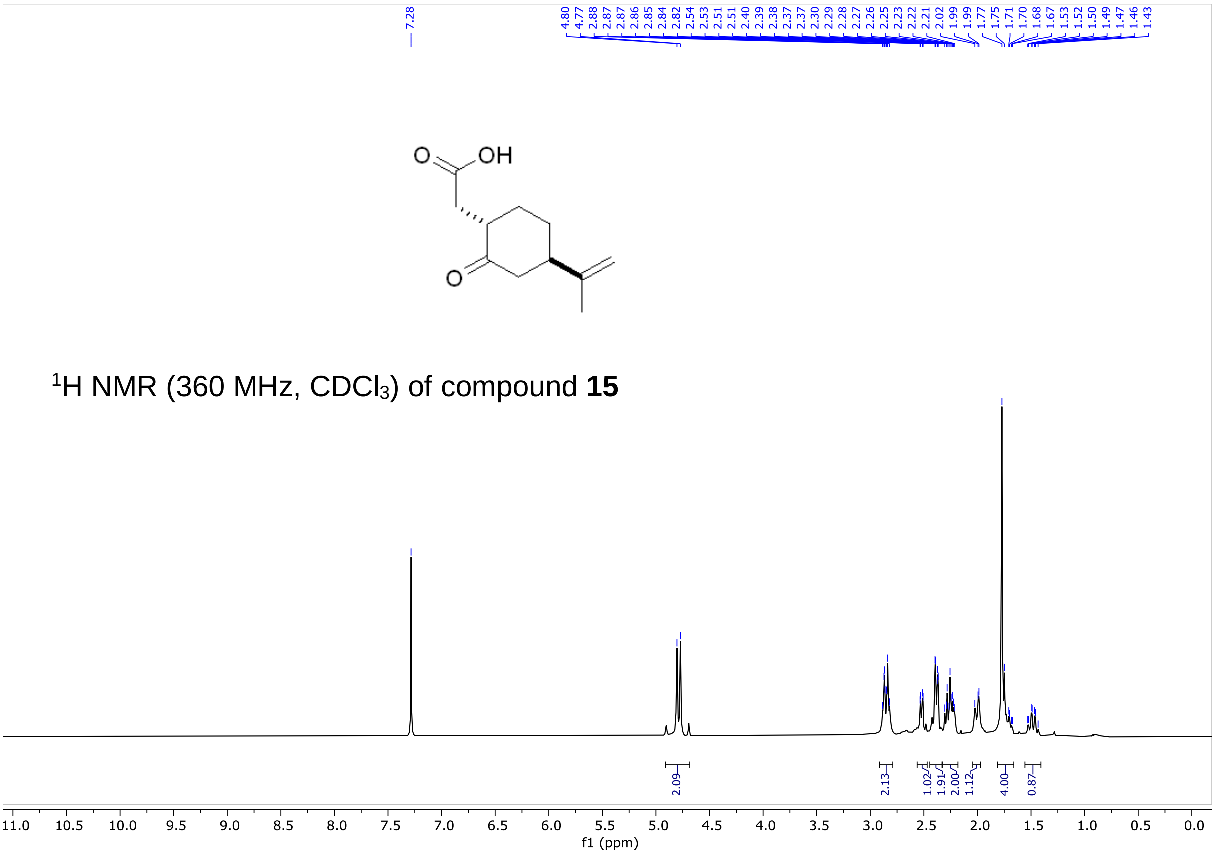

^{1}H NMR (360 MHz, $CDCl_3$) of compound **15**

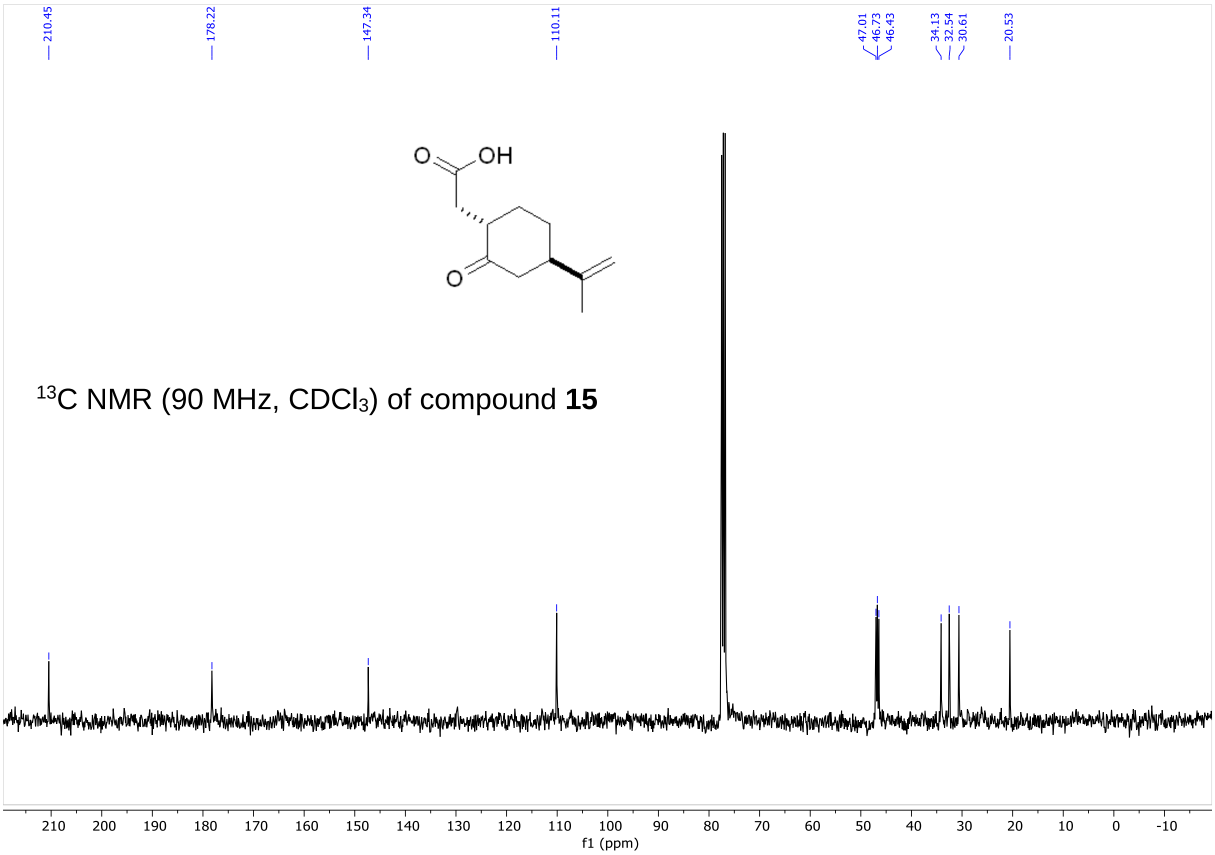

^{13}C NMR (90 MHz, $CDCl_3$) of compound **15**

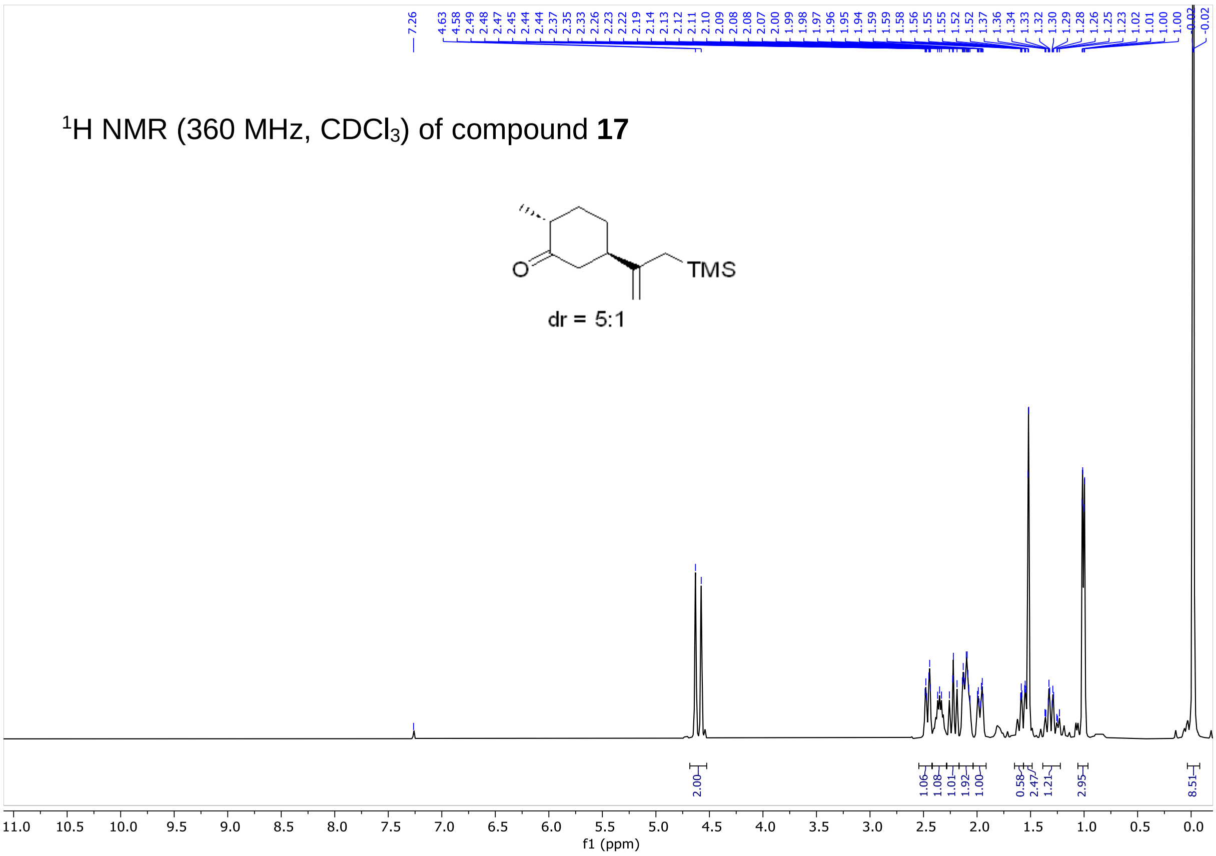

7.26
4.63 4.58 2.49 2.48 2.47 2.45 2.44 2.44 2.37 2.35 2.33 2.26 2.23 2.22 2.19 2.14 2.13 2.12 2.11 2.10 2.09 2.08 2.08 2.07 2.00 1.99 1.98 1.97 1.96 1.95 1.94 1.59 1.59 1.58 1.56 1.55 1.55 1.52 1.52 1.37 1.36 1.34 1.33 1.32 1.30 1.29 1.28 1.26 1.25 1.23 1.02 1.01 1.00 1.00 0.02 -0.02
^{1}H NMR (360 MHz, CDCl$_3$) of compound **17**
TMS
dr = 5:1
2.00
1.06 1.08 1.01 1.92 1.00
0.58 2.47 1.21
2.95
8.51
f1 (ppm)

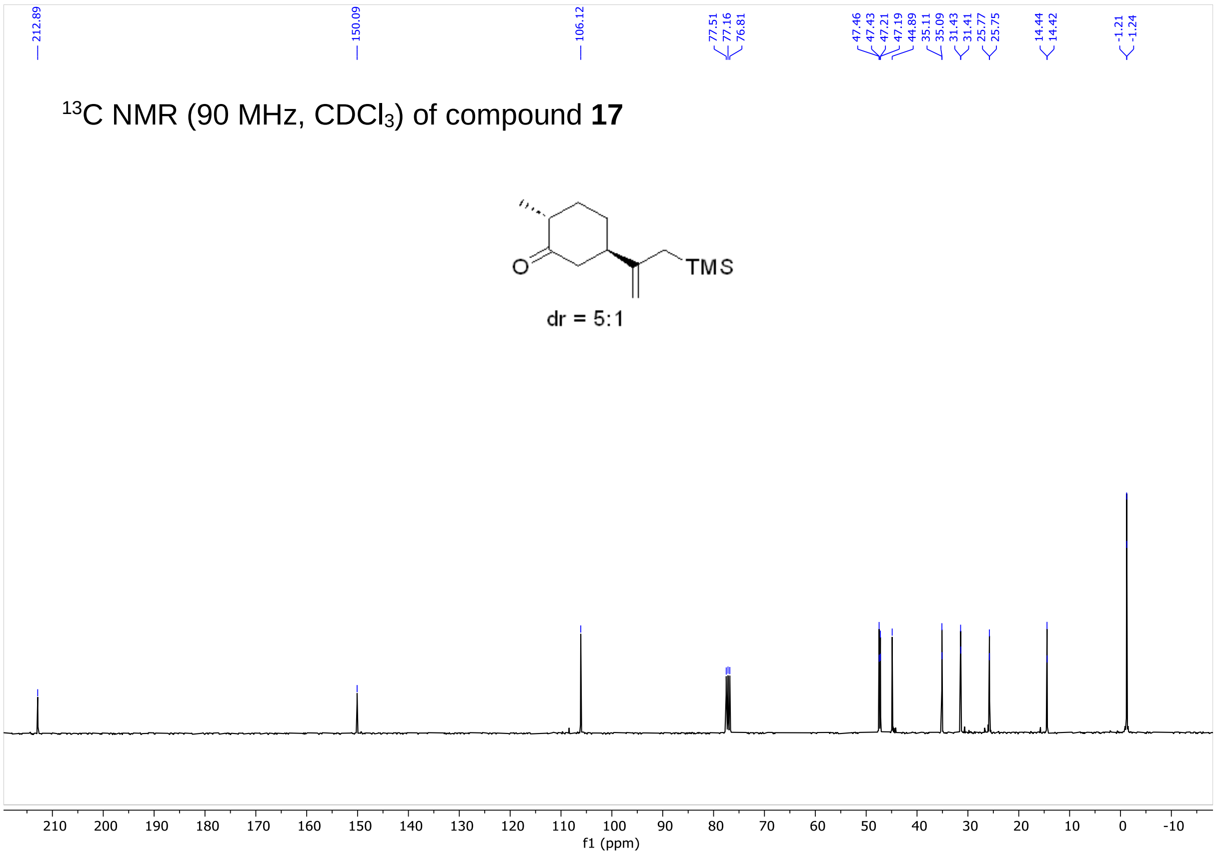

212.89
150.09
106.12
77.51 77.16 76.81
47.46 47.43 47.21 47.19 44.89 35.11 35.09 31.43 31.41 25.77 25.75
14.44 14.42
-1.21 -1.24
^{13}C NMR (90 MHz, CDCl$_3$) of compound **17**
TMS
dr = 5:1
f1 (ppm)

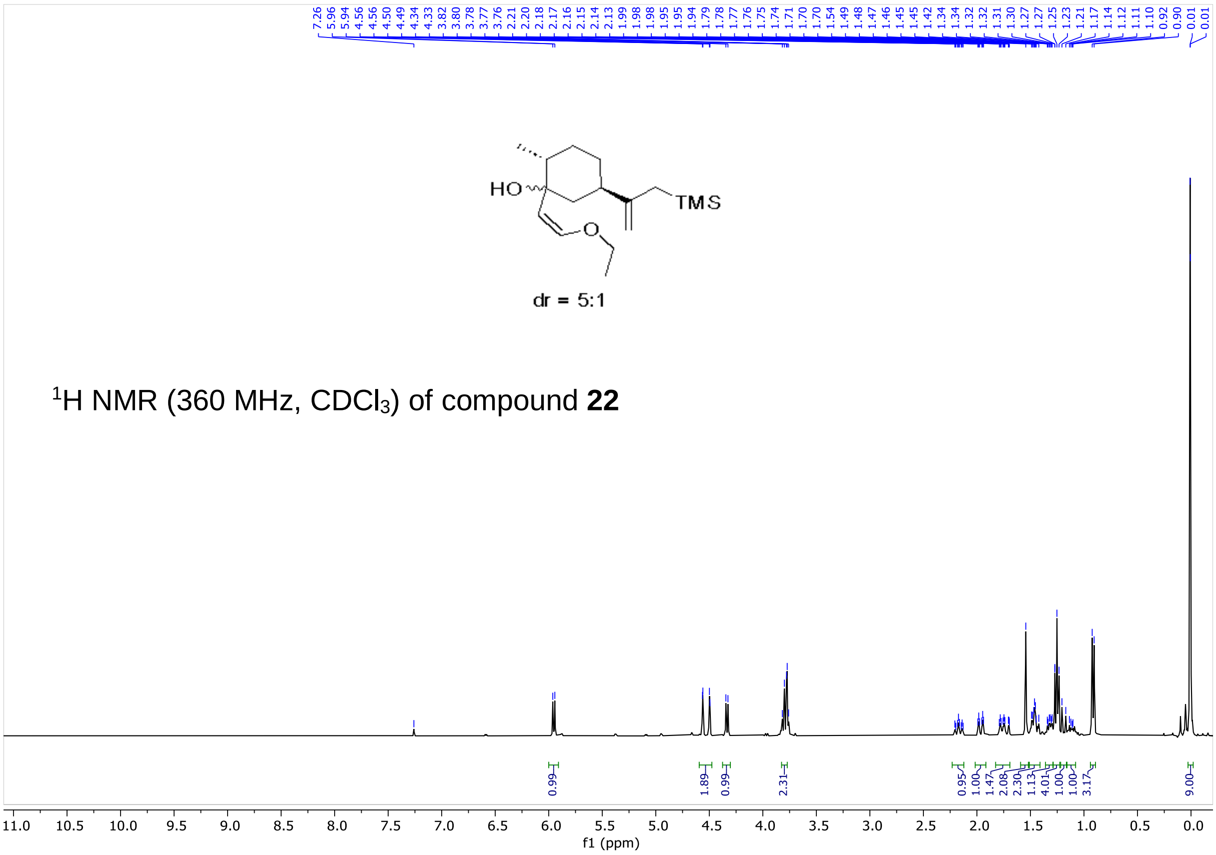

^{1}H NMR (360 MHz, $CDCl_3$) of compound **22**

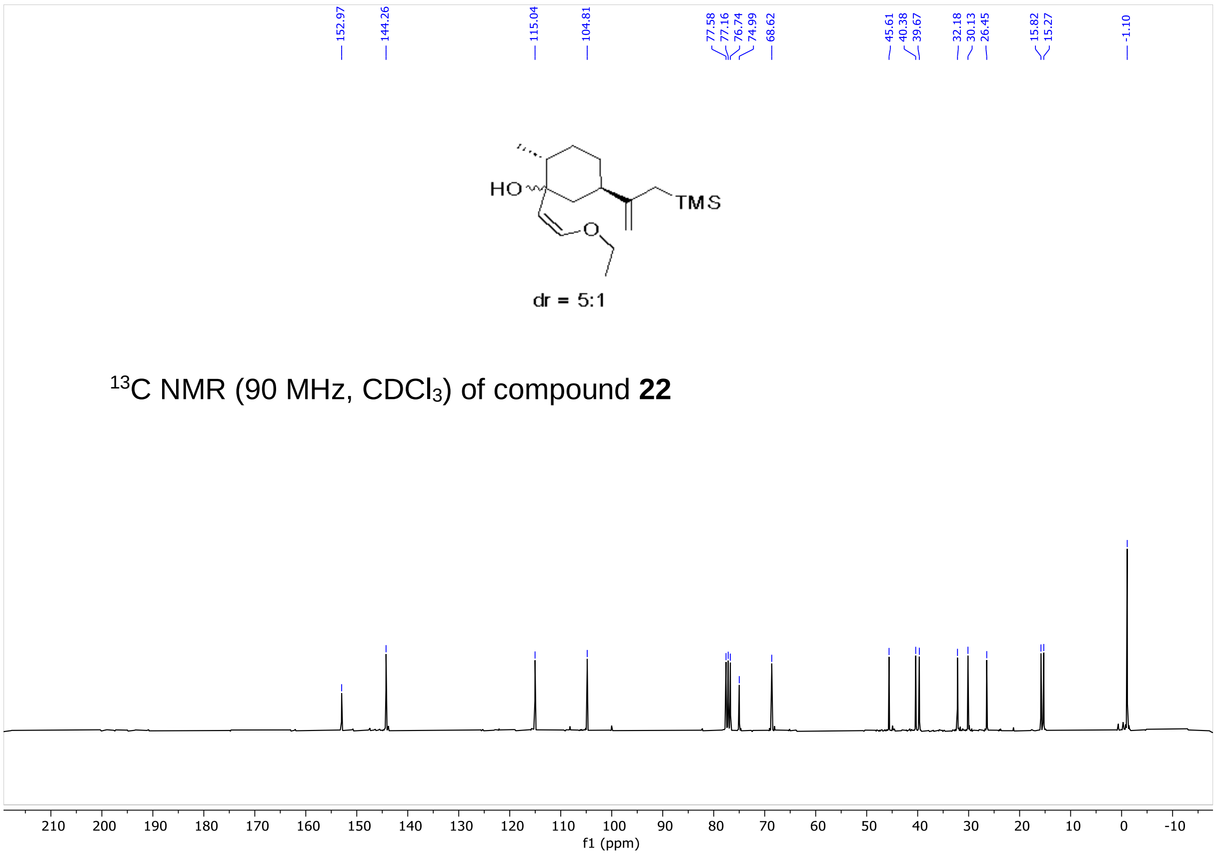

^{13}C NMR (90 MHz, $CDCl_3$) of compound **22**

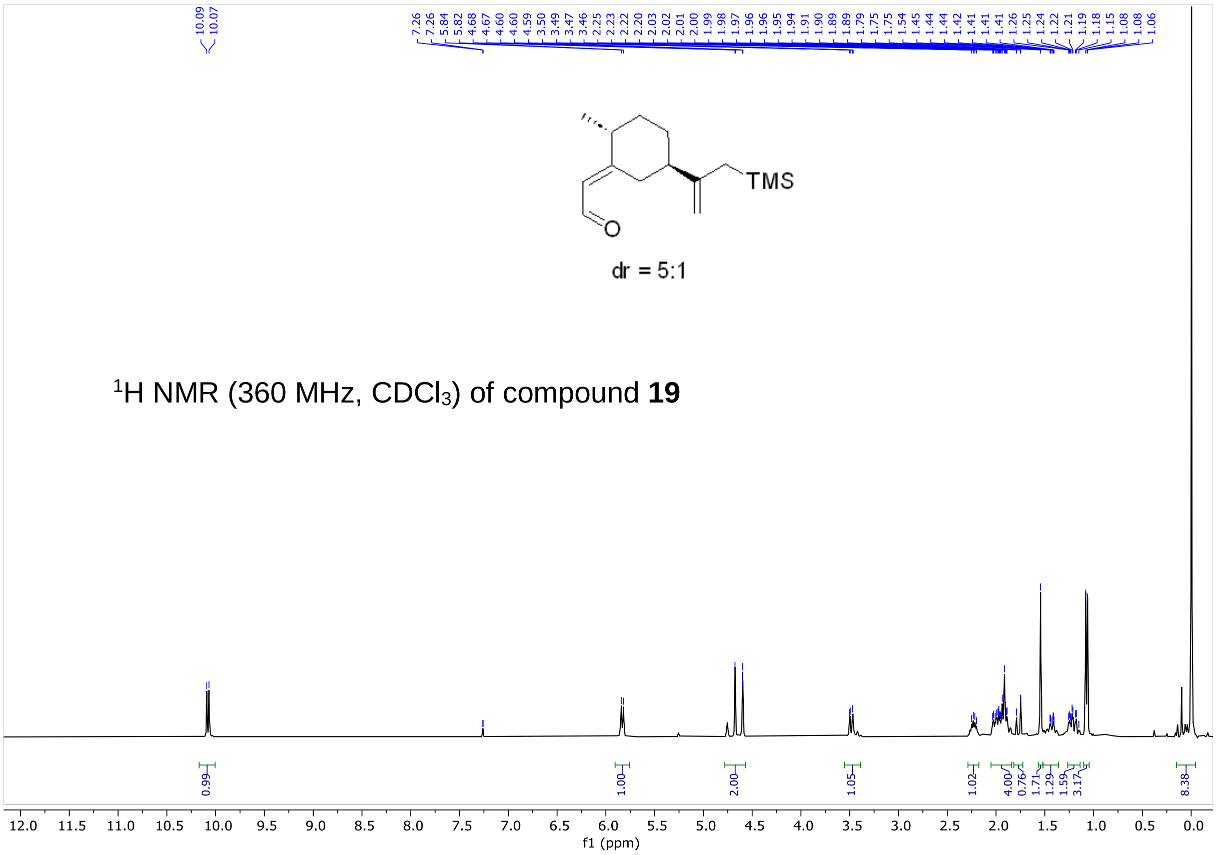

^{1}H NMR (360 MHz, $CDCl_3$) of compound **19**

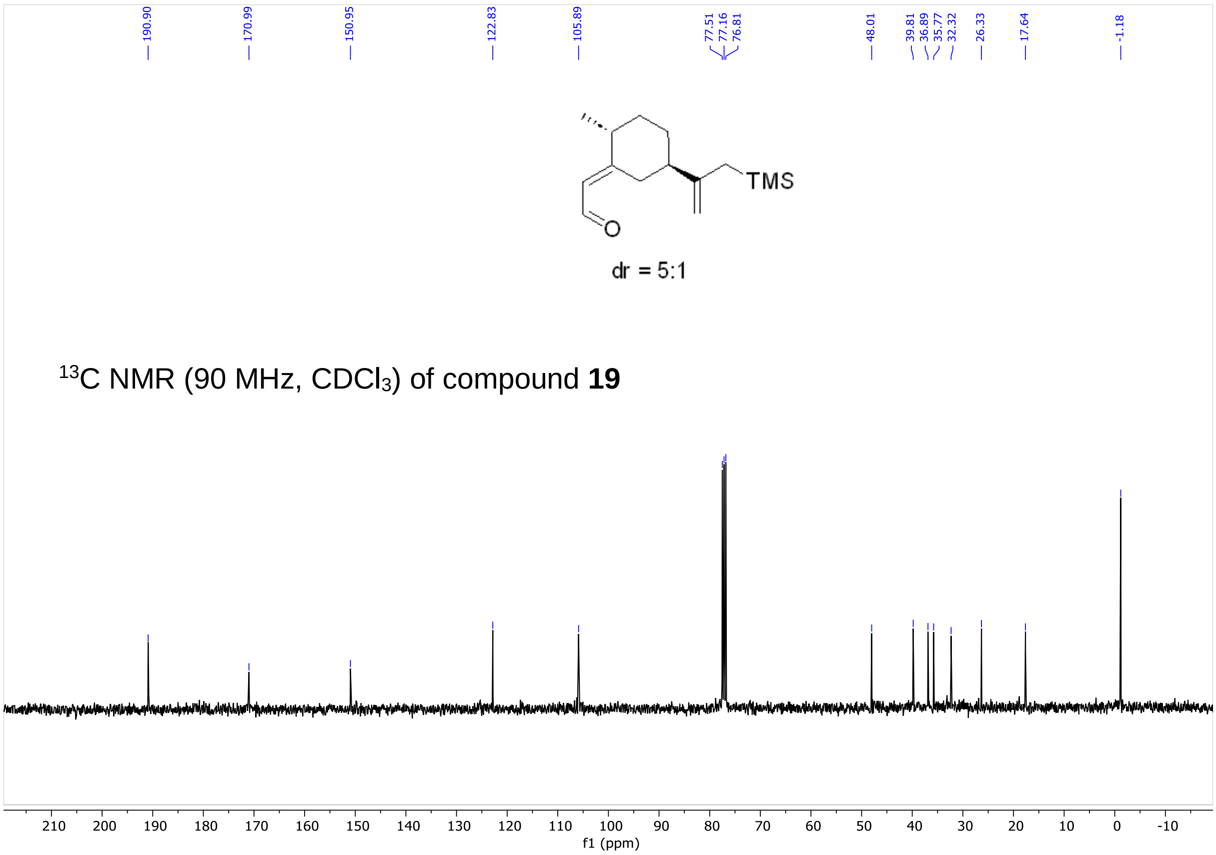

^{13}C NMR (90 MHz, $CDCl_3$) of compound **19**

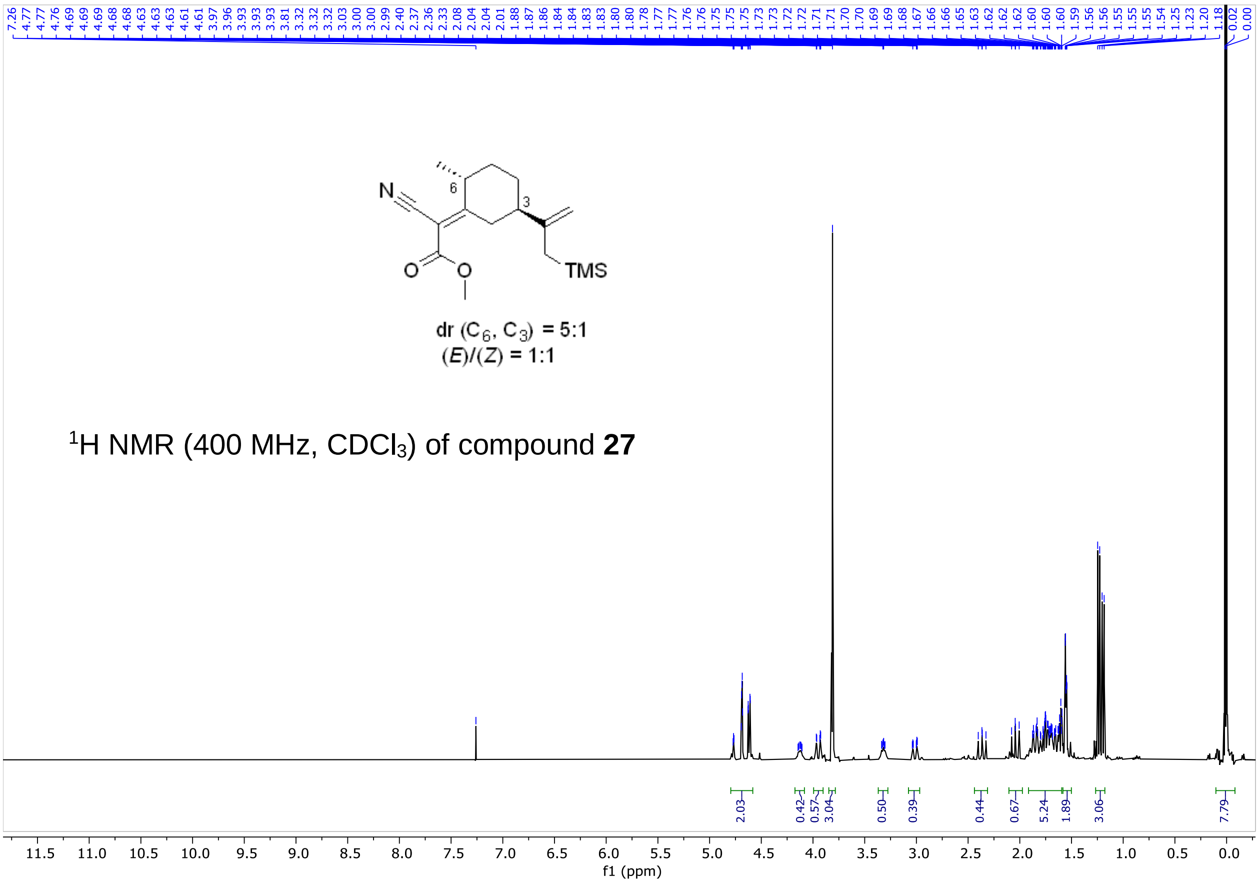

^{1}H NMR (400 MHz, $CDCl_3$) of compound **27**

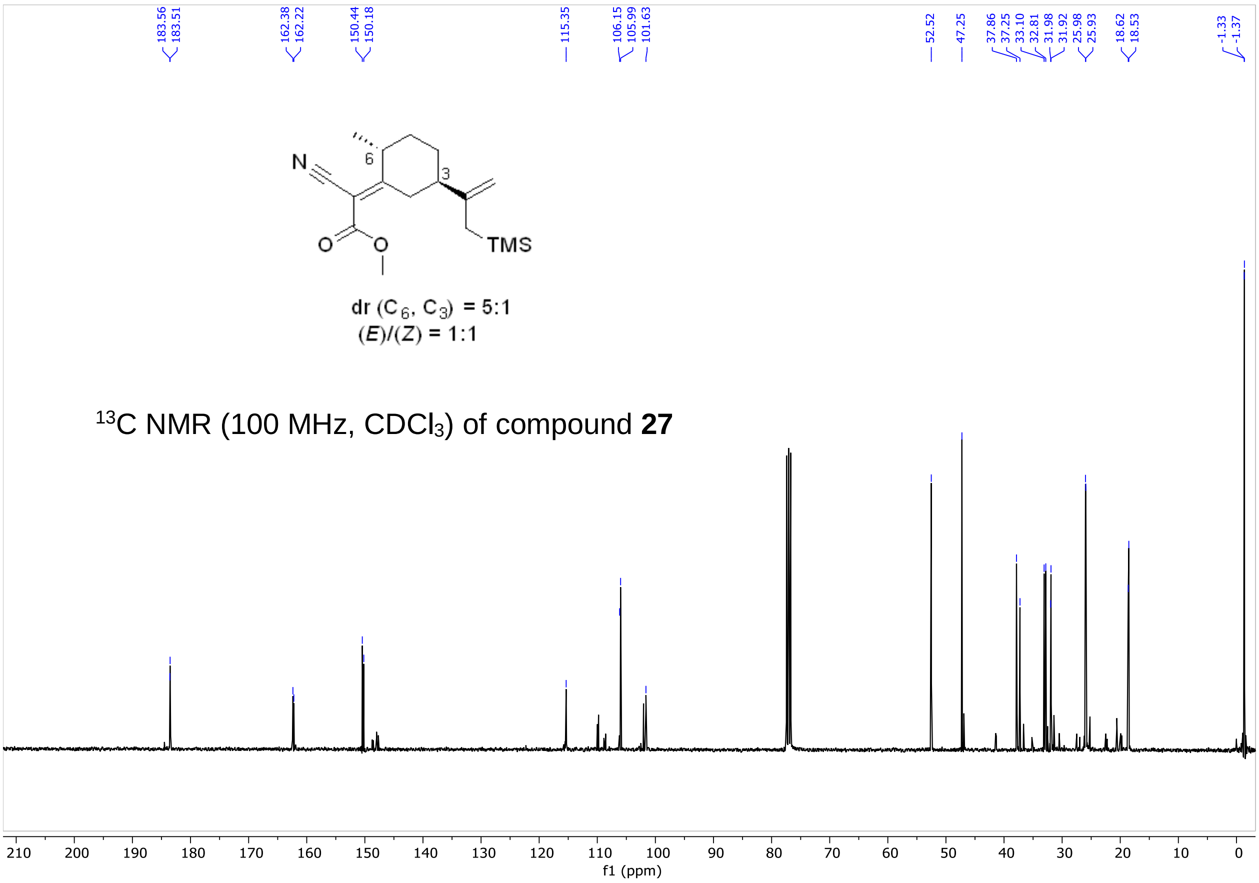

^{13}C NMR (100 MHz, $CDCl_3$) of compound **27**

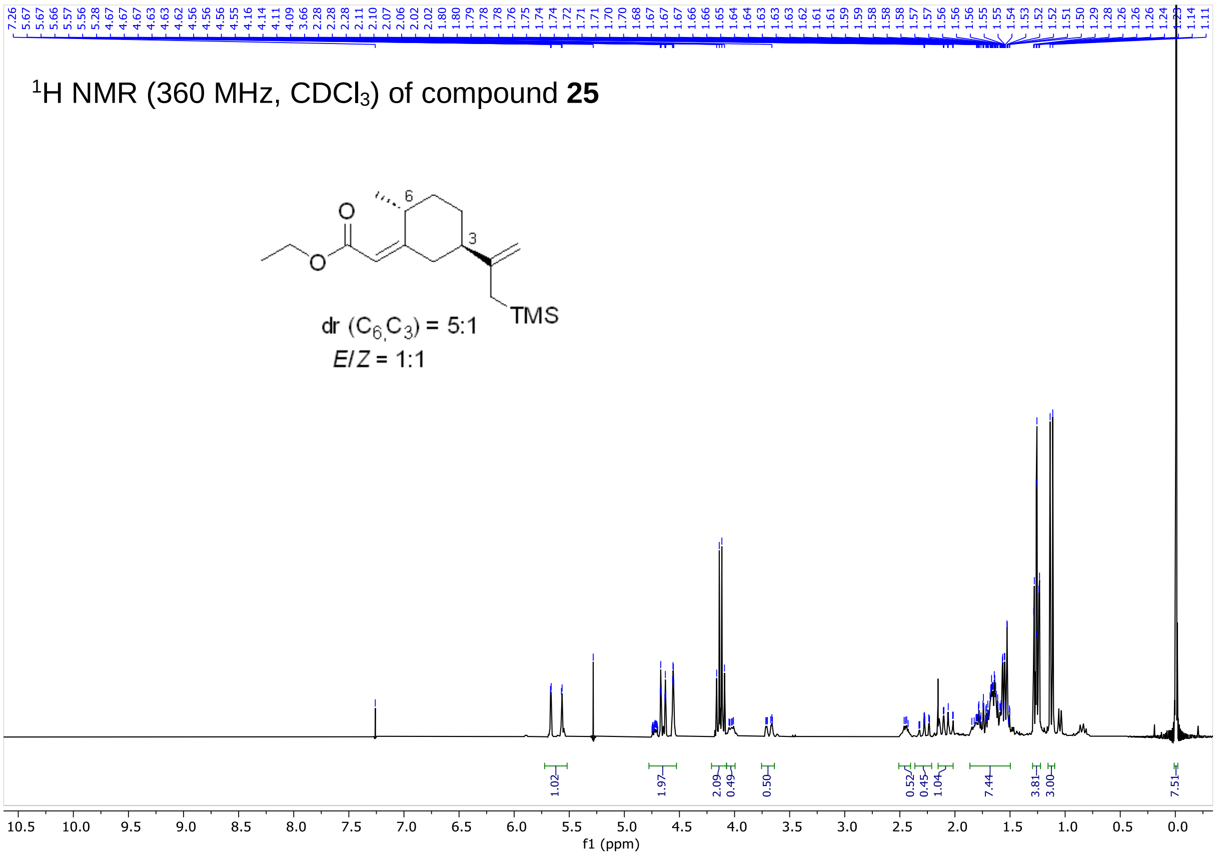

1H NMR (360 MHz, CDCl3) of compound 25
6
3
O
O
TMS
dr (C6,C3) = 5:1
E/Z = 1:1
f1 (ppm)

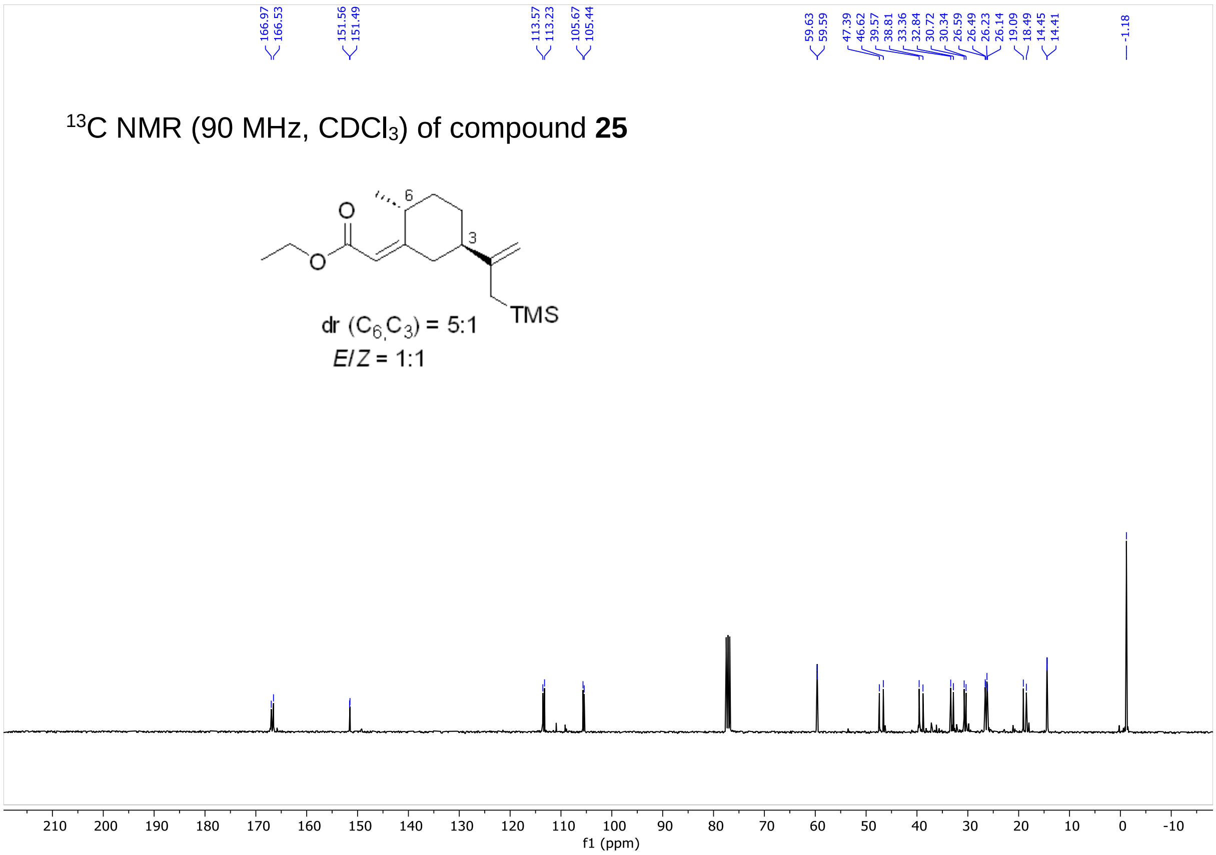

13C NMR (90 MHz, CDCl3) of compound 25
6
3
O
O
TMS
dr (C6,C3) = 5:1
E/Z = 1:1
f1 (ppm)

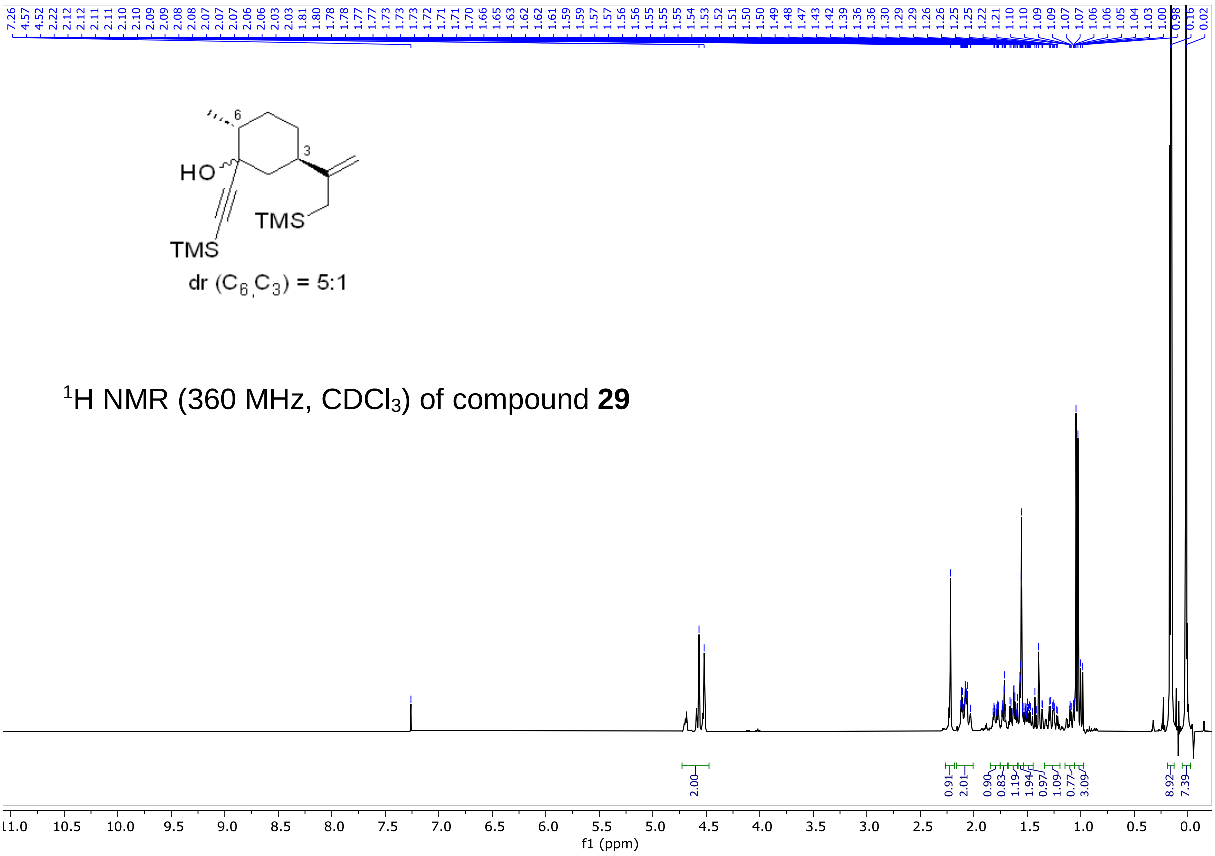
6
3
HO
TMS
TMS
dr (C6,C3) = 5:1
1H NMR (360 MHz, CDCl3) of compound 29
f1 (ppm)

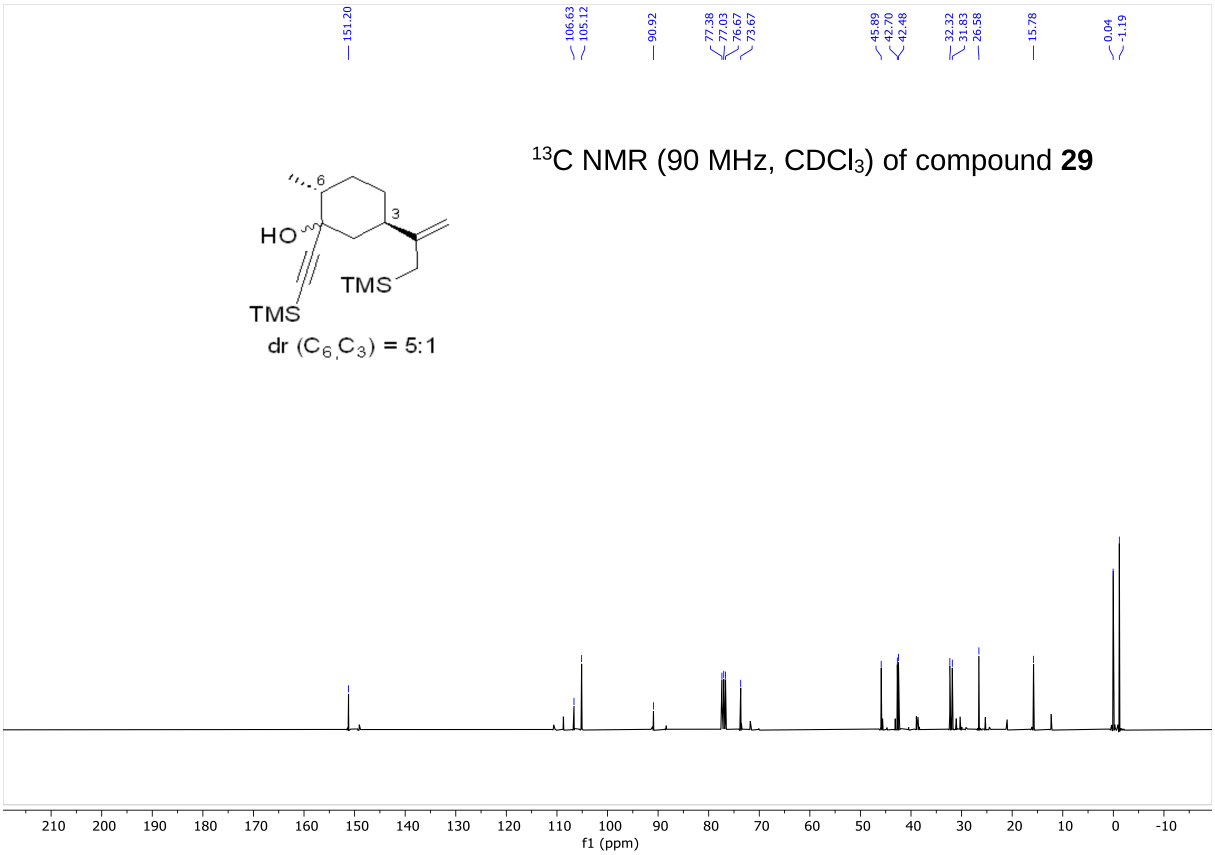
151.20
106.63
105.12
90.92
77.38
77.03
76.67
73.67
45.89
42.70
42.48
32.32
31.83
26.58
15.78
0.04
-1.19
13C NMR (90 MHz, CDCl3) of compound 29
6
3
HO
TMS
TMS
dr (C6,C3) = 5:1
f1 (ppm)

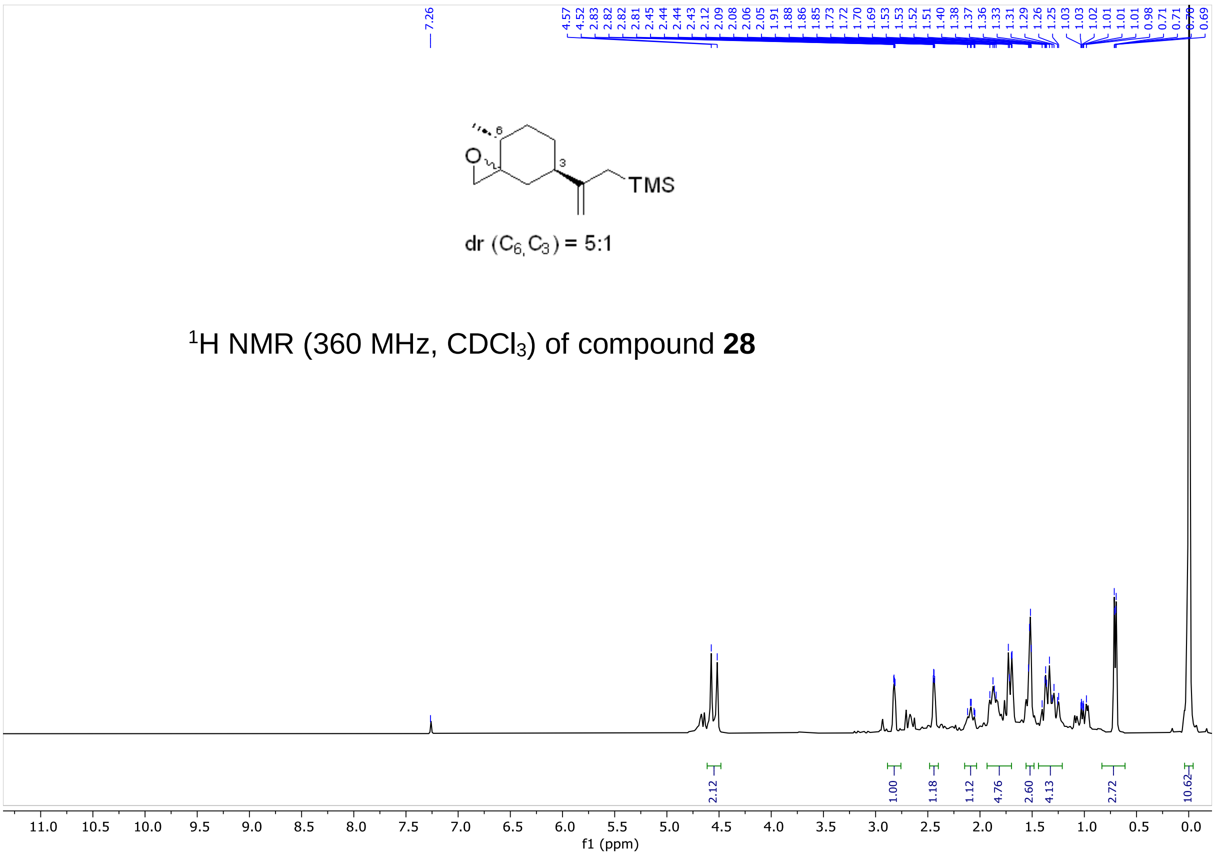
7.26
4.57 4.52 2.83 2.82 2.82 2.81 2.45 2.44 2.44 2.43 2.12 2.09 2.08 2.06 2.05 1.91 1.88 1.86 1.85 1.73 1.72 1.70 1.69 1.53 1.53 1.52 1.51 1.40 1.38 1.37 1.36 1.33 1.31 1.29 1.26 1.25 1.03 1.03 1.02 1.01 1.01 1.01 0.98 0.71 0.71 0.70 0.69
O
6
3
TMS
dr (C6, C3) = 5:1
1H NMR (360 MHz, CDCl3) of compound 28
2.12 1.00 1.18 1.12 4.76 2.60 4.13 2.72 10.62
11.0 10.5 10.0 9.5 9.0 8.5 8.0 7.5 7.0 6.5 6.0 5.5 5.0 4.5 4.0 3.5 3.0 2.5 2.0 1.5 1.0 0.5 0.0
f1 (ppm)

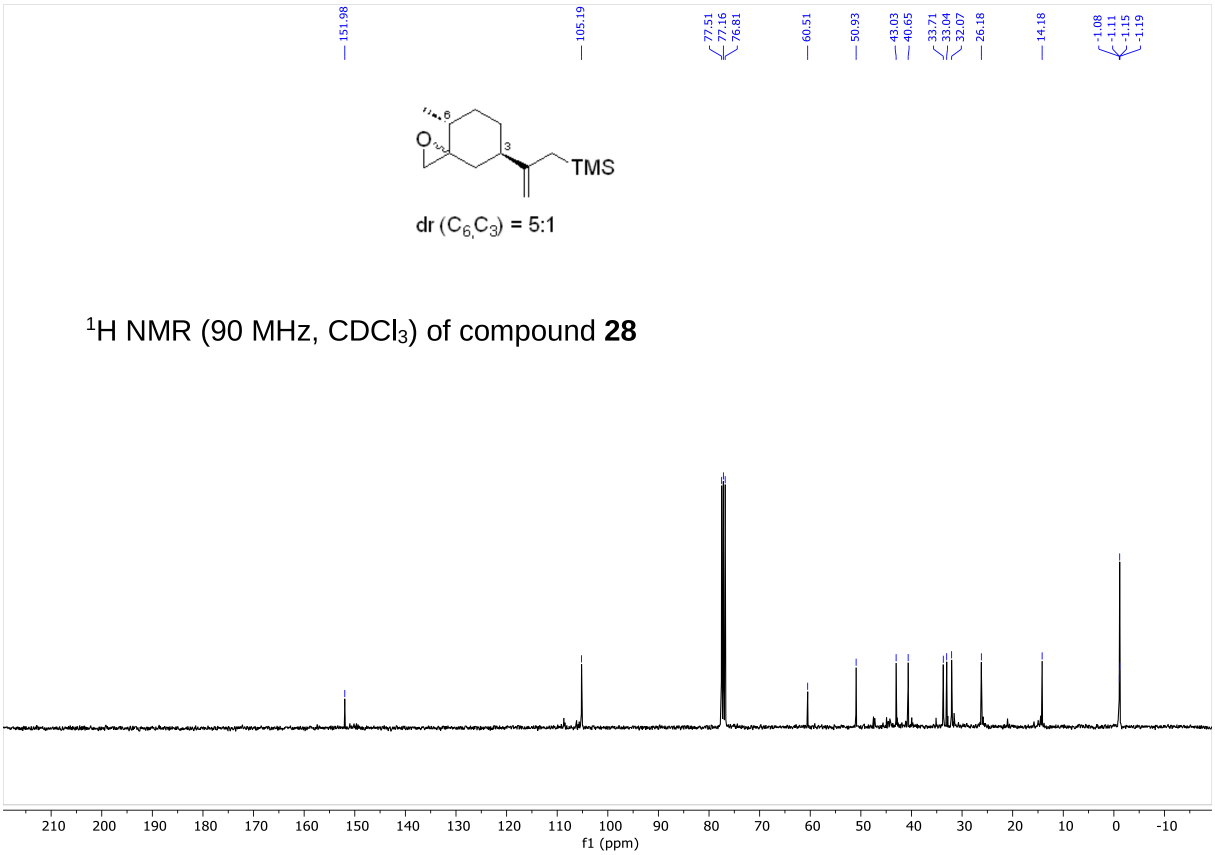
151.98
105.19
77.51 77.16 76.81
60.51
50.93
43.03 40.65
33.71 33.04 32.07
26.18
14.18
-1.08 -1.11 -1.15 -1.19
O
6
3
TMS
dr (C6, C3) = 5:1
1H NMR (90 MHz, CDCl3) of compound 28
210 200 190 180 170 160 150 140 130 120 110 100 90 80 70 60 50 40 30 20 10 0 -10
f1 (ppm)

^{1}H NMR (360 MHz, $CDCl_3$) of compound **32** (*cis*)

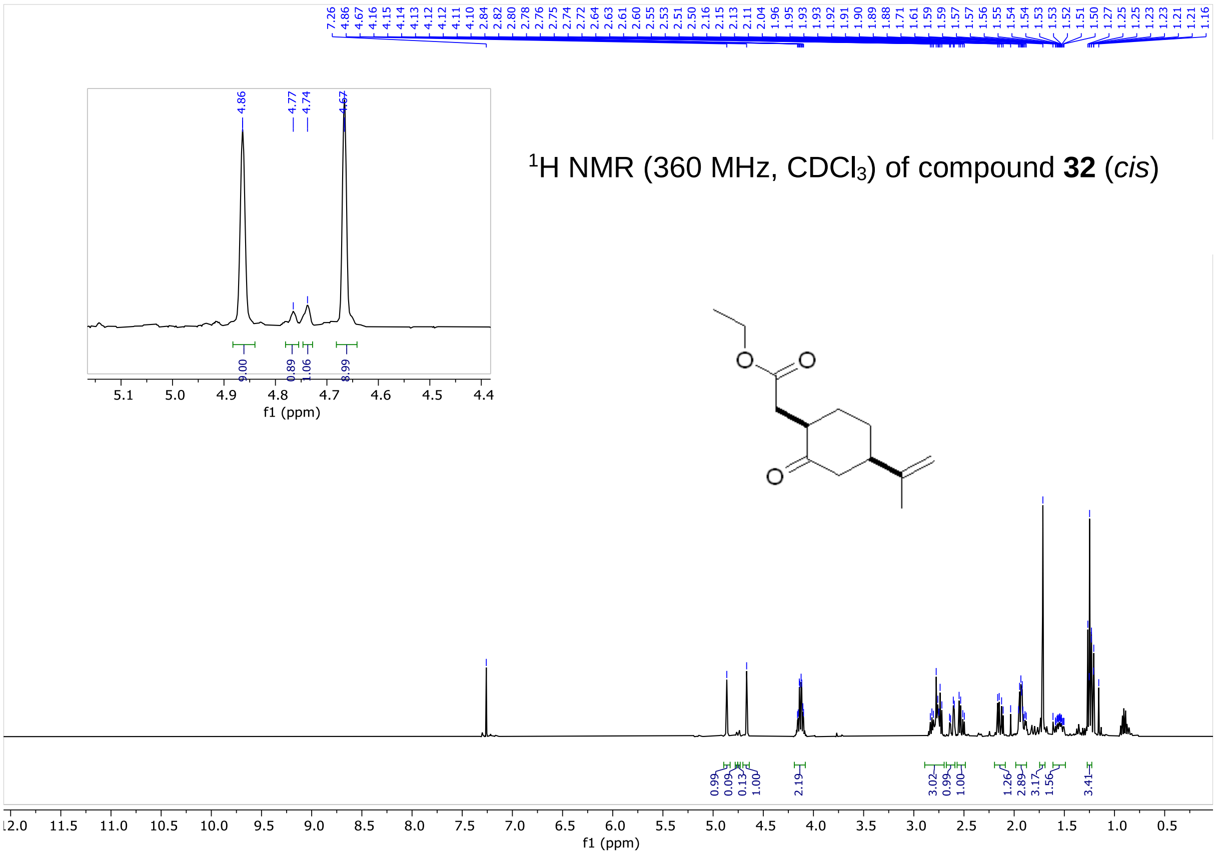

^{1}H NMR (360 MHz, $CDCl_3$) of compound **32** (*trans*)

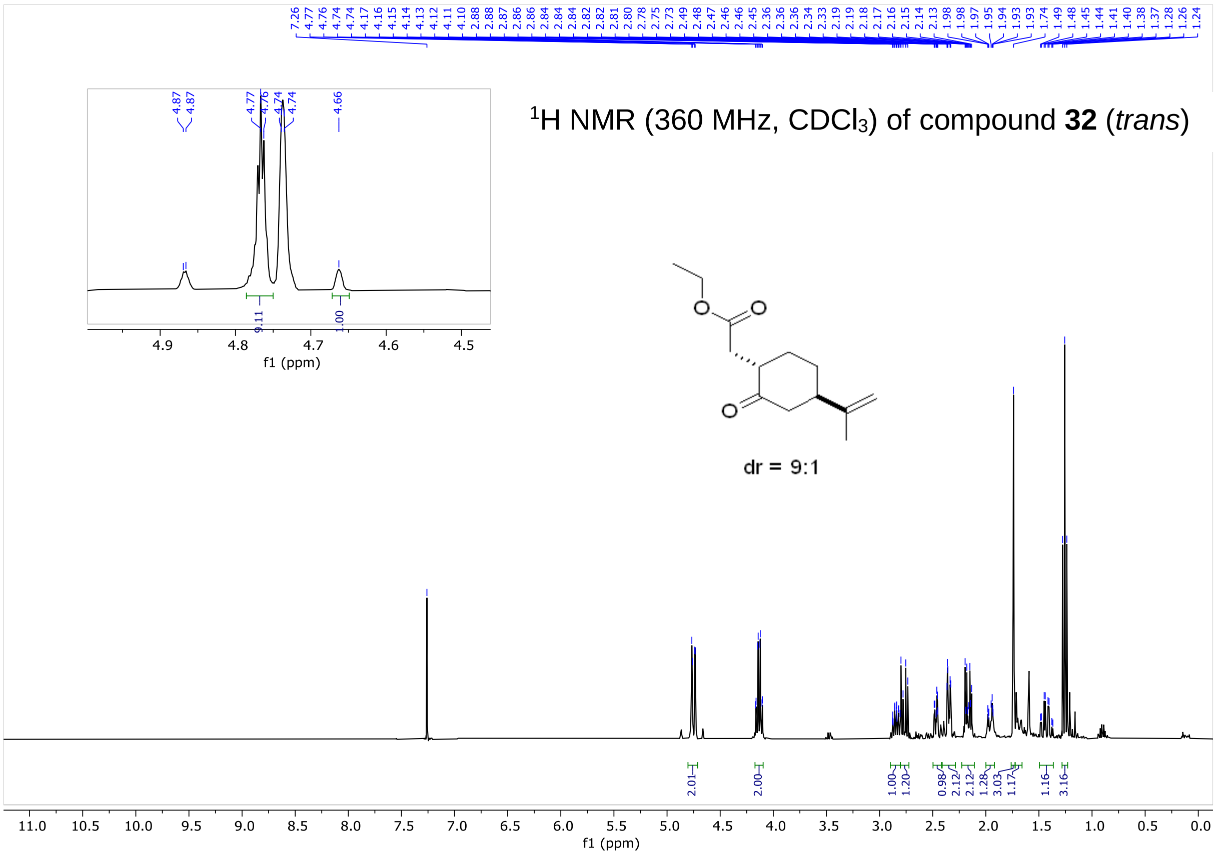

^{13}C NMR (90 MHz, $CDCl_3$) of compound **32** (*trans*)

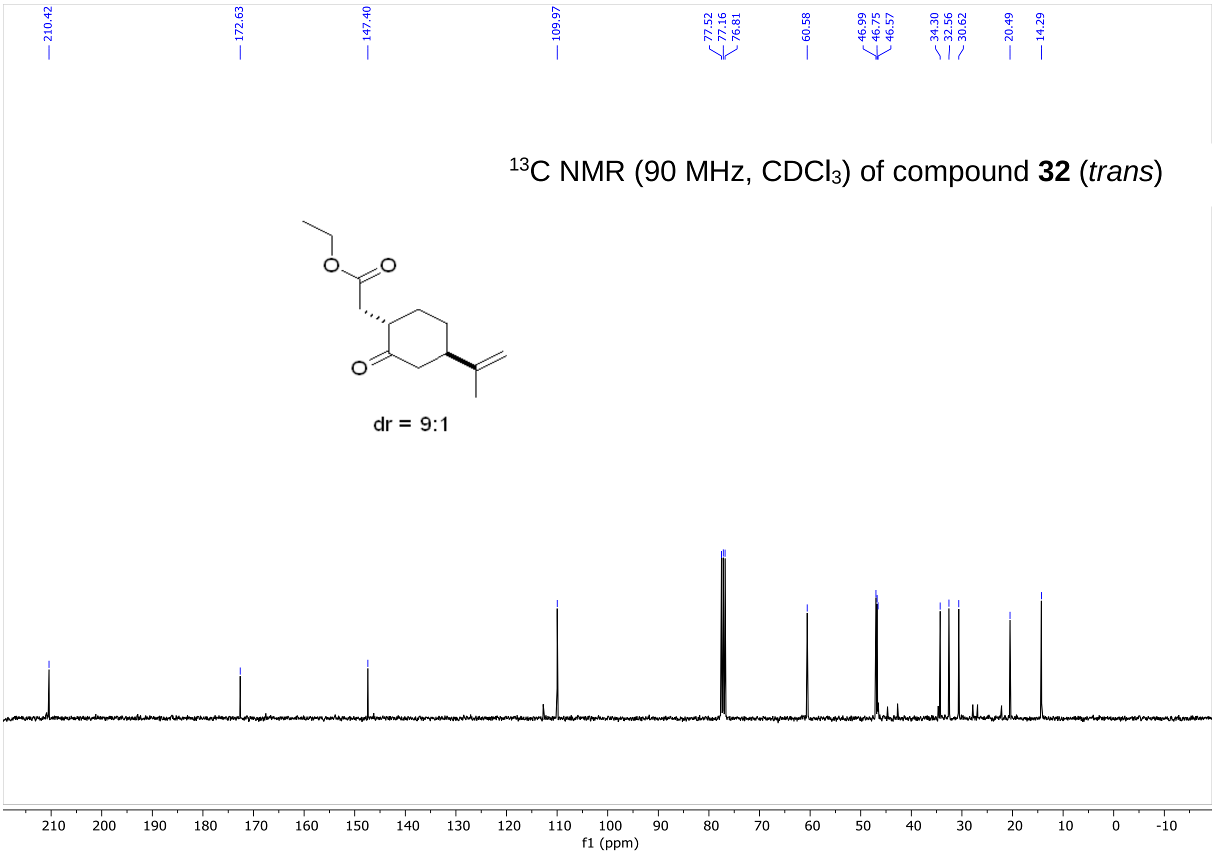

^{1}H NMR (360 MHz, C_6D_6) of compound **36**

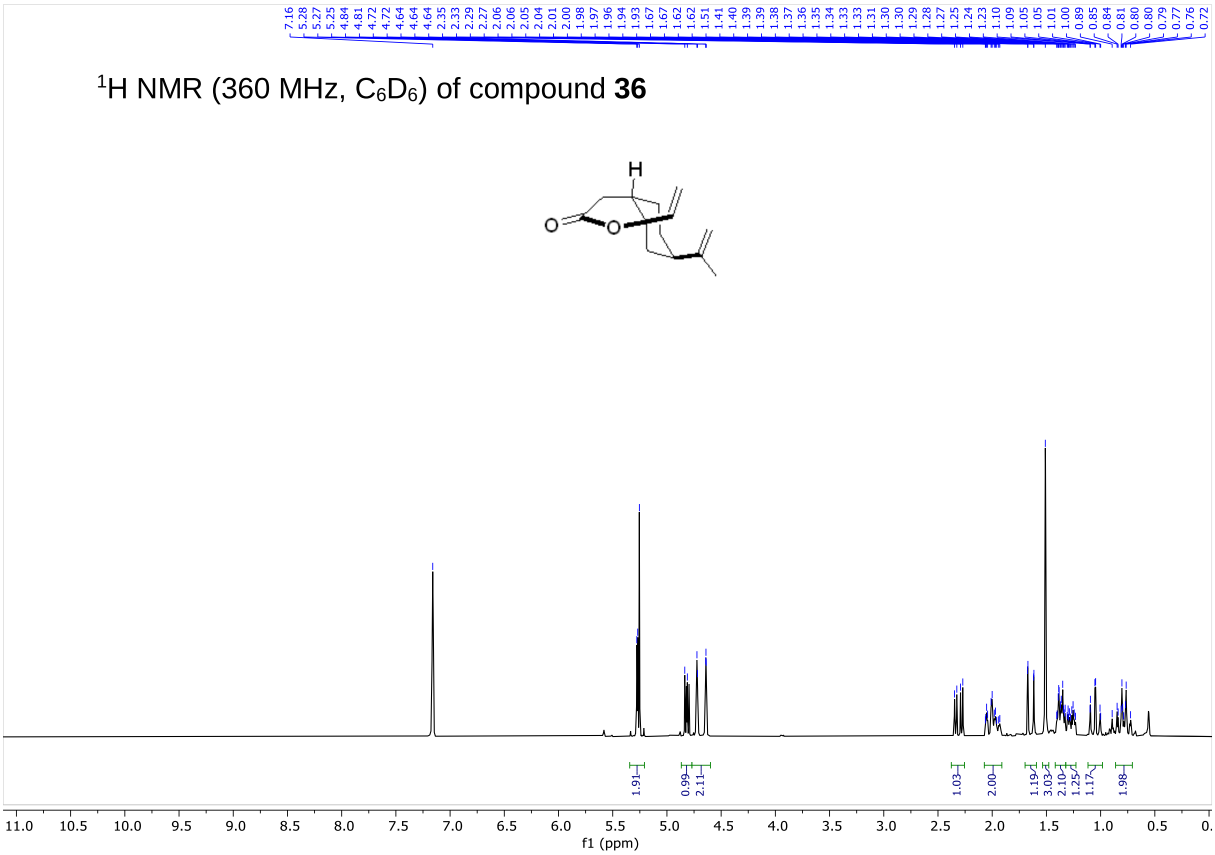

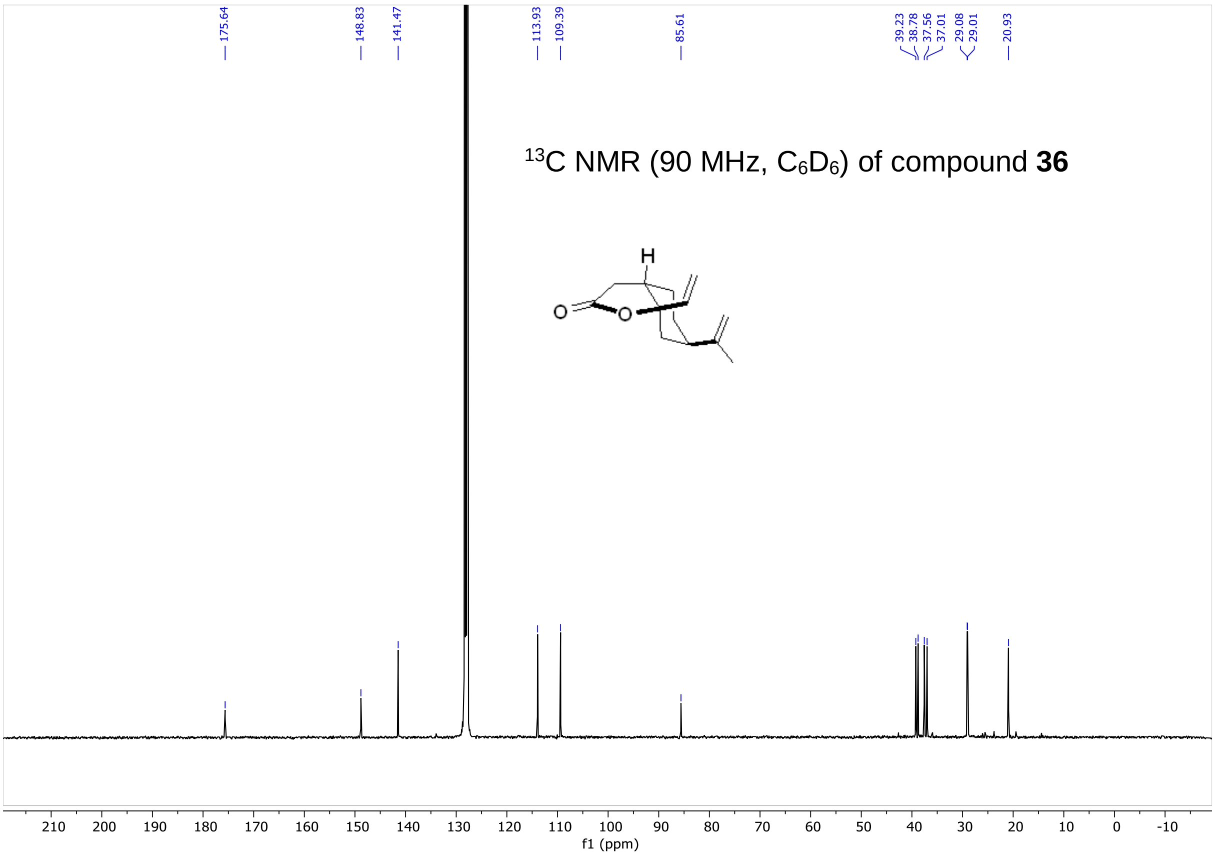
175.64
148.83
141.47
113.93
109.39
85.61
39.23
38.78
37.56
37.01
29.08
29.01
20.93
^{13}C NMR (90 MHz, C_6D_6) of compound **36**
H
O
O
210 200 190 180 170 160 150 140 130 120 110 100 90 80 70 60 50 40 30 20 10 0 -10
f1 (ppm)

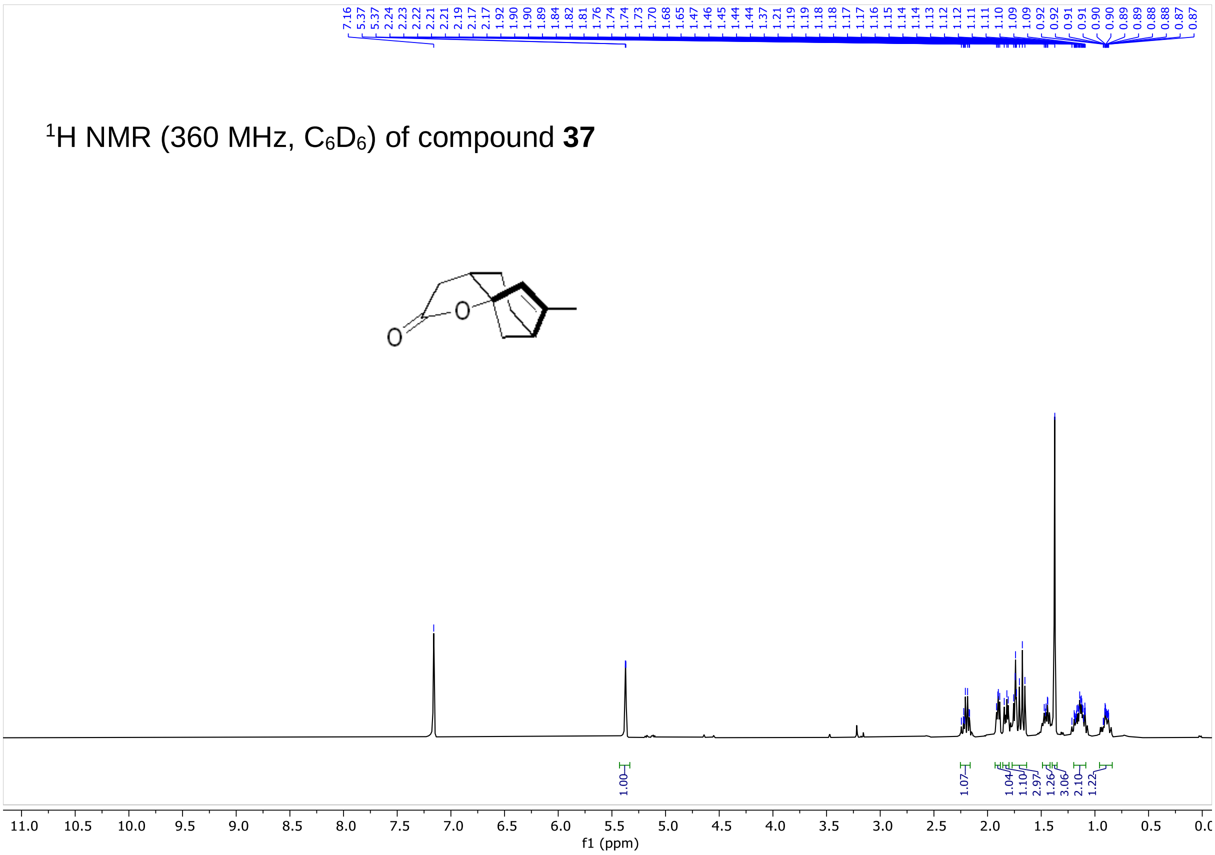
7.16 5.37 5.37 2.24 2.23 2.22 2.21 2.21 2.19 2.17 2.17 1.92 1.90 1.90 1.89 1.84 1.82 1.81 1.76 1.74 1.74 1.73 1.70 1.68 1.65 1.47 1.46 1.45 1.44 1.37 1.21 1.19 1.19 1.18 1.18 1.17 1.17 1.16 1.15 1.14 1.14 1.13 1.12 1.12 1.11 1.11 1.10 1.09 1.09 0.92 0.92 0.91 0.91 0.90 0.90 0.89 0.89 0.88 0.88 0.87 0.87
^{1}H NMR (360 MHz, C_6D_6) of compound **37**
O
O
1.00
1.07
1.04
1.10
2.97
1.26
3.06
2.10
1.22
11.0 10.5 10.0 9.5 9.0 8.5 8.0 7.5 7.0 6.5 6.0 5.5 5.0 4.5 4.0 3.5 3.0 2.5 2.0 1.5 1.0 0.5 0.0
f1 (ppm)

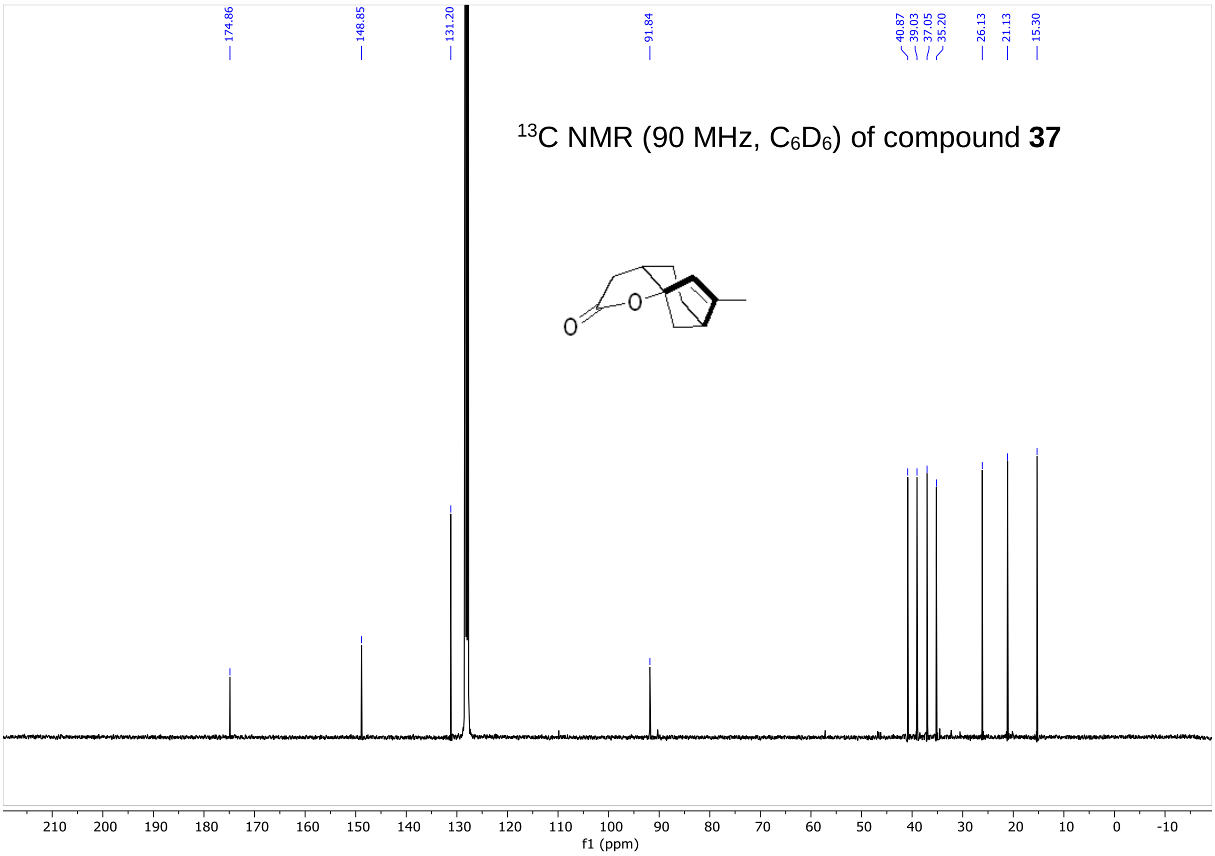

^{13}C NMR (90 MHz, C_6D_6) of compound **37**

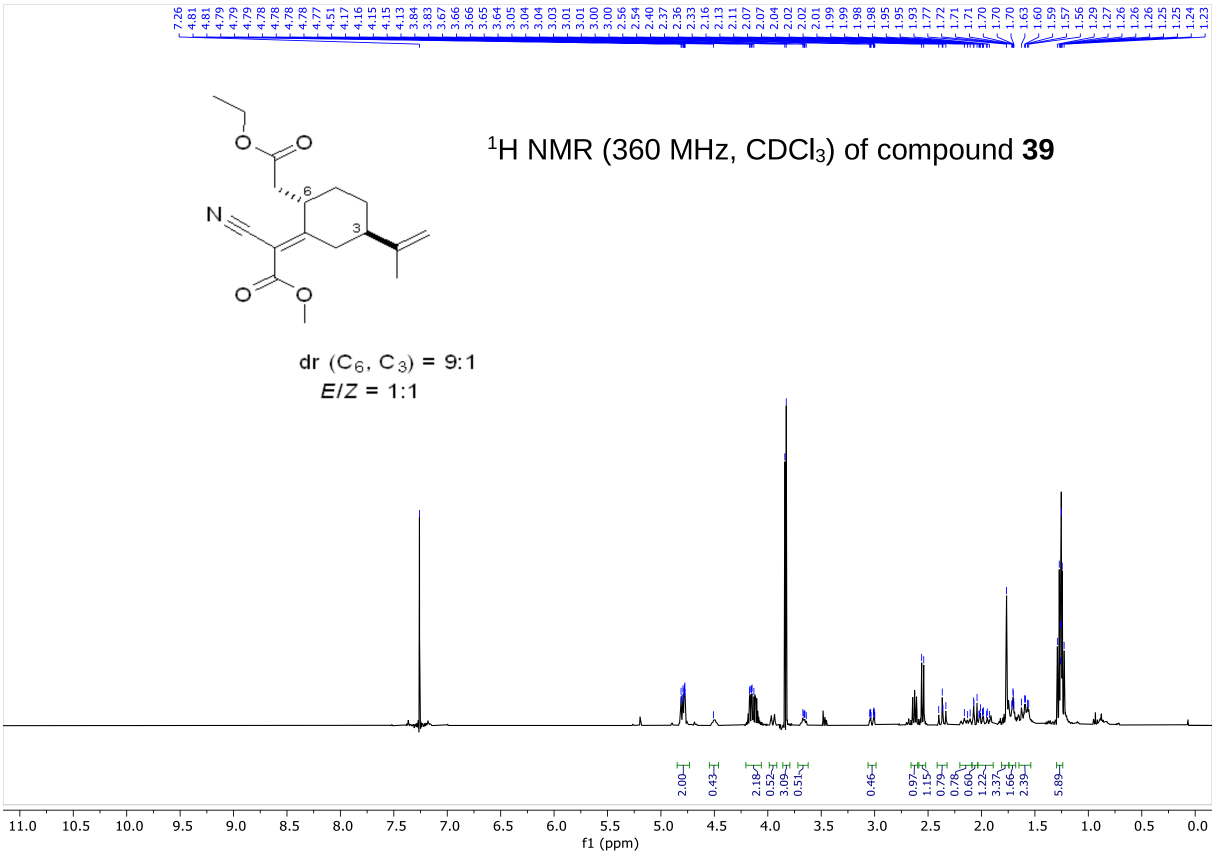

^{1}H NMR (360 MHz, $CDCl_3$) of compound **39**

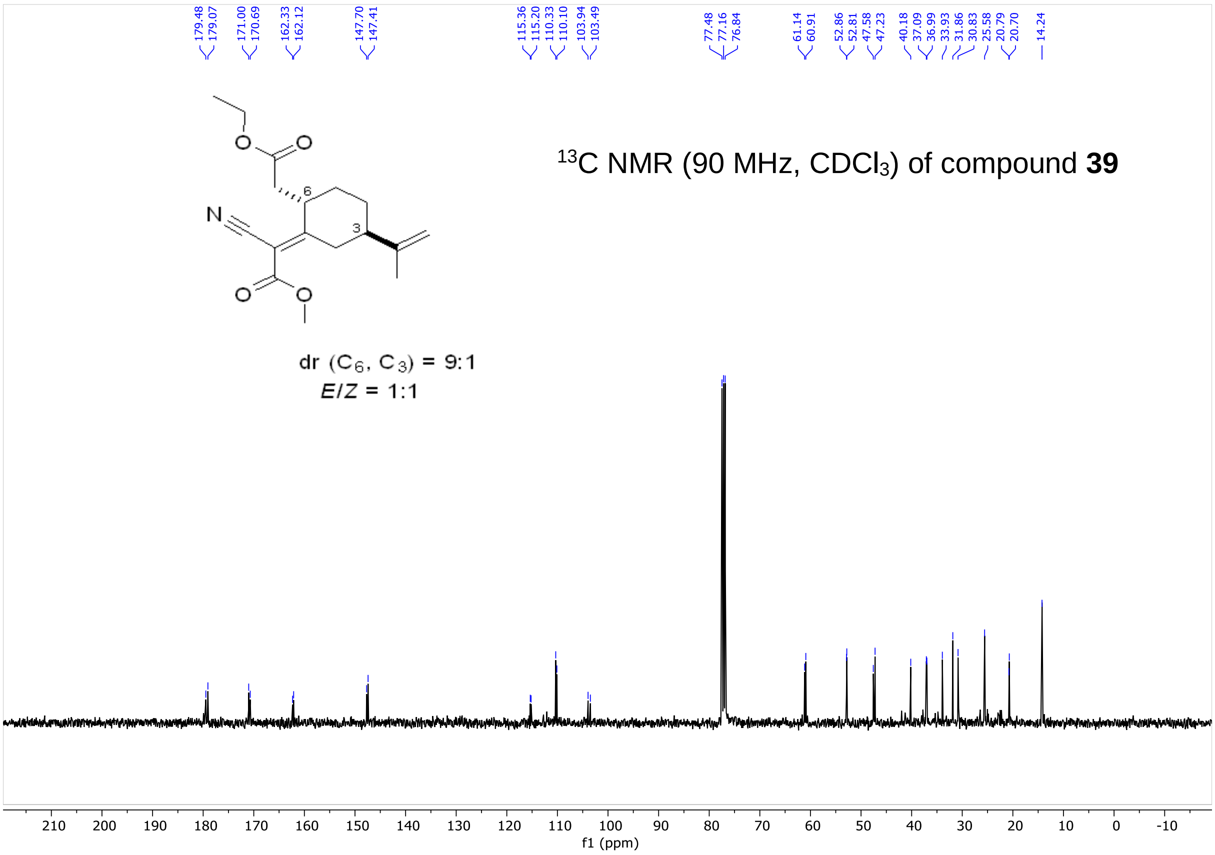
179.48
179.07
171.00
170.69
162.33
162.12
147.70
147.41
115.36
115.20
110.33
110.10
103.94
103.49
77.48
77.16
76.84
61.14
60.91
52.86
52.81
47.58
47.23
40.18
37.09
36.99
33.93
31.86
30.83
25.58
20.79
20.70
14.24
13C NMR (90 MHz, CDCl3) of compound 39
dr (C6, C3) = 9:1
E/Z = 1:1
f1 (ppm)

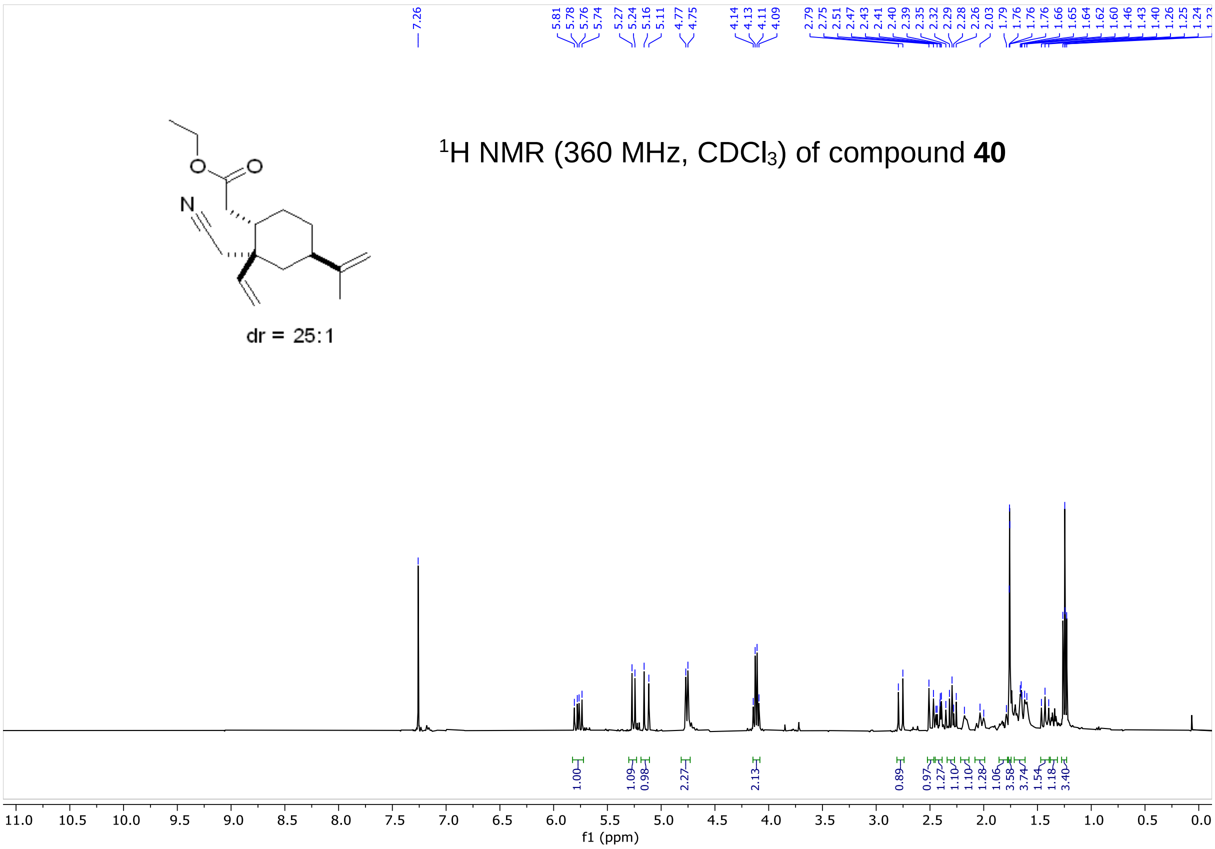
1H NMR (360 MHz, CDCl3) of compound 40
dr = 25:1
f1 (ppm)

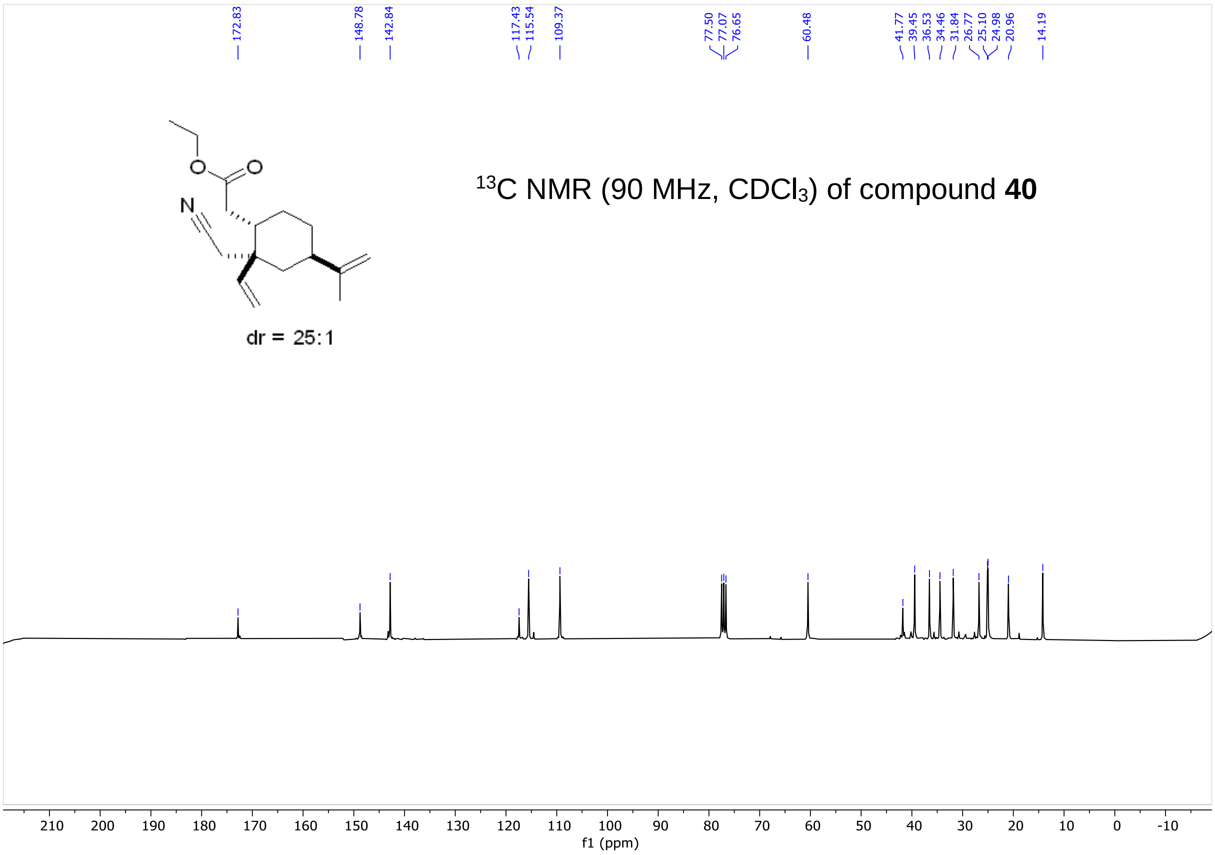

^{13}C NMR (90 MHz, $CDCl_3$) of compound **40**

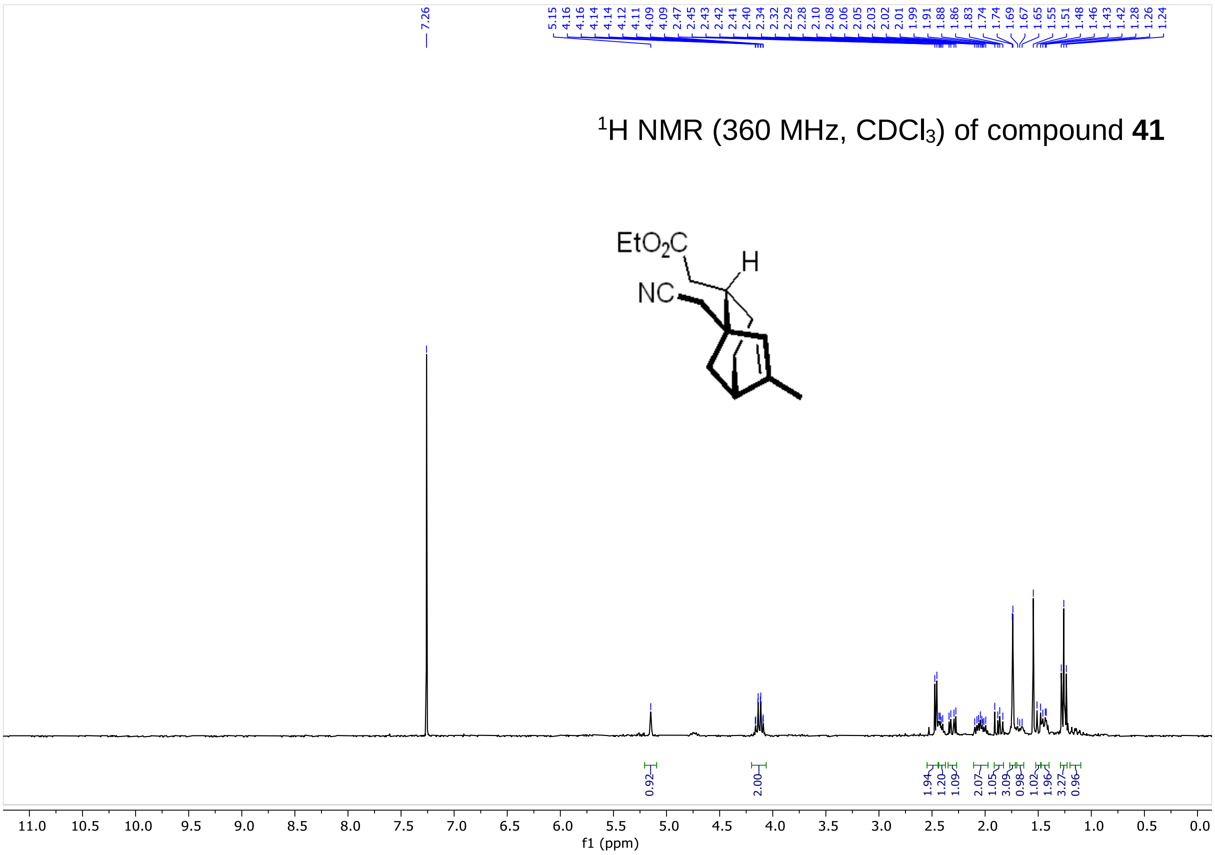

^{1}H NMR (360 MHz, $CDCl_3$) of compound **41**

^{13}C NMR (90 MHz, $CDCl_3$) of compound **41**

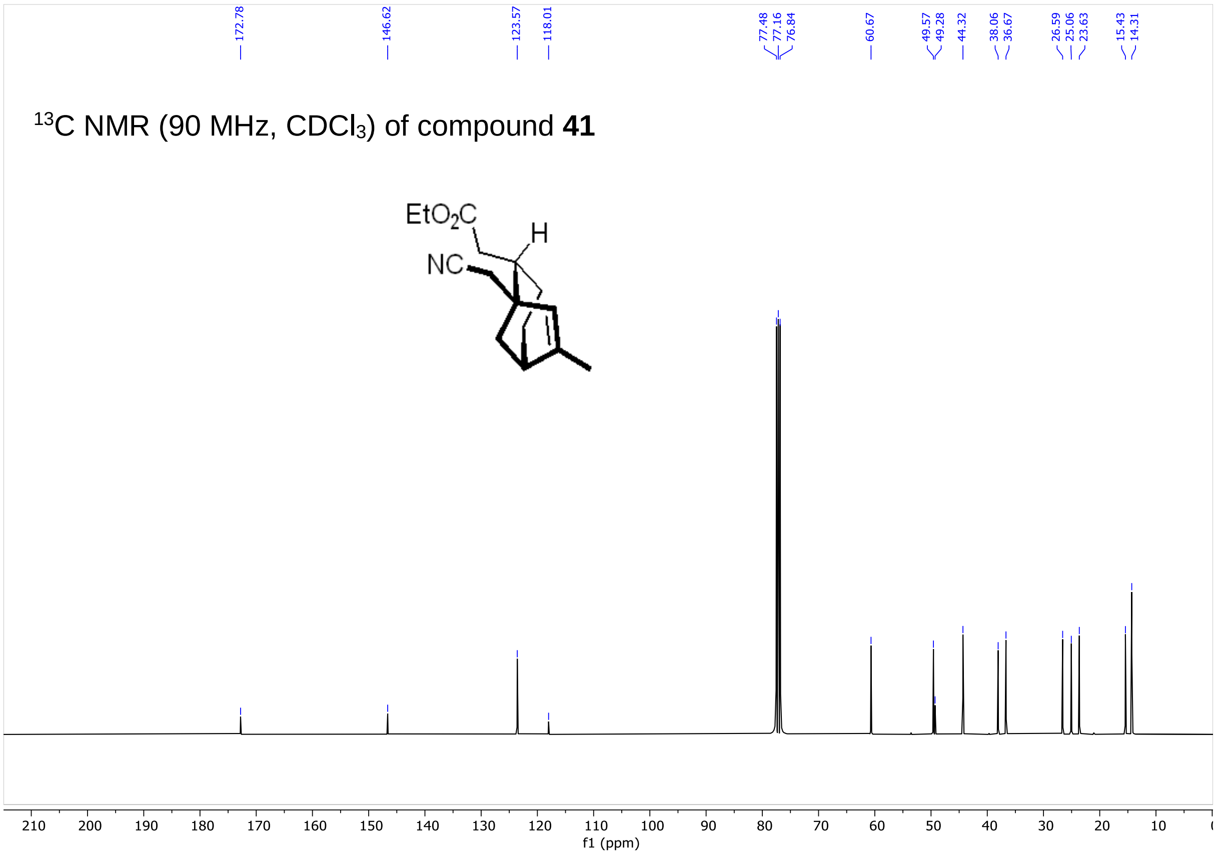

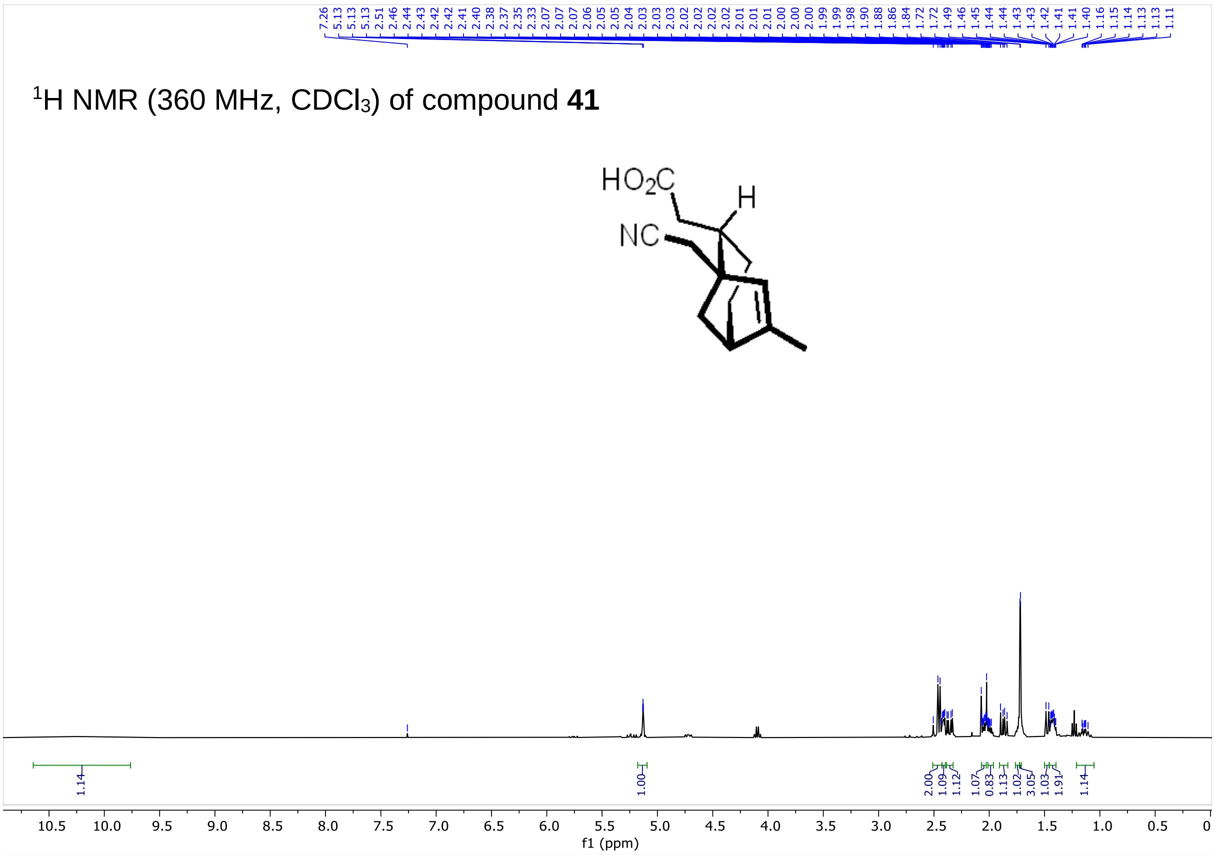

^{1}H NMR (360 MHz, $CDCl_3$) of compound **41**

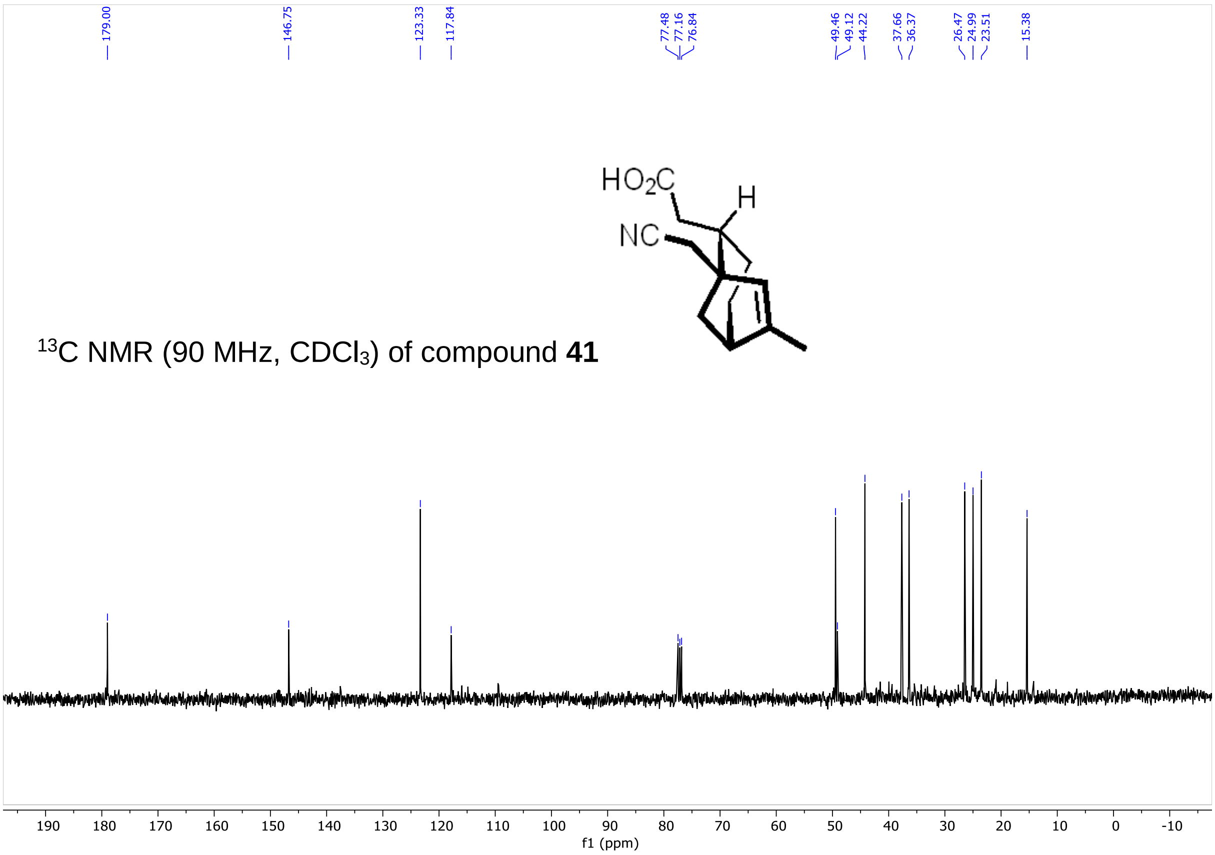

^{13}C NMR (90 MHz, $CDCl_3$) of compound **41**

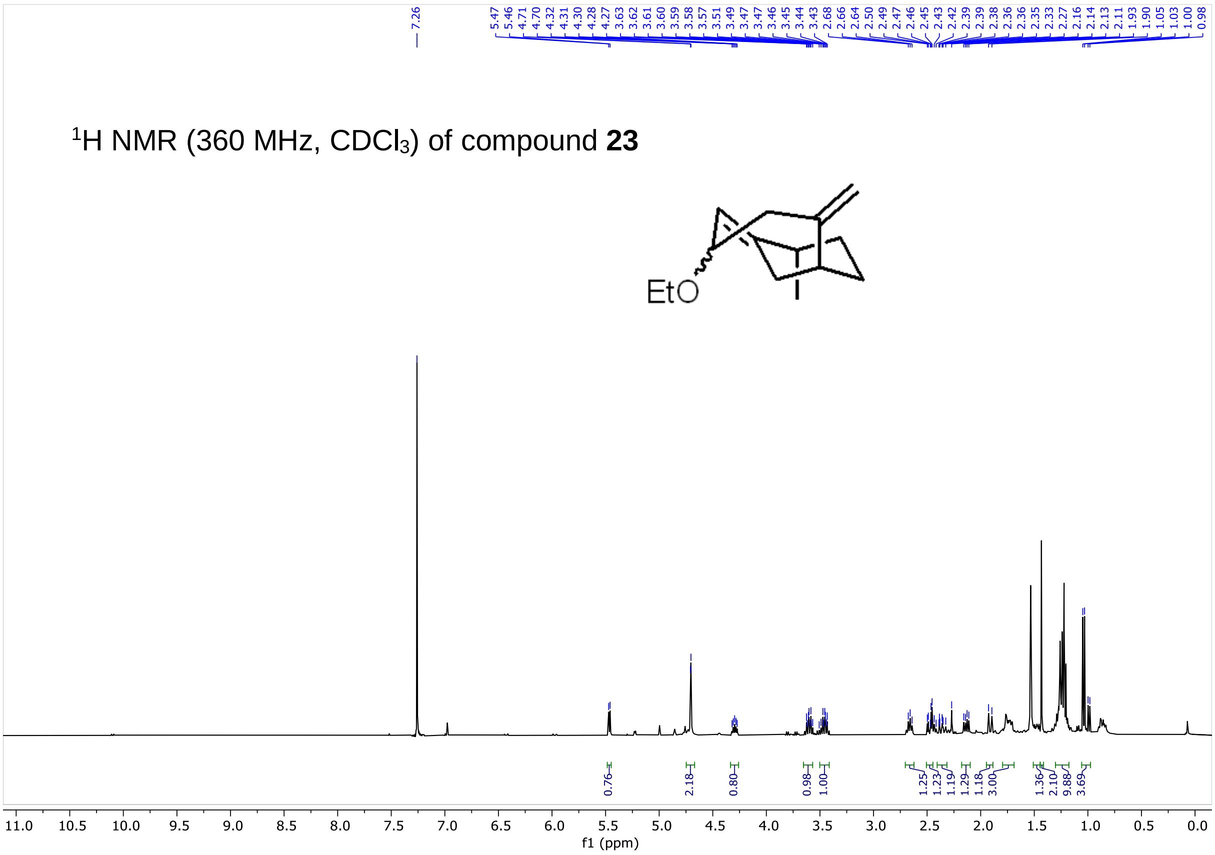

^{1}H NMR (360 MHz, $CDCl_3$) of compound **23**

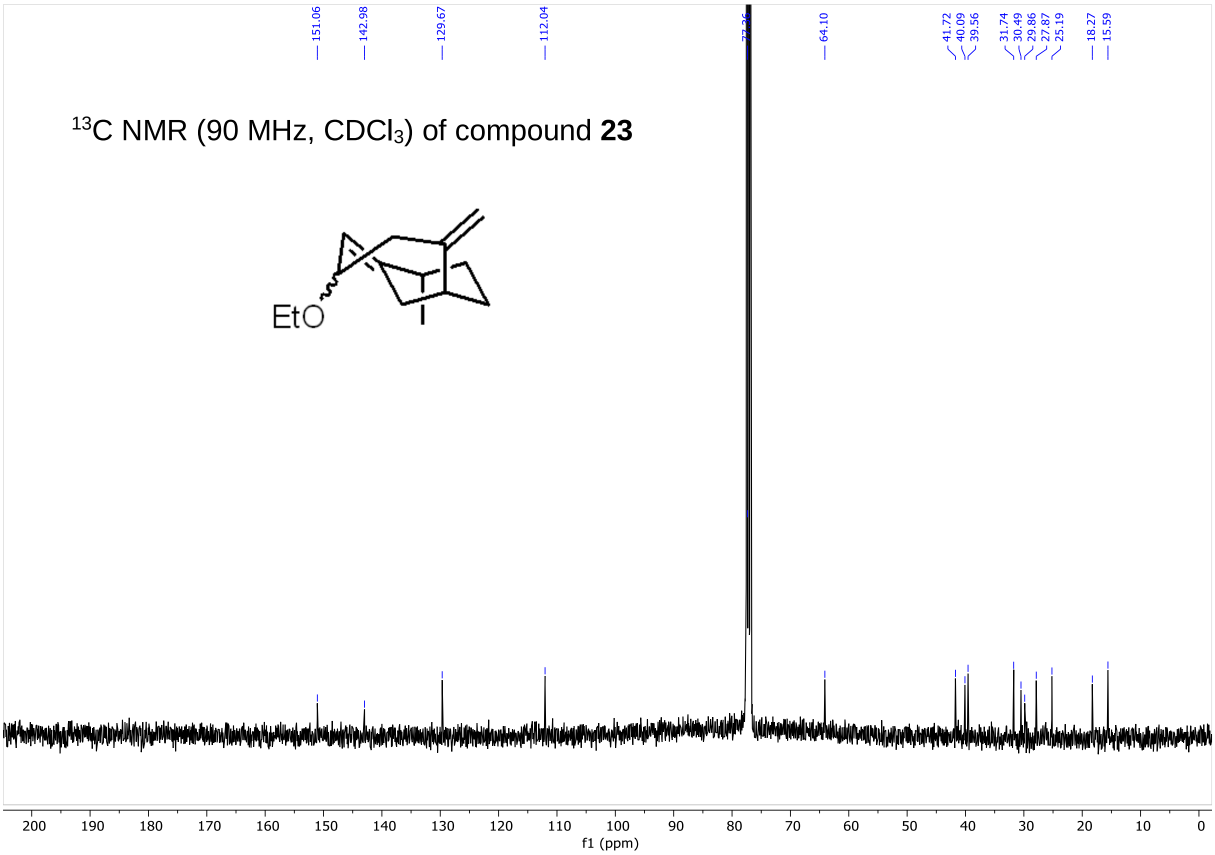

^{13}C NMR (90 MHz, $CDCl_3$) of compound **23**

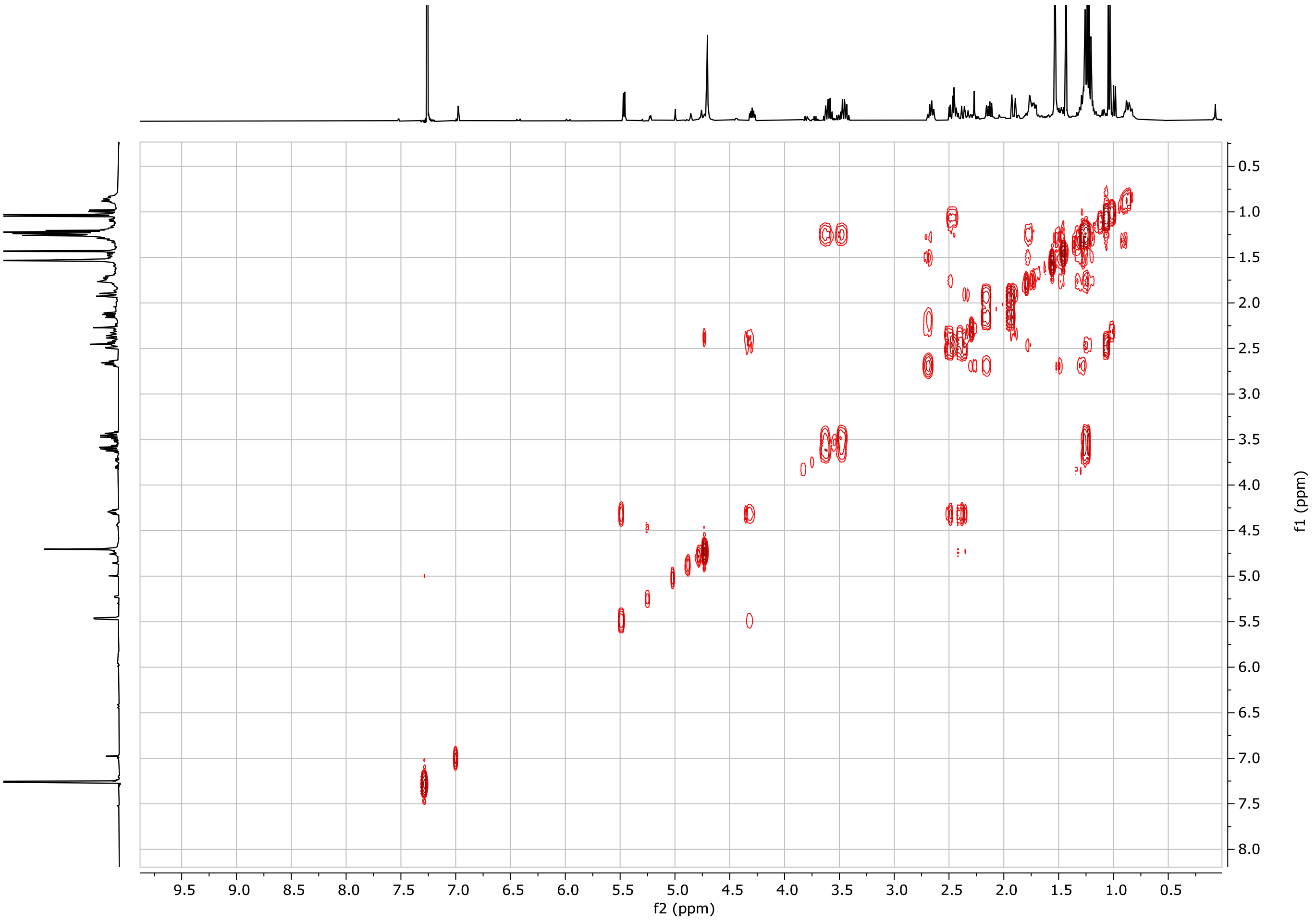

COSY spectrum of **23** (in CDCl3)

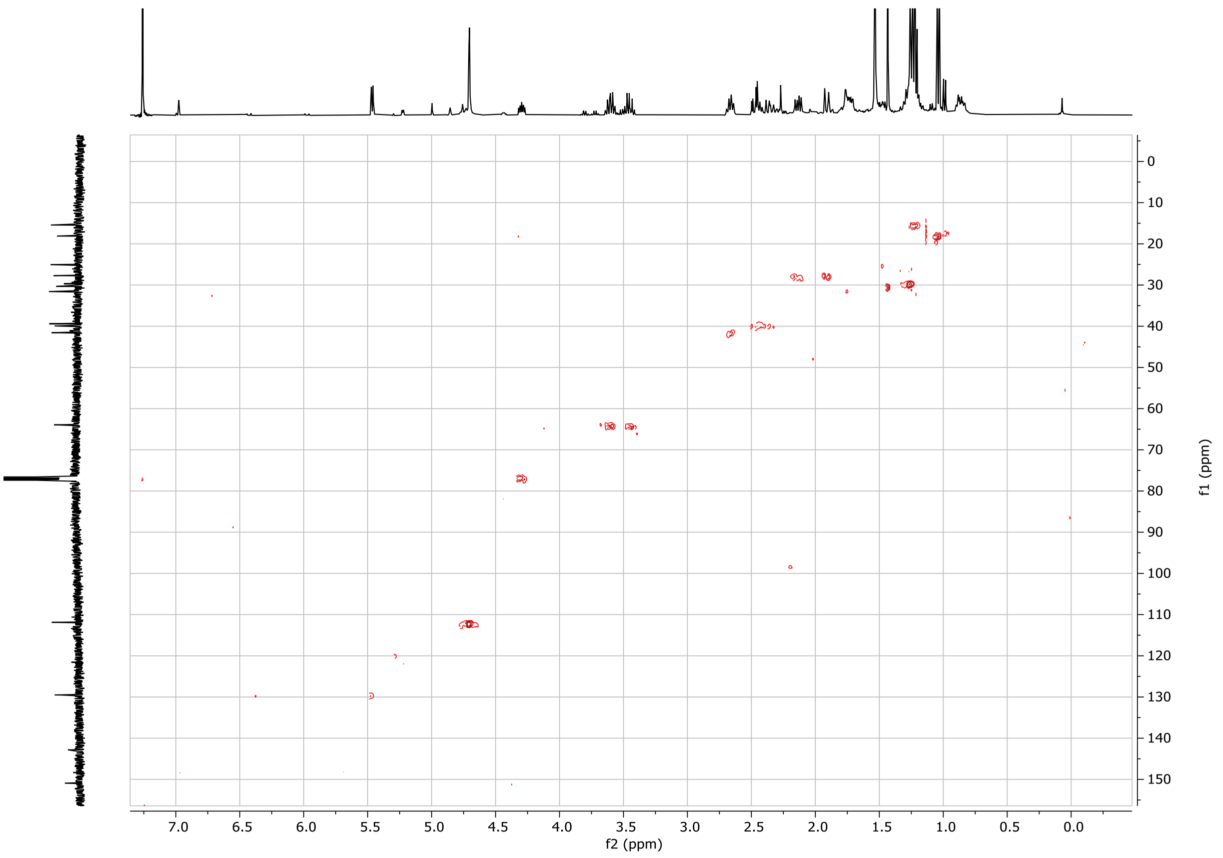

HSQC spectrum of **23** (in CDCl3)

References

[1] T. Kang, Y. Kim, D. Lee, Z. Wang, S. Chang, "Iridium-Catalyzed Intermolecular Amidation of sp3 C–H Bonds: Late-Stage Functionalization of an Unactivated Methyl Group" *J. Am. Chem. Soc.* **2014,** *136*, 4141–4144.

[2] O. Hauenstein, M. Reiter, S. Agarwal, B. Rieger, A. Greiner, "Bio-based polycarbonate from limonene oxide and CO2 with high molecular weight, excellent thermal resistance, hardness and transparency "*Green Chem.* **2016**, *18*, 760–770.

[3] M. Uroos, W. Lewis, A. J. Blake, C. J. Hayes, "Total Synthesis of (+)-Cymbodiacetal: A Re-evaluation of the Biomimetic Route" *J. Org. Chem.* **2010**, *75*, 8465.

[4] D. S. Pisoni, D. Gamba, C. V. Fonseca, J. S. da Costa, C. L. Petzhold, E. R. de Oliveira, M. A. *Ceschi,* E. R. de Oliveira, M.A Ceschi, "InCl3/NaClO: a reagent for allylic chlorination of terminal olefins" *J. Braz. Chem. Soc.* **2006***, 17,* 321-327.

[5] C. Henry, D. Bolien, B. Ibanescu, S. Bloodworth, D.C. Harrowven, X. Zhang, A. Craven, H. F. Sneddon, R.J. Whitby, "Generation and Trapping of Ketenes in Flow" *Eur. J. Org. Chem*. **2015**, *7,* 1491-1499.

[6] W. Nagata, Y. Hayase, "Formylolefination of carbonyl compounds" *J. Chem. Soc. C*, **1969**, *3*, 460-466.

[7] T. Gerke, C. Kropf, U. Huchel, A. Griesbeck, B. Porschen (Henkel AG), US 2018/0170849 A1, **2018**.

[8] R. L. Bassfield, K. F. Podraza, "Regiospecific Alkylation of 3-Substituted-2-cyclohexen-l-ones. Synthesis and Conformational Analysis of 6-(Carbethoxymethyl)-3-substituted-2-cyclohexen-l-ones" *J. Org. Chem.* **1989**, *54*, 5919–5922.

[9] J. Küppers, P. Becker, R. Jarling, M. Dörries, N. Cakić, M. Schmidtmann, J. Christoffers, R. Rabus, H. Wilkes, "Stereochemical Insights into the Anaerobic Degradation of 4-Isopropylbenzoyl-CoA in the Denitrifying Bacterium Strain pCyN1" *Chem. Eur. J.* **2019**, *25*, 4722.
